# Insights Into Disability and Disability Progression in People With Multiple Sclerosis Using Large‐Scale Healthcare Data

**DOI:** 10.1111/ene.70124

**Published:** 2025-03-30

**Authors:** Onur Dereli, Jochen Behringer, Achim Berthele, Alexander Hapfelmeier, Bernhard Hemmer, Christiane Gasperi

**Affiliations:** ^1^ Department of Neurology, TUM School of Medicine and Health, TUM University Hospital Technical University of Munich Munich Germany; ^2^ AOK Bayern Munich Germany; ^3^ Institute of AI and Informatics in Medicine, TUM School of Medicine and Health, TUM University Hospital Technical University of Munich Munich Germany; ^4^ Institute of General Practice and Health Services Research, TUM School of Medicine and Health, TUM University Hospital Technical University of Munich Munich Germany; ^5^ Munich Center for Health Economics and Policy Munich Germany; ^6^ Munich Cluster for Systems Neurology (SyNergy) Munich Germany

**Keywords:** disability progression, healthcare data, machine learning, multiple sclerosis, prediction

## Abstract

**Background:**

Identifying predictors for disability progression is crucial for managing multiple sclerosis (MS). This study aims to explore levels of disability and informative factors for disability progression in people with MS (PwMS) using healthcare data without detailed clinical information.

**Methods:**

We conducted a case–control/cohort study on data from Bavaria's largest health insurance organization. The dataset included records of assistive devices, nursing care, sick leaves, rehabilitation, drug therapies, and diagnoses for individuals with MS, Crohn's disease (CD), rheumatoid arthritis (RA), and controls (CTR) without these diseases. We used generalized linear models to compare healthcare service utilization between MS and other cohorts. A gradient‐boosting algorithm identified informative healthcare‐related factors associated with disability progression in PwMS, defined by increased nursing care utilization.

**Results:**

PwMS (*N* = 11,961) demonstrated higher healthcare utilization than CD (*N* = 21,884), RA (*N* = 105,450), and CTR (*N* = 82,677) groups, even at young ages. Besides expected risk factors like age, smoking, diabetes, and psychiatric disorders, the prediction algorithm revealed that PwMS with specific gynecological disorders, upper tract infections, asthma, and thyroiditis were less likely to need higher levels of nursing care.

**Conclusions:**

Leveraging healthcare data allows for an objective assessment of disability in PwMS and can identify informative factors for disability progression. Our approach can be applied to studies on disease progression in large cohorts without detailed clinical data and can be adapted to other diseases, disability measures, and healthcare systems. Higher utilization of healthcare resources even at young ages revealed an unmet need for improved treatment and management strategies for young adults with MS.

## 1 Introduction

Multiple sclerosis (MS) is a chronic inflammatory disease of the central nervous system and the most common non‐traumatic cause of acquired disability in young adults [1]. The clinical course is diverse, from nearly asymptomatic to highly active forms with rapid disability progression. Predicting the disease course as early as possible is crucial [2], and the search for predictors of disease progression is a vital question in MS research.

Several approaches using genomic, proteomic, or clinical information have been proposed to predict the disease progression of people with MS (PwMS) [3, 4, 5, 6, 7]. Collecting longitudinal data for these models is challenging; they are often sampled non‐periodically, and not all clinical measurements are performed at each patient visit. Therefore, such methods usually suffer from a limited number of individuals and missing information [8].

Most prediction algorithms use clinical measurement methods to depict the MS progression [3, 4, 5, 6, 7]. Quantitative measurement methods like the Expanded Disability Status Scale [9] (EDSS) have several limitations [10]. They require detailed clinical data, which is often unavailable, can be subjective, and are affected by the individual's daily condition. These challenges lead us to find alternative ways to determine disability progression.

For individuals with increasing disability, we can expect increasing utilization rates for assistive devices and remedies (e.g., walking aids, wheelchairs, physiotherapy), as well as more rehabilitation and sick leave requirements, or the establishment of nursing care. This study aimed to investigate whether disability progression of PwMS can be predicted using healthcare data.

Here, we first focused on revealing differences regarding healthcare factors between MS and other autoimmune diseases that affect young adults, Crohn's disease (CD) and rheumatoid arthritis (RA), and a control group (CTR) with none of these diseases. Following that, we utilized a gradient‐boosting‐based machine learning algorithm to identify informative healthcare factors in the disability progression of PwMS.

## 2 Methods

### 2.1 Data

Data from Allgemeine Ortskrankenkasse (AOK) Bayern, Bavaria's foremost health insurer, covers more than 35% of the population. Raw data include records for drug therapies, assistive devices, remedies for physiotherapy and ergotherapy (Table S1), nursing care levels, rehabilitation, sick leave reports, and ICD‐10 (The International Statistical Classification of Diseases and Related Health Problems, 10th revision [11]) codes (Table S2) for 18,263 individuals with MS, 22827 individuals with CD, 113921 individuals with RA, and 111,246 controls, collected quarterly between 2010 and 2020.

We categorized DMTs by effectiveness as moderate, high, and very high efficacy DMTs [12] (Table S3). We included the following assistive devices in our analyses: mobility‐related aids (walking aids, ambulances/vehicles, mobility aids, care aids for more independent living/mobility), incontinence‐related aids, nursing items, visual aids, toilet aids, care aids to facilitate care, care aids for body care/hygiene, and for alleviating complaints. We used the residential region (urban or rural) information [13] in our prediction model, determined from the provided partial postal codes.

In Germany, individuals receive nursing care at varying levels depending on their health‐related needs, if they last at least 6 months (Table S4). The nursing care levels range from one (minor impairments of independence) to five (heaviest impairment of autonomy) [14] (Table S5). We compared nursing care levels across different cohorts and used nursing care levels of PwMS to define disability progression as the outcome of the prediction model.

### 2.2 Cohort Definition, Data Filtering, and Cleaning

We used ICD‐10 codes G35 for MS, K50 for CD, and M06 for RA to define the cohorts. We included individuals diagnosed with the respective code at least twice in an ambulatory care unit in different quarterly periods or at least once in inpatient care. We applied thorough data filtering and cleaning, considering reliability and specialist information for diagnoses, possible demyelinating diseases in CTR, insurance status, and observation duration. The details of the filtering process can be found in Supporting Information. The final dataset included 11,961 individuals with MS, 21,884 with CD, 105,450 with RA, and 82,677 controls (Table 1).

**TABLE 1:** The summary of cohorts used in the analyses.

Phenotype	Number of patients	Age (mean [sd])	Sex (Female)	Observation in days (mean [sd])
Multiple sclerosis	11,961	49.30 [14.00]	69.67%	3318 [745]
Crohn's disease	21,884	51.91 [17.64]	56.48%	3226 [839]
Rheumatoid arthritis	105,450	69.46 [15.31]	69.58%	3244 [819]
Controls	82,677	52.28 [15.90]	70.13%	3041 [1007]

### 2.3 Generalized Linear Models for Statistical Comparisons of MS Versus Control Cohorts

We utilized generalized linear models (GLMs) to analyze differences in healthcare utilization between MS, CD, RA, and CTR. For binary outcomes, we utilized quasibinomial distributions, and for count data, we used negative binomial distributions. We used the quasibinomial distribution to address overdispersion in binary outcomes, providing a better fit to the data by adjusting the observed variability [15, 16].

We included disease phenotype, age, sex, and insurance period as covariates in the models. The observation length was utilized as a regularization parameter to adjust the models accordingly. We constructed the GLMs using the following formula:

$$\begin{array}{c} \text{Outcome}∼\text{Phenotype}+\mathrm{Age}+\mathrm{Sex}+\text{InsurancePeriod}\\ +\text{offset}\left(\log\left(\text{ObservationDuration}\right)\right) \end{array}$$

Using the model coefficients obtained from the GLMs constructed for each healthcare parameter either using quasibinomial or negative binomial distributions, we calculated odds ratios (ORs) and corresponding 95% confidence intervals (CIs) and obtained significance levels (*p*‐values) of each covariate. We used MS as the reference phenotype in the models. Consequently, odds ratios lower than one for CD, RA, and CTR indicate higher odds of observing the relevant outcome parameters in MS, while odds ratios higher than one indicate lower odds of observing the relevant outcome parameters in MS. To increase the interpretability, we report the inverse odds ratios (1/OR), where higher values represent higher odds of observing the relevant outcome parameters in MS. For each model, we report Bonferroni‐adjusted *p*‐values in cases involving multiple statistical comparisons. To analyze sick leave, we performed GLM analysis on a subset of individuals within active working ages (ages 25 to 55), as we could not accurately determine individuals' unemployment or retirement status. We also conducted separate analyses for different age groups (20–29, …,70–79, 80+) for each outcome.

### 2.4 Prediction Model

#### 2.4.1 Feature Set Used in Prediction Model

We employed longitudinal healthcare records to predict disability progression defined by a nursing care level increase. In our training model, we utilized a 3‐year patient trajectory and quarterly records with missing information of up to 30% of the observation period (see Supporting Information for the details). Table 2 lists all 1119 features included in our model.

**TABLE 2:** List of all features used in the prediction model.

Feature	Variable type	Values
Sex	Categorical	1 = female, 2 = male
Age at given quarter	Continuous	[20,107]
Frequency of receiving rehabilitation	Continuous	≥ 0
Duration of rehabilitation treatment in days	Continuous	≥ 0
Number of doctor visits made at the given quarter	Continuous	≥ 0
Year of the observation for the given quarter	Categorical	{2010, …,2019}
Order of the observation for the given quarter	Categorical	{1 = earliest record, …, 12 = latest record}
MS subtype: Last known diagnosis of an individual at the given quarter	Categorical	(1 = Unspecified, 2 = RRMS, 3 = SPMS, 4 = PPMS)
DMT level: Level of DMT prescribed to an individual at the given quarter (Table S3)	Categorical	{1 = None, 2 = Moderate Efficacy, 3 = High Efficacy, 4 = Very high efficacy}
Residential region: Information about the last known residential region of an individual	Categorical	{1 = Urban, 2 = Rural}
Usage of an assistive device group at the given quarter: mobility‐related aids, incontinence‐related aids, nursing items, visual aids, toilet aids, care aids to facilitate care, care aids for body care/hygiene and for alleviating complaints	Binary	Yes, No
Diagnosis of a disease at the given quarter. List of ICD‐10 Codes can be found in Table S2	Binary	Yes, No
Usage of a physiotherapy or ergotherapy remedy. The list of physiotherapy and ergotherapy treatments can be found in Table S1	Binary	Yes, No

Abbreviations: DMT, disease modifying therapy; ICD‐10, The International Statistical Classification of Diseases and Related Health Problems, 10th revision; MS, multiple sclerosis; PPMS, primary‐progressive MS; RRMS, relapsing–remitting MS; SPMS, secondary‐progressive MS.

#### 2.4.2 Prediction Task Definition

Our model predicts the disability progression of PwMS 2 years after the observation period by taking 3 years of healthcare records of N PwMS, denoted as X, and the disability progression of those individuals 2 years after the last observation, denoted as Y, as input (Figure S1). We classified individuals who required nursing care at level two or lower at the end of the observation period ($t_{0}$) but required nursing care at level three or higher 2 years after $t_{0}$ as individuals with disability progression, and individuals who still required nursing care at level two or lower at $t_{0}+2$ as individuals without disability progression. We defined $t_{0}$ as follows: If an individual required nursing care at level three or higher, we picked the last quarter before reaching level three for the first time as the end of the observation period, which ensures that individual was also receiving nursing care level three or higher after 2 years. If not, we picked 2 years before the last observation as $t_{0}$. We excluded individuals with nursing care level three or higher during the observation period. The resulting dataset included 8360 PwMS, 687 of whom showed an increase in the nursing care level.

#### 2.4.3 Learning Algorithm

We trained our prediction model using a gradient‐boosting tree‐based learning algorithm, LightGBM [17], implemented via LightGBM's R package (v3.2.2) [18].

#### 2.4.4 Experimental Settings

Data include multiple records per individual, deviating from the independent and identically distributed (IID) structure due to correlated instances. We split the non‐IID structured data as follows: We divided the dataset into training and test groups by randomly picking 80% of the individuals for training and 20% for the test set. By applying a person‐level splitting procedure, we kept all samples belonging to the same individual either in training or test sets. We tried to keep the ratio between individuals who showed disability progression and those who did not the same in both sets. We repeated this process 50 times to obtain more robust predictive results and tuned the hyperparameters using 4‐fold cross‐validation on the training set, keeping all samples of an individual in the same fold. We tuned and updated one hyperparameter at a time by selecting the best value for the average area under the precision‐recall curve (AUPR) through cross‐validation. The hyperparameters and their search range are listed in Table S6.

We reported the predictive performance on the test partition over 50 replications using the area under the receiver operating characteristic (AUROC) and the AUPR curves, F1, and Average Balanced Accuracy metrics. We compared our predictive performance with: (i) a LightGBM algorithm trained on data including the same individuals with feature columns created for each time point, preserving IID structure since it includes one sample per individual; and (ii) GPBoost [19], a LightGBM‐based Gaussian process and mixed effects model proposed for longitudinal data with non‐IID structure. The GPBoost algorithm used the same data as our approach, and both algorithms were trained using the same experimental settings.

We calculated the contribution of each feature to the prediction by utilizing Shapley values [20] obtained through the model trained on our entire dataset. Details on performance metrics and the Shapley method can be found in the Supporting Information.

## 3 Results

### 3.1 Healthcare Needs of Individuals With MS Compared to Those With CD, RA, and Controls

**FIGURE 1:**
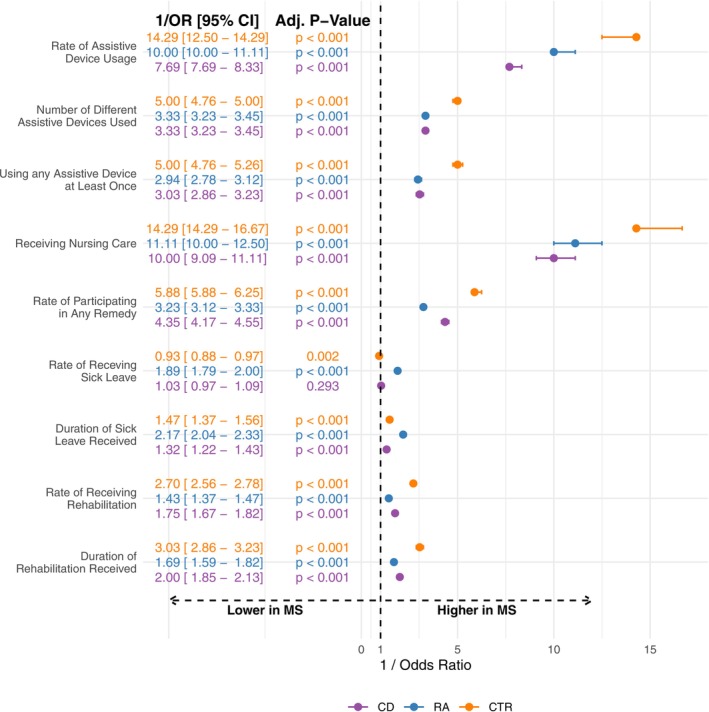
The comparisons of MS, CD, RA, and CTR, with respect to frequencies of utilizing each healthcare parameter. The odds ratios, their corresponding 95% confidence intervals, and adjusted *p*‐values for model covariates were obtained using generalized linear models constructed for each healthcare parameter. MS was used as the base phenotype for all models, meaning that higher values for (1/odds ratios) indicate a higher likelihood of observing the related outcome in MS. Adj, adjusted; CD, Crohn's disease; CTR, controls; MS, multiple sclerosis; RA, rheumatoid arthritis.

PwMS showed a significantly higher need for assistive devices, nursing care, remedies, rehabilitative treatments, and sick leaves than all control groups (Figure 1). Utilization of all healthcare parameters was considerably higher in MS for all age groups, including young individuals (Table S7).

The odds of using assistive devices were significantly higher in MS compared to CD, RA, and CTR, with overall usage being 7.69, 10, and 14.29 times higher, respectively. MS also showed three to five times higher odds for different types of assistive devices across all cohorts. Specifically, for ages 30–39, MS had 1.8 and 2.7 times higher odds than CD and CTR, respectively (Figure S2). Usage of mobility, incontinence, and care‐related aids was notably higher in MS across almost all age groups (Figure S3, Table S8). In the 30–39 age group, the odds of using mobility aids were 1.85 and 2.56 times higher for MS than CD and CTR, but similar to RA (1/OR = 1.1, 95% CI = [0.94, 1.28]). Odds for incontinence aids were three, six, and eight times higher in MS than in CD (1/OR = 3.33), RA (1/OR = 5.88), and CTR (1/OR = 8.33), respectively.

Nursing care utilization was more frequent in MS than CD (1/OR = 10), RA (1/OR = 11.11), and CTR (1/OR = 14.29) (Figure 1). For ages 30–39, MS showed three to six times higher odds compared to CD (1/OR = 5.6), RA (1/OR = 3.3), and CTR (1/OR = 5.6) (Figure S4). This difference was consistent across all nursing care levels, especially for higher levels (Figure S5). For ages 30–39, the odds of requiring level two nursing care were 8.13, 13.33, and 4.27 times higher for MS than CTR, CD, and RA, respectively (Table S9). After age 40, the odds of receiving nursing care remained significantly higher for MS across all levels compared to other cohorts.

The odds of participating in remedies were higher in MS compared to CD (1/OR = 4.35), RA (1/OR = 3.23), and CTR (1/OR = 5.88) (Figure 1). For ages 30–39, the odds of receiving physiotherapy were 2.27, 1.28, and 3.70 times higher for PwMS compared to CD, RA, and CTR (Figure S6A). Specifically, between ages 30 and 39, the odds of receiving ergotherapy were approximately 5.3, 2, and 14.3 times higher in MS than in CD, RA, and CTR, respectively. However, there was no significant difference compared to RA after adjusting for multiple testing (adjusted *p*‐value = 1) (Figure S6B).

The odds of receiving rehabilitative treatments were also higher in MS than in CD (1/OR = 1.75), RA (1/OR = 1.43), and CTR (1/OR = 2.70) (Figure 1). Furthermore, the odds of a longer duration of rehabilitation were 2, 1.69, and 3.03 times higher in MS compared to CD, RA, and CTR, respectively.

While the odds of receiving sick leave were similar for CD compared to PwMS (1/OR = 1.03, 95% CI = [0.97, 1.09]) and were slightly higher for CTR (1/OR = 0.93), these odds were lower for the individuals with RA (1/OR = 1.89) compared to PwMS (Figure 1). However, the odds of receiving longer sick leaves were higher for PwMS compared to individuals with CD (1/OR = 1.32), RA (1/OR = 2.17), and CTR (1/OR = 1.47).

### 3.2 Informative Healthcare Factors in Predicting Disease Progression of PwMS

Our algorithm predicted the need for higher levels of nursing care after 2 years using the healthcare data with 0.94 mean AUROC and 0.71 mean AUPR values, outperforming baseline performance levels (i.e., AUROC = 0.5 and AUPR = 0.082 for the random classifier). Mean F1 and the Average Balanced Accuracy values are 0.52 and 0.87, respectively, indicating better predictions compared to random classifiers. Figure S7 and Table S10 summarize the predictive performance over 50 replications.

**FIGURE 2:**
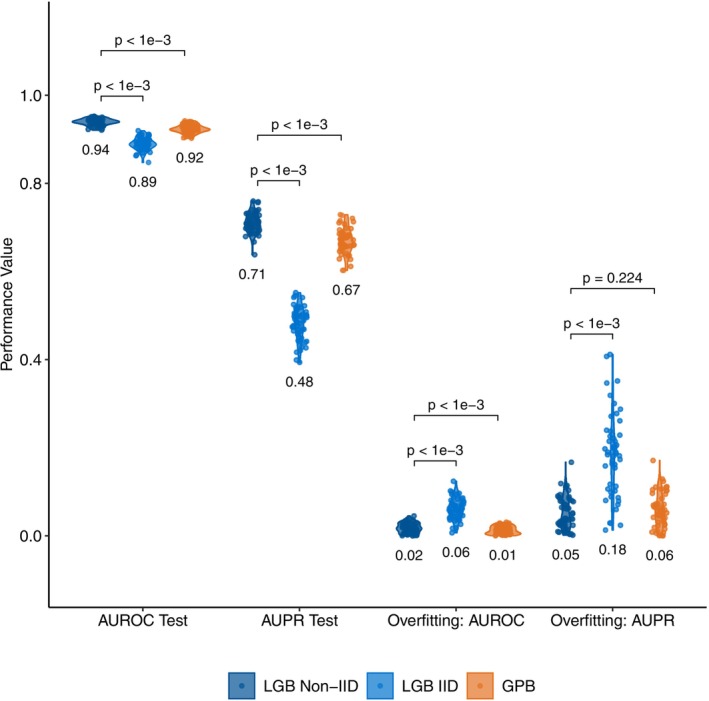
Predictive performance comparisons. Predictive performances of our approach (LGB Non‐IID) compared to the LightGBM algorithm trained on the IID version of our data (LGB IID) and the LightGBM‐based Gaussian process and mixed effects model (GPBoost) specifically proposed for longitudinal data with non‐IID. structure. The first two violin plots show the AUROC and AUPR values over 50 replications on the test set. The levels of overfitting with respect to AUROC and AUPR values were obtained by subtracting the performance levels on the test set from the training set for each replication, where a lower difference indicates better generalizability. The significance between the performances of the algorithms was assessed using two‐tailed paired *t*‐tests. Mean values for each measure over 50 replications are given under each corresponding violin plot. AUPR, the area under the precision‐recall curve; AUROC, area under the receiver operating characteristic curve; IID, independent and identically distributed.

Our approach (LGB Non‐IID in Figure 2) obtained better or comparable results against the IID version of our algorithm (LGB IID) and the LightGBM‐based Gaussian process and mixed effects model (GPBoost). Our approach obtained significantly higher AUROC and AUPR values on the test data than both algorithms and demonstrated stronger generalization than LGB IID, with similar generalization to GPBoost, as evidenced by smaller AUROC and AUPR differences between training and test sets (Figure 2).

**FIGURE 3:**
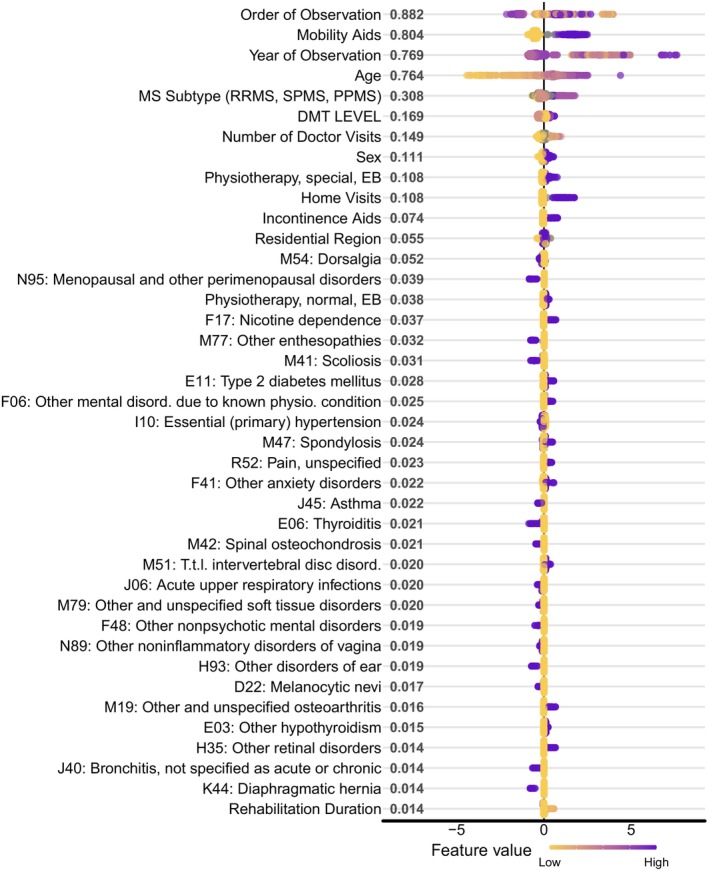
Feature contributions on predicting disability progression. Shap summary plot shows the contributions of top‐ranked model features on disability progression. The mean absolute Shapley values are given next to each corresponding feature in the order of their relative contribution to the prediction task. Each row of the beeswarm plot represents the degree of impact for the corresponding feature, and the gradient color indicates the original value of the given feature (i.e., yellow for the lowest, magenta for the highest values). Features with positive Shapley values have an increasing impact on disability progression, while those with negative values have the opposite effect. The higher the mean absolute Shapley value, the higher the contribution of the feature to the model. Disord, disorders; physio, physiological; T.t.l, thoracic, thoracolumbar, and lumbosacral. Values of the categorical features: DMT Levels: 1 = None, 2 = Moderate Efficacy, 3 = High Efficacy, 4 = Very High Efficacy; MS Subtypes: 1 = Unspecified, 2 = RRMS, 3 = SPMS, 4 = PPMS; Residential Region: 1 = Urban, 2 = RuralSex: 1 = Female, 2 = Male.

Figure 3 illustrates the top 40 model features ranked by mean absolute Shapley values, reflecting their relative contribution to the model. The full list of the feature contributions is given in Table S11 (details on Shapley values in Experimental Settings and Supporting Information).

Unsurprisingly, age was among the most significant risk factors affecting nursing care levels. Also, individuals diagnosed with secondary progressive MS (SPMS) or primary progressive MS (PPMS) were more likely to switch to nursing care level three or higher within 2 years. Mobility‐related aids and high‐efficacy DMTs were among the most crucial informative factors, while male sex was also a strong predictor for higher nursing care levels (Figure 3).

We found that ICD‐10 codes linked to a higher risk of disability progression included nicotine dependence (F17), type 2 diabetes mellitus (E11), mental and anxiety disorders (F06, F41), as well as scoliosis, spondylosis, and disc disorders (M41, M47, and M51). Additionally, individuals with unspecified pain (R52) were more likely to switch to a higher nursing care level.

Several ICD‐10 codes were linked to a lower risk of switching to higher nursing care levels, including bronchial asthma (J45) and thyroiditis (E06). Interestingly, two gynecological disorders—menopausal and other perimenopausal disorders (N95), and other noninflammatory disorders of the vagina (N89)—and two infections of the upper respiratory tract‐acute upper respiratory infections (J06) and bronchitis (J40)—were also associated with a lower risk of needing a higher nursing care level.

## 4 Discussion

We analyzed healthcare service usage of PwMS in Bavaria, focusing on assistive devices, remedies, nursing care, medical and rehabilitative treatments, incapacity for work, and diagnostic records. We compared PwMS to a control cohort as well as to individuals with either CD or RA, two other chronic autoimmune diseases. While their disease characteristics and trajectories differ, all three conditions are associated with disability accumulation and often require the use of varying healthcare factors [21, 22, 23, 24]. We investigated whether healthcare data can be used to predict disability progression and identify informative factors for disability progression in PwMS.

Analyses showed that although CD and RA can manifest at an early age and cause disability leading to high healthcare utilization [21, 22], PwMS need healthcare services more likely than CD and RA in almost all age groups. Even in young age groups, a relevant proportion of PwMS showed increased healthcare usage. To our knowledge, studies presenting differences between MS and other chronic autoimmune diseases regarding healthcare utilization are rare. The prominent use of healthcare factors among PwMS, even at an early age, emphasizes the need to diagnose and treat PwMS as early as possible to prevent disease progression causing disability.

Using nursing care to indicate disability is uncommon, as most studies focus on EDSS progression or progression to SPMS. We chose nursing care level two as the predictive threshold since level three exhibits severe independence impairment, often requiring extensive mobility device usage. In contrast, individuals at care level two can still move independently with assistive devices.

Since we introduced a new indicator for disease progression, a direct comparison with existing MS progression prediction algorithms was not possible. However, those algorithms reported AUROC and AUPR levels similar to ours, indicating that disability progression of PwMS can also be predicted without detailed clinical/*omic* data. Previous models often used genomic, proteomic, and detailed clinical data [3, 4, 5, 6, 7]. These are typically unavailable for large cohorts or suffer from high missingness and unstandardized recording. Assessing and predicting disability progression using healthcare data allows for analyzing large cohorts from different care settings without detailed clinical information.

Age, progressive MS, mobility and incontinence aids, physiotherapy, and very high efficacy DMTs are known risk factors for disease progression or associated with acquired disability [25, 26, 27, 28, 29, 30]. Our findings demonstrate that changes in nursing care requirements can indicate future disability progression as our model also identified those factors as informative. Additionally, —among other comorbidities—nicotine dependence, type 2 diabetes mellitus, and psychiatric comorbidities were associated with a higher nursing care level, aligning with studies linking these conditions to increased disability in PwMS [31, 32, 33].

Interestingly, we found ICD‐10 codes associated with a lower risk of needing a higher nursing care level, including two gynecological disorders—menopausal and other perimenopausal disorders and noninflammatory disorders of the vagina. Using a different dataset from Bavaria, we previously showed that gynecological disorders, including noninflammatory diseases of the female genital tract [34] were linked to reduced MS risk. Using different data and approaches, we found these disorders are both associated with lower MS risk and lower disability progression in PwMS. Our results hint at a relationship between gynecological disorders and MS susceptibility or the disease course that has yet to be understood.

Additionally, acute upper respiratory infections (J06) and bronchitis (J40) had a risk‐lowering association with disability progression. Our previous study indicated that different upper respiratory tract infections were less frequently observed in PwMS before disease onset [35], possibly. These findings may reflect an interaction between upper respiratory infections and immunomodulatory treatments increasing infection risk. They may also indicate a true negative association between specific infections and MS risk and disease progression, warranting further investigation to better understand these associations.

Lastly, bronchial asthma and thyroiditis were associated with a reduced risk of increasing disability. Although these comorbidities were found to be more common in PwMS compared to the general population [36, 37], they were not linked to a higher risk of disability in MS [38, 39]. It was previously described that MS tends to be less severe in individuals with such comorbidities [40, 41], indicating the necessity for further analysis of the association between lower disability progression risks in PwMS and these conditions.

Missing data is typical in longitudinal healthcare records due to irregular medical visits and incomplete insurance records. We addressed this limitation using a learning algorithm capable of handling such gaps. Diagnoses were not externally audited, and even with secured MS diagnoses by neurologists, we observed conflicting records. To mitigate this, we only included individuals with at least two cohort‐defining diagnoses, picked secured diagnoses when available, and excluded individuals with conflicting records. Additionally, the timing of first diagnoses was missing in the data, complicating analyses of pre‐ and post‐diagnosis differences and the effects of early DMT use on progression. Including sick leave data in our model yielded conflicting findings (i.e., higher likelihood of disability progression in PwMS with low sick leave days), likely because unemployed individuals do not receive sick leave reports. Due to the absence of employment status information, we excluded incapacity for work data from our prediction model.

In Germany, over 85% of the population has statutory health insurance, while the remainder is privately insured. AOK, the largest statutory insurer, covers approximately 35% of Bavaria's population and predominantly serves individuals with lower socioeconomic status and greater healthcare needs than those with private or other statutory health insurers [42, 43]. While our dataset enables robust analyses within this significant subgroup, we acknowledge that these differences may influence healthcare utilization, necessitating further investigation using multi‐insurer datasets for broader generalizability.

Overall, we demonstrated a higher utilization of healthcare resources like assistive devices, remedies, nursing care, rehabilitation, and sick leaves in PwMS compared not only to the general population but also to individuals with other chronic autoimmune diseases affecting young adults. Increased healthcare system utilization in young adults highlights the unmet need for the treatment of young adults with MS. We showed that healthcare data can identify informative factors for disability progression defined as increasing nursing care levels, offering an objective approach to assess disability and disease progression in PwMS. This approach can be applied to studies on disease progression in large cohorts where no detailed clinical data is available and to other diseases, disability measures, and healthcare systems with minor modifications. In addition to expected factors, we identified novel informative factors like gynecological disorders, upper respiratory tract infections, bronchial asthma, and thyroiditis requiring further validation and investigation.

## Author Contributions

**Onur Dereli:** conceptualization, formal analysis, methodology, visualization, writing – original draft, writing – review and editing. **Jochen Behringer:** writing – review and editing, data curation, resources. **Achim Berthele:** writing – review and editing. **Alexander Hapfelmeier:** conceptualization, writing – review and editing, methodology. **Bernhard Hemmer:** writing – review and editing. **Christiane Gasperi:** conceptualization, funding acquisition, writing – original draft, writing – review and editing, supervision.

## Consent

There was no need for written informed consent from participants.

## Conflicts of Interest

Onur Dereli, Jochen Behringer, and Alexander Hapfelmeier report no disclosures relevant to the manuscript. Achim Berthele receives funding from the German Federal Ministry of Education and Research (BMBF; grant 01ZZ2102B). He has received consulting and/or speaker fees from Alexion, Biogen, Horizon, Novartis, Roche, and Sandoz/Hexal, and his institution has received compensation for clinical trials from Alexion, Biogen, Merck, Novartis, Roche, and Sanofi Genzyme; all outside the present work. Bernhard Hemmer has served on scientific advisory boards for Novartis; he has served as a DMSC member for AllergyCare, Sandoz, Polpharma, Biocon, and TG therapeutics; his institution received research grants from Roche for multiple sclerosis research. He has received honoraria for counseling (Gerson Lehrmann Group). He holds part of two patents: one for the detection of antibodies against KIR4.1 in a subpopulation of patients with multiple sclerosis and one for genetic determinants of neutralizing antibodies to interferon. All conflicts are not relevant to the topic of the study. He is associated with DIFUTURE (Data Integration for Future Medicine) [BMBF 01ZZ1804[A‐I]]. He received funding for the study from the European Union's Horizon 2020 Research and Innovation Program [grant MultipleMS, EU RIA 733161] and the Deutsche Forschungsgemeinschaft (DFG, German Research Foundation) under Germany's Excellence Strategy within the framework of the Munich Cluster for Systems Neurology [EXC 2145 SyNergy—ID 390857198]. Christiane Gasperi received funding from the BMBF, the Deutsche Forschungsgemeinschaft (DFG, German Research Foundation), the Hertie Foundation, and the Hans and Klementia Langmatz Stiftung.

## Supporting information


**Data S1.** Supporting Information.

## Data Availability

This study analyzed anonymous healthcare claims data held by AOK Bayern. Approval by an ethics standards committee on human experimentation was not needed according to the Guidelines and Recommendations for Good Practice of Secondary Data Analysis [44]. Data protection regulations prohibit the open distribution of the underlying data. Approval was obtained from the AOK's data protection officer.

## References

[1] C. Walton , R. King , L. Rechtman , et al., “Rising Prevalence of Multiple Sclerosis Worldwide: Insights From the Atlas of MS, Third Edition,” Multiple Sclerosis Journal 26, no. 14 (2020): 1816–1821, 10.1177/1352458520970841. 33174475 PMC7720355

[2] R. Dobson and G. Giovannoni , “Multiple Sclerosis–A Review,” European Journal of Neurology 26 (2019): 27–40. 30300457 10.1111/ene.13819

[3] A. Abdelhak , P. Benkert , S. Schaedelin , et al., “Neurofilament Light Chain Elevation and Disability Progression in Multiple Sclerosis,” JAMA Neurology 80 (2023): 1317–1325. 37930670 10.1001/jamaneurol.2023.3997 PMC10628837

[4] J. Åkesson , S. Hojjati , S. Hellberg , et al., “Proteomics Reveal Biomarkers for Diagnosis, Disease Activity and Long‐Term Disability Outcomes in Multiple Sclerosis,” Nature Communications 14 (2023): 6903. 10.1038/s41467-023-42682-9 PMC10616092 37903821

[5] E. De Brouwer , T. Becker , Y. Moreau , et al., “Longitudinal Machine Learning Modeling of MS Patient Trajectories Improves Predictions of Disability Progression (Vol 208, 106180, 2021),” Computer Methods and Programs in Biomedicine 213 (2022): 106479. 34146771 10.1016/j.cmpb.2021.106180

[6] K. L. Kreft , E. Uzochukwu , S. Loveless , et al., “Relevance of Multiple Sclerosis Severity Genotype in Predicting Disease Course: A Real‐World Cohort,” Annals of Neurology 95, no. 3 (2023): 459–470. 37974536 10.1002/ana.26831

[7] M. T. Law , A. L. Traboulsee , D. K. Li , et al., “Machine Learning in Secondary Progressive Multiple Sclerosis: An Improved Predictive Model for Short‐Term Disability Progression,” Multiple Sclerosis Journal–Experimental, Translational and Clinical 5 (2019): 2055217319885983. 31723436 10.1177/2055217319885983 PMC6836306

[8] J. Liu , E. Kelly , and B. Bielekova , “Current Status and Future Opportunities in Modeling Clinical Characteristics of Multiple Sclerosis,” Frontiers in Neurology 13 (2022): 884089. 35720098 10.3389/fneur.2022.884089 PMC9198703

[9] J. F. Kurtzke , “Rating Neurologic Impairment in Multiple‐Sclerosis—An Expanded Disability Status Scale (Edss),” Neurology 33 (1983): 1444–1452. 6685237 10.1212/wnl.33.11.1444

[10] S. Meyer‐Moock , Y. S. Feng , M. Maeurer , F. W. Dippel , and T. Kohlmann , “Systematic Literature Review and Validity Evaluation of the Expanded Disability Status Scale (EDSS) and the Multiple Sclerosis Functional Composite (MSFC) in Patients With Multiple Sclerosis,” BMC Neurology 14 (2014): 58. 24666846 10.1186/1471-2377-14-58 PMC3986942

[11] World Health Organization , The ICD‐10 Classification of Mental and Behavioural Disorders: Diagnostic Criteria for Research (World Health Organization, 1993).

[12] B. Hemmer , K. Gehring , A. Bayas et al., “Diagnose und Therapie der Multiplen Sklerose, Neuromyelitis‐Optica‐Spektrum‐Erkrankungen und MOG‐IgG‐assoziierten Erkrankungen, S2k‐Leitlinie,” in: Deutsche Gesellschaft für Neurologie, (Hrsg.) Leitlinien für Diagnostik und Therapie in der Neurologie (Arbeitsgemeinschaft der Wissenschaftlichen Medizinischen Fachgesellschaften Online, 2024), www.dgn.org/leitlinien.

[13] Eurostat , “NUTS Overview,”, https://ec.europa.eu/eurostat/web/nuts/overview (accessed 11.12.2024).

[14] Bundesamt für Justiz , “Social Code (SGB),”, https://www.gesetze‐im‐internet.de/sgb_11/ (accessed 07.10.2024).

[15] P. C. Consul , “A Simple Urn Model Dependent Upon Predetermined Strategy,” Sankhyā: The Indian Journal of Statistics, Series B 36 (1974): 391–399.

[16] P. C. Consul , “On Some Properties and Applications of Quasi‐Binomial Distribution,” Communications in Statistics ‐ Theory and Methods 19 (1990): 477–504.

[17] G. Ke , Q. Meng , T. Finley , et al., “Lightgbm: A Highly Efficient Gradient Boosting Decision Tree,” in Advances in Neural Information Processing Systems (Curran Associates Inc, 2017), 3149–3157.

[18] Y. Shi , G. Ke , D. Soukhavong , et al., “lightgbm: Light Gradient Boosting Machine,” 2022.

[19] F. Sigrist , “Latent Gaussian Model Boosting,” IEEE Transactions on Pattern Analysis and Machine Intelligence 45 (2023): 1894–1905. 35439126 10.1109/TPAMI.2022.3168152

[20] S. Lundberg , “A Unified Approach to Interpreting Model Predictions,” 2017. arXiv preprint arXiv:170507874.

[21] P. Emery , C. Solem , I. Majer , J. C. Cappelleri , and M. Tarallo , “A European Chart Review Study on Early Rheumatoid Arthritis Treatment Patterns, Clinical Outcomes, and Healthcare Utilization,” Rheumatology International 35 (2015): 1837–1849. 26164150 10.1007/s00296-015-3312-3

[22] G. Roda , S. Chien Ng , P. G. Kotze , et al., “Crohn's Disease,” Nature Reviews Disease Primers 6 (2020): 22. 10.1038/s41572-020-0156-2 32242028

[23] Y. P. Wen and Z. G. Yu , “Identifying Shared Genetic Loci and Common Risk Genes of Rheumatoid Arthritis Associated With Three Autoimmune Diseases Based on Large‐Scale Cross‐Trait Genome‐Wide Association Studies,” Frontiers in Immunology 14 (2023): 1160397. 37377963 10.3389/fimmu.2023.1160397 PMC10291128

[24] Y. Yang , H. Musco , S. Simpson‐Yap , et al., “Investigating the Shared Genetic Architecture Between Multiple Sclerosis and Inflammatory Bowel Diseases,” Nature Communications 12 (2021): 5641. 10.1038/s41467-021-25768-0 PMC8463615 34561436

[25] A. Gajofatto and M. D. Benedetti , “Treatment Strategies for Multiple Sclerosis: When to Start, When to Change, When to Stop?,” World Journal of Clinical Cases 3 (2015): 545–555. 26244148 10.12998/wjcc.v3.i7.545 PMC4517331

[26] S. Hempel , G. D. Graham , N. Fu , et al., “A Systematic Review of Modifiable Risk Factors in the Progression of Multiple Sclerosis,” Multiple Sclerosis Journal 23 (2017): 525–533. 28151053 10.1177/1352458517690270

[27] C. B. Malpas , A. Manouchehrinia , S. Sharmin , et al., “Early Clinical Markers of Aggressive Multiple Sclerosis,” Brain 143 (2020): 1400–1413. 32386427 10.1093/brain/awaa081

[28] P. A. McCombe , T. P. Gordon , and M. W. Jackson , “Bladder Dysfunction in Multiple Sclerosis,” Expert Review of Neurotherapeutics 9 (2009): 331–340. 19271942 10.1586/14737175.9.3.331

[29] M. M. Paz Soldan , M. Novotna , N. Abou Zeid , et al., “Relapses and Disability Accumulation in Progressive Multiple Sclerosis,” Neurology 84 (2015): 81–88. 25398229 10.1212/WNL.0000000000001094 PMC4336097

[30] H. L. Zwibel , “Contribution of Impaired Mobility and General Symptoms to the Burden of Multiple Sclerosis,” Advances in Therapy 26 (2009): 1043–1057. 20082242 10.1007/s12325-009-0082-x

[31] B. C. Healy , E. N. Ali , C. R. G. Guttmann , et al., “Smoking and Disease Progression in Multiple Sclerosis,” Archives of Neurology 66 (2009): 858–864. 19597087 10.1001/archneurol.2009.122 PMC2754172

[32] K. A. McKay , H. Tremlett , J. D. Fisk , et al., “Psychiatric Comorbidity Is Associated With Disability Progression in Multiple Sclerosis,” Neurology 90 (2018): e1316–e1323. 29523642 10.1212/WNL.0000000000005302 PMC5894930

[33] P. Tettey , S. Simpson, Jr. , B. V. Taylor , and I. A. van der Mei , “Vascular Comorbidities in the Onset and Progression of Multiple Sclerosis,” Journal of the Neurological Sciences 347 (2014): 23–33. 25454639 10.1016/j.jns.2014.10.020

[34] C. Gasperi , A. Hapfelmeier , A. Schneider , K. A. Kuhn , E. Donnachie , and B. Hemmer , “Association of Pregnancies With Risk of Multiple Sclerosis,” Multiple Sclerosis Journal 28 (2022): 1630–1640. 35301890 10.1177/13524585221080542 PMC9315178

[35] C. Gasperi , A. Hapfelmeier , T. Daltrozzo , A. Schneider , E. Donnachie , and B. Hemmer , “Systematic Assessment of Medical Diagnoses Preceding the First Diagnosis of Multiple Sclerosis,” Neurology 96 (2021): e2977–e2988. 33903190 10.1212/WNL.0000000000012074

[36] P. Fallahi , S. M. Ferrari , I. Ruffilli , et al., “The Association of Other Autoimmune Diseases in Patients With Autoimmune Thyroiditis: Review of the Literature and Report of a Large Series of Patients,” Autoimmunity Reviews 15 (2016): 1125–1128. 27639841 10.1016/j.autrev.2016.09.009

[37] E. Hill , H. Abboud , and F. B. S. Briggs , “Prevalence of Asthma in Multiple Sclerosis: A United States Population‐Based Study,” Multiple Sclerosis and Related Disorders 28 (2019): 69–74. 30557818 10.1016/j.msard.2018.12.012

[38] E. Ciampi , R. Uribe‐San‐Martin , B. Soler , et al., “Prevalence of Comorbidities in Multiple Sclerosis and Impact on Physical Disability According to Disease Phenotypes,” Multiple Sclerosis and Related Disorders 46 (2020): 102565. 33039942 10.1016/j.msard.2020.102565

[39] H. Ghoshouni , N. Rafiei , M. Yazdan Panah , et al., “Asthma and Chronic Obstructive Pulmonary Disease (COPD) in People With Multiple Sclerosis: A Systematic Review and Meta‐Analysis,” Multiple Sclerosis and Related Disorders 85 (2024): 105546. 38507873 10.1016/j.msard.2024.105546

[40] R. Bergamaschi , S. Villani , M. Crabbio , et al., “Inverse Relationship Between Multiple Sclerosis and Allergic Respiratory Diseases,” Neurological Sciences 30 (2009): 115–118. 19259620 10.1007/s10072-009-0036-8

[41] M. Zhang , Z. Ma , H. Qin , and Z. Yao , “Thyroid Hormone Potentially Benefits Multiple Sclerosis via Facilitating Remyelination,” Molecular Neurobiology 53 (2016): 4406–4416. 26243185 10.1007/s12035-015-9375-z

[42] F. Hoffmann and D. Koller , “Different Regions, Differently Insured Populations? Socio‐Demographic and Health‐Related Differences Between Insurance Funds,” Gesundheitswesen 79 (2017): e1–e9. 26492391 10.1055/s-0035-1564074

[43] J. Huber and A. Mielck , “Morbidity and Healthcare Differences Between Insured in the Statutory (“GKV”) and Private Health Insurance (“PKV”) in Germany. Review of Empirical Studies,” Bundesgesundheitsblatt, Gesundheitsforschung, Gesundheitsschutz 53 (2010): 925–938. 20853090 10.1007/s00103-010-1119-7

[44] E. Swart , H. Gothe , S. Geyer , et al., “Gute praxis sekundärdatenanalyse (GPS): leitlinien und empfehlungen,” Das Gesundheitswesen 77 (2015): 120–126. 25622207 10.1055/s-0034-1396815

