# Supplementary material for: Insights Into Disability and Disability Progression in People With Multiple Sclerosis Using Large‐Scale Healthcare Data
Source: Eur J Neurol. 2025 Mar 30;32(4):e70124. doi: 10.1111/ene.70124 (PMC11955418; doi:10.1111/ene.70124)
Supplement: Supplementary file 1 — Data S1. Supporting Information. [file ENE-32-e70124-s001.docx]

**Supporting Information**

**Insights into Disability and Disability Progression in People with Multiple Sclerosis Using Large-Scale Healthcare Data**

Onur Dereli^1^, Jochen Behringer^2^, Achim Berthele^1^, Alexander Hapfelmeier^3,4,5^, Bernhard Hemmer ^1,6^, Christiane Gasperi ^1^

1 Department of Neurology, TUM School of Medicine and Health, TUM University Hospital, Technical University of Munich, Ismaninger Str. 22, 81675 Munich, Germany

2 AOK Bayern, Munich, Germany

3 Institute of AI and Informatics in Medicine, TUM School of Medicine and Health, TUM University Hospital, Technical University of Munich, Ismaninger Str. 22, 81675 Munich, Germany.

4 Institute of General Practice and Health Services Research, TUM School of Medicine and Health, TUM University Hospital, Technical University of Munich, Orleansstr. 47, 81667, Munich, Germany.

5 Munich Center for Health Economics and Policy, Munich, Germany

6 Munich Cluster for Systems Neurology (SyNergy), Germany

Table of Contents

[Supplementary Methods 2](#_Toc190097506)

[Data Filtering and Cleaning 2](#_Toc190097507)

[Selecting the Model Configuration 2](#_Toc190097508)

[Learning Model 3](#_Toc190097509)

[Performance Metrics 3](#_Toc190097510)

[Supplementary Figures 4](#_Toc190097511)

[Figure S1: The overview of our algorithm proposed for disability progression prediction. 4](#_Toc190097512)

[Figure S2: The comparisons of MS, CD, RA, and CTR, with respect to utilizing each assistive device at least once, for different age groups 5](#_Toc190097513)

[Figure S3: The comparisons of MS, CD, RA, and CTR with respect to utilizing each assistive device at least once. 6](#_Toc190097514)

[Figure S4: The comparisons of MS, CD, RA, and CTR with respect to utilizing nursing care for different age groups. 7](#_Toc190097515)

[Figure S5: The comparisons of MS, CD, RA, and CTR with respect to each nursing care level. 8](#_Toc190097516)

[Figure S6: The comparisons of MS, CD, RA, and CTR, with respect to frequencies of utilizing a) physiotherapy, b) ergotherapy remedies for different age groups. 9](#_Toc190097517)

[Figure S7: Predictive performances of the MS progression prediction model that uses healthcare data as input. 10](#_Toc190097518)

[Supplementary Tables 11](#_Toc190097519)

[Table S1: List of remedies included in each remedy group 11](#_Toc190097520)

[Table S2: List of ICD-10 codes included in the prediction model 11](#_Toc190097521)

[Table S3: The list of disease-modifying therapies (DMTs) used in the prediction models and their category based on the effectiveness 21](#_Toc190097522)

[Table S4: Assessment criteria for determining the need for nursing care 22](#_Toc190097523)

[Table S5: Levels of nursing care assigned to the individuals 22](#_Toc190097524)

[Table S6: List of hyperparameters used for training the prediction model 22](#_Toc190097525)

[Table S7: The comparisons of MS, CD, RA, and CTR for each age group, with respect to all healthcare parameters included in the study. 23](#_Toc190097526)

[Table S8: The comparisons of MS, CD, RA, and CTR for each age group, with respect to all assistive devices included in the study. 25](#_Toc190097527)

[Table S9: The comparisons of MS, CD, RA, and CTR for each age group, with respect to each nursing care level. 27](#_Toc190097528)

[Table S10: Predictive performances obtained through 50 replications using the area under the receiver operating characteristic curve (AUROC), the area under the precision-recall curve (AUPR), F1, and Average Balanced Accuracy (Avg. Balanced Accr.) 29](#_Toc190097529)

[Table S11: The mean absolute Shapley values that show the contributions of each feature to the disease progression prediction. 30](#_Toc190097530)

[Table S12: The predictive performances obtained for each model configuration (varying observation durations and missing value percentages) based on the area under the precision-recall curve (AUPR) values over 50 replications. 42](#_Toc190097531)

# **Supplementary Methods**

## **Data Filtering and Cleaning**

We applied a thorough filtering and cleaning process to the data. Information about the reliability of the diagnoses was available only for outpatient records without hospital admissions, and we included only the ones with secured diagnoses in our study. We included outpatient records with hospital admissions and inpatient records without considering the diagnosis reliability since we did not have that information for such records. We included a Multiple Sclerosis (MS) diagnosis (i.e., G35) in the study if a neurologist made it. However, if the specialist information for a diagnosis was unavailable, we kept this diagnosis record in the analyses to have a sufficient number of individuals for the study. For the MS cohort, we excluded individuals diagnosed with any of the other two autoimmune diseases (i.e., CD or RA). We further excluded individuals with possible demyelinating diseases from the healthy control group since such diseases could imply an early stage of MS or related diseases. For this purpose, we discarded individuals with recorded ICD-10 codes related to encephalitis, myelitis, and encephalomyelitis, as well as inflammatory diseases of the CNS (G04, G05, G09), demyelinating diseases of the CNS (G36, G37), retrobulbar neuritis and other disorders of the optic nerve and the visual pathways (H46, H47, H48), and abnormal findings on diagnostic imaging of CNS (R90) from the control cohort.

We excluded individuals whose insurance status was unknown for 20% or more of the total insurance period and individuals with less than one year of total insurance period to obtain a more robust dataset.

We included individuals with Primary-Progressive MS (PPMS, G352), Relapsing-Remitting MS (RRMS, G351), and Secondary-Progressive MS (SPMS, G353). For individuals diagnosed with both PPMS and RRMS or PPMS and SPMS, we relied on the most recent diagnosis over the previous ones. In cases where a patient received different diagnoses in the last observation period, we selected the most common diagnosis within the last two years as the accurate one. If we could not identify the most common diagnosis in the last two years, we excluded the individual from our dataset.

After filtering and cleaning, the resulting dataset included 11961 individuals with MS, 21884 with CD, 105450 with RA, and 82667 controls

## **Selecting the Model Configuration**

To reduce the missing information rate and include a sufficient number of individuals in the study, we evaluated models with different durations for the patient trajectory and different levels of missing information. We considered two, three, and four years of observation durations with quarterly time bins for recording periods. We created subsets for each configuration, including individuals with no missing value and individuals with missing information at most 10 and 30% of the recording periods. Since there were slight or no significant differences between different model configurations in terms of predictive performances, we selected a 3-year patient trajectory and missing information up to 30% of the whole observation period for training our prediction model. Table S12 shows the predictive performances obtained for each model configuration on the validation set over 50 replications.

## **Learning Model**

### Performance Metrics

We used the area under the receiver operating characteristic curve (AUROC) and the area under the precision-recall curve (AUPR) as performance metrics to evaluate the predictive performance of our approach. AUROC and AUPR are well-known binary classification metrics that measure the ability of models to distinguish between classes. Larger AUROC and AUPR values correspond to better predictive performance. The baseline value for AUROC is 0.5, meaning that the model cannot distinguish classes better than random guessing (i.e., random classifier). For the AUPR, the baseline value corresponds to the fraction of positive classes in the data. In our case, it is 0.082 ((number of positive samples) / (number of total samples)). AUPR is a useful metric for evaluating the performance of imbalanced data. We also reported two other performance metrics, Average Balanced Accuracy and F1, used to assess the predictive performances of imbalanced data. We picked the classification threshold for F1 and Average Balanced Accuracy using the geometric mean of sensitivity and specificity values.

To interpret the contribution of healthcare factors on disease progress prediction, we utilized the SHapley Additive exPlanations (SHAP) method[1]. Shapley values are derived by evaluating all possible combinations of feature subsets, allowing us to interpret the machine learning models by quantifying the contribution of each feature to a specific prediction task. Features with higher mean absolute Shapley values, representing the average magnitude of a feature’s effect, have a greater contribution to the prediction task. The degree of contribution of the model features to the prediction task is illustrated in a beeswarm plot. In this plot, the yellow-magenta gradient scale indicates the original value of features, i.e., yellow represents the lowest, and magenta represents the highest feature values (see Table 2 for the corresponding values of the numerical and categorical features). Features with positive Shapley values have an increasing impact on disability progression, while those with negative values have the opposite effect.

# **Supplementary Figures**

### Figure S1: The overview of our algorithm proposed for disability progression prediction.

**Figure S1:** The overview of our algorithm proposed for disability progression prediction. Our algorithm takes healthcare parameters (X), as well as a vector of binary indicators for disability progression (Y) defined using nursing care information as input. Those parameters are used to learn a function (f) to predict whether the individuals with MS will switch from nursing care level two or below to nursing care level three or above two years after their last observation.

### Figure S2: The comparisons of MS, CD, RA, and CTR, with respect to utilizing each assistive device at least once, for different age groups

**Figure S2:** The comparisons of MS, CD, RA, and CTR, with respect to utilizing each assistive device at least once, for different age groups. The odds ratios, their corresponding 95% confidence intervals, and adjusted p-values were obtained using GLM. MS was used as the base phenotype for all models, meaning that higher values for (1 / odds ratios) indicate a higher likelihood of observing the related outcome in MS. Abbreviations: Multiple Sclerosis (MS), Crohn’s Disease (CD), Rheumatoid Arthritis (RA), Controls (CTR), Adjusted (Adj.).

### Figure S3: The comparisons of MS, CD, RA, and CTR with respect to utilizing each assistive device at least once.

**Figure S3:** The comparisons of MS, CD, RA, and CTR with respect to utilizing each assistive device at least once. The odds ratios, their corresponding 95% confidence intervals, and adjusted p-values were obtained using GLM. MS was used as the base phenotype for all models, meaning that higher values for (1 / odds ratios) indicate a higher likelihood of observing the related outcome in MS. Abbreviations: Multiple Sclerosis (MS), Crohn’s Disease (CD), Rheumatoid Arthritis (RA), Controls (CTR), Adjusted (Adj.).

Figure S4: The comparisons of MS, CD, RA, and CTR with respect to utilizing nursing care for different age groups.

Figure S4: The comparisons of MS, CD, RA, and CTR with respect to utilizing nursing care for different age groups. The odds ratios, their corresponding 95% confidence intervals, and adjusted p-values were obtained using GLM. MS was used as the base phenotype for all models, meaning that higher values for (1 / odds ratios) indicate a higher likelihood of observing the related outcome in MS. Abbreviations: Multiple Sclerosis (MS), Crohn’s Disease (CD), Rheumatoid Arthritis (RA), Controls (CTR), Adjusted (Adj.).

Figure S5: The comparisons of MS, CD, RA, and CTR with respect to each nursing care level.

Figure S5: The comparisons of MS, CD, RA, and CTR with respect to each nursing care level. The odds ratios, their corresponding 95% confidence intervals, and adjusted p-values were obtained using multinomial logistic regression model. MS was used as the base phenotype for all models, meaning that higher values for (1 / odds ratios) indicate a higher likelihood of observing the related outcome in MS. Abbreviations: Multiple Sclerosis (MS), Crohn’s Disease (CD), Rheumatoid Arthritis (RA), Controls (CTR), Adjusted (Adj.).

### Figure S6: The comparisons of MS, CD, RA, and CTR, with respect to frequencies of utilizing a) physiotherapy, b) ergotherapy remedies for different age groups.

###

Figure S6: The comparisons of MS, CD, RA, and CTR, with respect to frequencies of utilizing A) physiotherapy, B) ergotherapy remedies for different age groups. The odds ratios, their corresponding 95% confidence intervals, and adjusted p-values were obtained using GLMs. MS was used as the base phenotype for all models, meaning that higher values for (1 / odds ratios) indicate a higher likelihood of observing the related outcome in MS. Abbreviations: Multiple Sclerosis (MS), Crohn’s Disease (CD), Rheumatoid Arthritis (RA), Controls (CTR), Adjusted (Adj.).

### **Figure S7: Predictive performances of the MS progression prediction model that uses healthcare data as input.**

Figure S7: Predictive performances of the MS progression prediction model that uses healthcare data as input. The violin plots show the area under the receiver operating characteristic curve (AUROC,) area under the precision-recall curve (AUPR), F1, and Average Balanced Accuracy values obtained by the model over 50 replications. The baseline AUROC and AUPR values of the random classifier are 0.5 and 0.082, respectively. Confidence intervals for each measure over 50 replications are given under each corresponding violin plot. Abbreviations: independent and identically distributed (IID), area under the receiver operating characteristic curve (AUROC), the area under the precision-recall curve (AUPR), confidence interval (CI).

# **Supplementary Tables**

### Table S1: List of remedies included in each remedy group

| **Definition** | **Remedy Group** |
| --- | --- |
| Massages | Physiotherapy |
| Man. Lymphatic drainage | Physiotherapy |
| Movement therapy/exercise treatment, EB | Physiotherapy |
| Movement therapy/Ü-_x000D_ exercise treatment GB | Physiotherapy |
| Physiotherapy, normal, EB | Physiotherapy |
| Physiotherapy, normal GB | Physiotherapy |
| Physiotherapy, special, EB | Physiotherapy |
| Physiotherapy, special, GB | Physiotherapy |
| Physiotherapy in the exercise pool, EB | Physiotherapy |
| Physiotherapy in the_x000D_ exercise pool, GB | Physiotherapy |
| Traction treatment/extension treatment | Physiotherapy |
| Manual therapy | Physiotherapy |
| Electrotherapy | Physiotherapy |
| Light therapy | Physiotherapy |
| Heat and cold therapy | Physiotherapy |
| Hydrotherapy | Physiotherapy |
| Medical baths | Physiotherapy |
| Inhalation therapy | Physiotherapy |
| Standardized combination of remedies | Physiotherapy |
| Other spa-specific forms of treatment | Physiotherapy |
| Additional positions for bath additives | Physiotherapy |
| Assessment of findings | Ergotherapy |
| Individual treatment | Ergotherapy |
| Group treatment | Ergotherapy |
| Thermal application, heat/cold | Ergotherapy |
| Rails | Ergotherapy |

### Table S2: List of ICD-10 codes included in the prediction model

| **ICD-10 Code/Definition** | **ICD-10 Code/Definition** |
| --- | --- |

| M40 | Kyphosis and lordosis |
| --- | --- |
| H49 | Paralytic strabismus |
| J00 | Acute nasopharyngitis [common cold] |
| R10 | Abdominal and pelvic pain |
| F40 | Phobic anxiety disorders |
| M95 | Other acquired deformities of musculoskeletal system and connective tissue |
| M60 | Myositis |
| N80 | Endometriosis |
| T08 | Fracture of the spine, level unspecified |
| M00 | Pyogenic arthritis |
| I10 | Essential (primary) hypertension |
| D10 | Benign neoplasm of mouth and pharynx |
| A00 | Cholera |
| R40 | Somnolence, stupor and coma |
| J20 | Acute bronchitis |
| L20 | Atopic dermatitis |
| E70 | Disorders of aromatic amino-acid metabolism |
| K20 | Esophagitis |
| R50 | Fever of other and unknown origin |
| J40 | Bronchitis, not specified as acute or chronic |
| J30 | Vasomotor and allergic rhinitis |
| H10 | Conjunctivitis |
| K55 | Vascular disorders of intestine |
| Q65 | Congenital deformities of hip |
| K50 | Crohn's disease [regional enteritis] |
| N70 | Salpingitis and oophoritis |
| E65 | Localized adiposity |
| R00 | Abnormalities of heart beat |
| E50 | Vitamin A deficiency |
| H60 | Otitis externa |
| T66 | Radiation sickness, unspecified |
| F10 | Alcohol related disorders |
| E00 | Congenital iodine-deficiency syndrome |
| B35 | Dermatophytosis |
| I80 | Phlebitis and thrombophlebitis |
| B99 | Other and unspecified infectious diseases |
| K70 | Alcoholic liver disease |
| N60 | Benign mammary dysplasia |
| B25 | Cytomegaloviral disease |
| F00 | Dementia in Alzheimer's disease |
| H00 | Hordeolum and chalazion |
| H90 | Conductive and sensorineural hearing loss |
| T80 | Complications following infusion, transfusion and therapeutic injection |
| S90 | Superficial injury of ankle, foot and toes |
| I95 | Hypotension |
| D50 | Iron deficiency anemia |
| H30 | Chorioretinal inflammation |
| B00 | Herpesviral [herpes simplex] infections |
| H25 | Age-related cataract |
| T79 | Certain early complications of trauma |
| J95 | Intraoperative and postprocedural complications and disorders of respiratory system |
| L60 | Nail disorders |
| I30 | Acute pericarditis |
| E10 | Type 1 diabetes mellitus |
| D37 | Neoplasm of uncertain behavior of oral cavity and digestive organs |
| M80 | Osteoporosis with current pathological fracture |
| D60 | Acquired pure red cell aplasia [erythroblastopenia] |
| H65 | Nonsuppurative otitis media |
| J09 | Influenza due to certain identified influenza viruses |
| K80 | Cholelithiasis |
| L80 | Vitiligo |
| S80 | Superficial injury of knee and lower leg |
| S00 | Superficial injury of head |
| S20 | Superficial injury of thorax |
| F50 | Eating disorders |
| H40 | Glaucoma |
| S60 | Superficial injury of wrist, hand and fingers |
| K40 | Inguinal hernia |
| N40 | Benign prostatic hyperplasia |
| H43 | Disorders of vitreous body |
| K90 | Intestinal malabsorption |
| R70 | Elevated erythrocyte sedimentation rate and abnormality of plasma viscosity |
| H55 | Nystagmus and other irregular eye movements |
| N17 | Acute kidney failure |
| I70 | Atherosclerosis |
| E20 | Hypoparathyroidism |
| L50 | Urticaria |
| S30 | Superficial injury of abdomen, lower back, pelvis and external genitals |
| S10 | Superficial injury of neck |
| M30 | Polyarteritis nodosa and related conditions |
| O20 | Hemorrhage in early pregnancy |
| I20 | Angina pectoris |
| D70 | Neutropenia |
| N25 | Disorders resulting from impaired renal tubular function |
| R83 | Abnormal findings in cerebrospinal fluid |
| L40 | Psoriasis |
| H15 | Disorders of sclera |
| F60 | Specific personality disorders |
| T00 | Superficial injuries involving several body regions |
| D65 | Disseminated intravascular coagulation [defibrination syndrome] |
| N10 | Acute pyelonephritis |
| B95 | Streptococcus, Staphylococcus, and Enterococcus as the cause of diseases classified elsewhere |
| N20 | Calculus of kidney and ureter |
| K00 | Disorders of tooth development and eruption |
| S40 | Superficial injury of shoulder and upper arm |
| R80 | Proteinuria |
| O80 | Encounter for full-term uncomplicated delivery |
| C00 | Malignant neoplasm of lip |
| S50 | Superficial injury of elbow and forearm |
| T51 | Toxic effect of alcohol |
| S70 | Superficial injury of hip and thigh |
| T20 | Burn and corrosion of head, face, and neck |
| L55 | Sunburn |
| O30 | Multiple gestation |
| F99 | Mental disorder, not otherwise specified |
| O85 | Puerperal sepsis |
| T15 | Foreign body on external eye |
| O60 | Preterm labor |
| K65 | Peritonitis |
| Q80 | Congenital ichthyosis |
| Q60 | Renal agenesis and other reduction defects of kidney |
| J90 | Pleural effusion |
| N00 | Acute nephritic syndrome |
| F20 | Schizophrenia |
| I05 | Rheumatic mitral valve diseases |
| I26 | Pulmonary embolism |
| D80 | Immunodeficiency with predominantly antibody defects |
| O10 | Pre-existing hypertension complicating pregnancy, childbirth and the puerperium |
| O00 | Ectopic pregnancy |
| D00 | Carcinoma in situ of oral cavity, esophagus and stomach |
| K35 | Acute appendicitis |
| F90 | Attention-deficit hyperactivity disorders |
| N99 | Intraoperative and postprocedural complications and disorders of genitourinary system |
| Q10 | Congenital malformations of eyelid, lacrimal apparatus and orbit |
| B85 | Pediculosis and phthiriasis |
| M41 | Scoliosis |
| H50 | Other strabismus |
| J01 | Acute sinusitis |
| R11 | Nausea and vomiting |
| F41 | Other anxiety disorders |
| M96 | Intraoperative and postprocedural complications and disorders of musculoskeletal system |
| M61 | Calcification and ossification of muscle |
| N81 | Female genital prolapse |
| T09 | Other injuries of the spine and trunk, level unspecified |
| M01 | Direct infections of joint in infectious and parasitic diseases classified elsewhere |
| I11 | Hypertensive heart disease |
| D11 | Benign neoplasm of major salivary glands |
| A01 | Typhoid and paratyphoid fevers |
| R41 | Other symp.* involving cognitive functions and awareness |
| J21 | Acute bronchiolitis |
| L21 | Seborrheic dermatitis |
| E71 | Disorders of branched-chain amino-acid metabolism and fatty-acid metabolism |
| K21 | Gastro-esophageal reflux disease |
| R51 | Headache |
| J41 | Simple and mucopurulent chronic bronchitis |
| J31 | Chronic rhinitis, nasopharyngitis and pharyngitis |
| H11 | Other disorders of conjunctiva |
| K56 | Paralytic ileus and intestinal obstruction without hernia |
| Q66 | Congenital deformities of feet |
| K51 | Ulcerative colitis |
| N71 | Inflammatory disease of uterus, except cervix |
| E66 | Overweight and obesity |
| R01 | Cardiac murmurs and other cardiac sounds |
| E51 | Thiamine deficiency |
| H61 | Other disorders of external ear |
| T67 | Effects of heat and light |
| F11 | Opioid related disorders |
| E01 | Iodine-deficiency related thyroid disorders and allied conditions |
| B36 | Other superficial mycoses |
| I81 | Portal vein thrombosis |
| K71 | Toxic liver disease |
| N61 | Inflammatory disorders of breast |
| B26 | Mumps |
| F01 | Vascular dementia |
| H01 | Other inflammation of eyelid |
| H91 | Other and unspecified hearing loss |
| T81 | Complications of procedures |
| S91 | Open wound of ankle, foot and toes |
| D51 | Vitamin B12 deficiency anemia |
| H31 | Other disorders of choroid |
| B01 | Varicella [chickenpox] |
| L01 | Impetigo |
| H26 | Other cataract |
| J96 | Respiratory failure |
| I31 | Other diseases of pericardium |
| E11 | Type 2 diabetes mellitus |
| D38 | Neoplasm of uncertain behavior of middle ear and respiratory and intrathoracic organs |
| M81 | Osteoporosis without current pathological fracture |
| D61 | Other aplastic anemias and other bone marrow failure syndromes |
| H66 | Suppurative and unspecified otitis media |
| J10 | Influenza due to other identified influenza virus |
| K81 | Cholecystitis |
| L81 | Other disorders of pigmentation |
| S81 | Open wound of knee and lower leg |
| S01 | Open wound of head |
| S21 | Open wound of thorax |
| F51 | Sleep disorders not due to a substance or known physiological condition |
| S61 | Open wound of wrist, hand and fingers |
| K41 | Femoral hernia |
| N41 | Inflammatory diseases of prostate |
| H44 | Disorders of globe |
| K91 | Intraoperative and postprocedural complications and disorders of digestive system |
| R71 | Abnormality of red blood cells |
| A31 | Infection due to other mycobacteria |
| N18 | Chronic kidney disease (CKD) |
| I71 | Aortic aneurysm and dissection |
| E21 | Hyperparathyroidism and other disorders of parathyroid gland |
| L51 | Erythema multiforme |
| S31 | Open wound of abdomen, lower back, pelvis and external genitals |
| S11 | Open wound of neck |
| M31 | Other necrotizing vasculopathies |
| O21 | Excessive vomiting in pregnancy |
| I21 | Acute myocardial infarction |
| N26 | Unspecified contracted kidney |
| R84 | Abnormal findings in specimens from respiratory organs and thorax |
| L41 | Parapsoriasis |
| H16 | Keratitis |
| F61 | Combined and other personality disorders |
| T01 | Open wounds involving multiple body regions |
| D66 | Hereditary factor VIII deficiency |
| N11 | Chronic tubulo-interstitial nephritis |
| B96 | Other bacterial agents as the cause of diseases classified elsewhere |
| N21 | Calculus of lower urinary tract |
| K01 | Embedded and impacted teeth |
| S41 | Open wound of shoulder and upper arm |
| R81 | Glycosuria |
| O81 | Use a seal for sealing or vacuum extraction |
| S51 | Open wound of elbow and forearm |
| S71 | Open wound of hip and thigh |
| T21 | Burn and corrosion of trunk |
| L56 | Other acute skin changes due to ultraviolet radiation |
| O86 | Other puerperal infections |
| T16 | Foreign body in ear |
| O61 | Failed induction of labor |
| K66 | Other disorders of peritoneum |
| Q81 | Epidermolysis bullosa |
| Q61 | Cystic kidney disease |
| J61 | Pneumoconiosis due to asbestos and other mineral fibers |
| F21 | Schizotypal disorder |
| I06 | Rheumatic aortic valve diseases |
| I27 | Other pulmonary heart diseases |
| D81 | Combined immunodeficiencies |
| O11 | Pre-existing hypertension with pre-eclampsia |
| O01 | Hydatidiform mole |
| A51 | Early syphilis |
| E16 | Other disorders of pancreatic internal secretion |
| D01 | Carcinoma in situ of other and unspecified digestive organs |
| K36 | Other appendicitis |
| F91 | Conduct disorders |
| E41 | Nutritional marasmus |
| Q11 | Anophthalmos, microphthalmos and macrophthalmos |
| B86 | Scabies |
| M42 | Spinal osteochondrosis |
| H51 | Other disorders of binocular movement |
| J02 | Acute pharyngitis |
| R12 | Heartburn |
| F42 | Obsessive-compulsive disorder |
| M62 | Other disorders of muscle |
| T10 | Fracture of the upper extremity, level unspecified |
| M02 | Postinfective and reactive arthropathies |
| I12 | Hypertensive chronic kidney disease |
| D12 | Benign neoplasm of colon, rectum, anus and anal canal |
| A02 | Other salmonella infections |
| R42 | Dizziness and giddiness |
| J22 | Unspecified acute lower respiratory infection |
| L22 | Diaper dermatitis |
| E72 | Other disorders of amino-acid metabolism |
| K22 | Other diseases of esophagus |
| R52 | Pain, unspecified |
| J42 | Unspecified chronic bronchitis |
| J32 | Chronic sinusitis |
| K57 | Diverticular disease of intestine |
| Q67 | Congenital musculoskeletal deformities of head, face, spine and chest |
| K52 | Other and unspecified noninfective gastroenteritis and colitis |
| N72 | Inflammatory disease of cervix uteri |
| E67 | Other hyperalimentation |
| R02 | Gangrene, not elsewhere classified |
| H62 | Disorders of external ear in diseases classified elsewhere |
| T68 | Hypothermia |
| F12 | Cannabis related disorders |
| E02 | Subclinical iodine-deficiency hypothyroidism |
| B37 | Candidiasis |
| I82 | Other venous embolism and thrombosis |
| K72 | Hepatic failure |
| N62 | Hypertrophy of breast |
| B27 | Infectious mononucleosis |
| F02 | Dementia in other diseases classified elsewhere |
| H02 | Other disorders of eyelid |
| H92 | Otalgia and effusion of ear |
| T82 | Complications of cardiac and vascular prosthetic devices, implants and grafts |
| S92 | Fracture of foot and toe, except ankle |
| I97 | Intraoperative and postprocedural complications and disorders of circulatory system |
| D52 | Folate deficiency anemia |
| H32 | Chorioretinal disorders in diseases classified elsewhere |
| B02 | Zoster [herpes zoster] |
| L02 | Cutaneous abscess, furuncle and carbuncle |
| H27 | Other disorders of lens |
| E12 | Diabetes mellitus associated with malnutrition |
| D39 | Neoplasm of uncertain behavior of female genital organs |
| M82 | Osteoporosis in diseases classified elsewhere |
| D62 | Acute posthemorrhagic anemia |
| H67 | Otitis media in diseases classified elsewhere |
| J11 | Influenza due to unidentified influenza virus |
| K82 | Other diseases of gallbladder |
| L82 | Seborrheic keratosis |
| S82 | Fracture of lower leg, including ankle |
| S02 | Fracture of skull and facial bones |
| S22 | Fracture of rib(s), sternum and thoracic spine |
| F52 | Sexual dysfunction not due to a substance or known physiological condition |
| H42 | Glaucoma in diseases classified elsewhere |
| S62 | Fracture at wrist and hand level |
| K42 | Umbilical hernia |
| N42 | Other and unspecified disorders of prostate |
| K92 | Other diseases of digestive system |
| R72 | Leukocyte alteration, not elsewhere classified |
| A32 | Listeriosis |
| H57 | Other disorders of eye and adnexa |
| N19 | Unspecified kidney failure |
| I72 | Other aneurysm |
| E22 | Hyperfunction of pituitary gland |
| L52 | Erythema nodosum |
| S32 | Fracture of lumbar spine and pelvis |
| S12 | Fracture of cervical vertebra and other parts of neck |
| M32 | Systemic lupus erythematosus (SLE) |
| O22 | Venous complications and hemorrhoids in pregnancy |
| I22 | Subsequent ST elevation (STEMI) and non-ST elevation (NSTEMI) myocardial infarction |
| D72 | Other disorders of white blood cells |
| N27 | Small kidney of unknown cause |
| R85 | Abnormal findings in specimens from digestive organs and abdominal cavity |
| L42 | Pityriasis rosea |
| H17 | Corneal scars and opacities |
| F62 | Persistent personality changes not due to brain damage or disease |
| T02 | Fractures involving multiple body regions |
| D67 | Hereditary factor IX deficiency |
| N12 | Tubulo-interstitial nephritis, not specified as acute or chronic |
| B97 | Viral agents as the cause of diseases classified elsewhere |
| K02 | Dental caries |
| S42 | Fracture of shoulder and upper arm |
| R82 | Other and unspecified abnormal findings in urine |
| O82 | Encounter for cesarean delivery without indication |
| S52 | Fracture of forearm |
| S72 | Fracture of femur |
| T22 | Burn and corrosion of shoulder and upper limb, except wrist and hand |
| L57 | Skin changes due to chronic exposure to nonionizing radiation |
| O32 | Maternal care for malpresentation of fetus |
| O87 | Venous complications and hemorrhoids in the puerperium |
| T17 | Foreign body in respiratory tract |
| O62 | Abnormalities of forces of labor |
| Q82 | Other congenital malformations of skin |
| Q62 | Congenital obstructive defects of renal pelvis and congenital malformations of ureter |
| N02 | Recurrent and persistent hematuria |
| F22 | Delusional disorders |
| I07 | Rheumatic tricuspid valve diseases |
| I28 | Other diseases of pulmonary vessels |
| D82 | Immunodeficiency associated with other major defects |
| O12 | Gestational [pregnancy-induced] edema and proteinuria without hypertension |
| O02 | Other abnormal products of conception |
| A52 | Late syphilis |
| D02 | Carcinoma in situ of middle ear and respiratory system |
| K37 | Unspecified appendicitis |
| F92 | Combined disorder of social behavior and emotions |
| Q12 | Congenital lens malformations |
| B87 | Myiasis |
| M43 | Other deforming dorsopathies |
| H52 | Disorders of refraction and accommodation |
| J03 | Acute tonsillitis |
| R13 | Aphagia and dysphagia |
| F43 | Reaction to severe stress, and adjustment disorders |
| M63 | Disorders of muscle in diseases classified elsewhere |
| N83 | Noninf. dis.* of ovary, fallopian tube and broad ligament |
| T11 | Other injuries of upper extremity, level unspecified |
| I13 | Hypertensive heart and chronic kidney disease |
| D13 | Benign neoplasm of other and ill-defined parts of digestive system |
| R43 | Disturbances of smell and taste |
| L23 | Allergic contact dermatitis |
| E73 | Lactose intolerance |
| R53 | Malaise and fatigue |
| J43 | Emphysema |
| J33 | Nasal polyp |
| H13 | Conditions of the conjunctiva in diseases classified elsewhere |
| K58 | Irritable bowel syndrome |
| Q68 | Other congenital musculoskeletal deformities |
| N73 | Other female pelvic inflammatory diseases |
| E68 | Sequelae of hyperalimentation |
| R03 | Abnormal blood-pressure reading, without diagnosis |
| E53 | Deficiency of other B group vitamins |
| T69 | Other effects of reduced temperature |
| F13 | Sedative, hypnotic, or anxiolytic related disorders |
| E03 | Other hypothyroidism |
| I83 | Varicose veins of lower extremities |
| K73 | Chronic hepatitis |
| N63 | Unspecified lump in breast |
| F03 | Unspecified dementia |
| H03 | Affections of the eyelid in diseases classified elsewhere |
| H93 | Other disorders of ear |
| T83 | Complications of genitourinary prosthetic devices, implants and grafts |
| S93 | Dislocation and sprain of joints and ligaments at ankle, foot and toe level |
| I98 | Other disorders of the circulatory system in diseases classified elsewhere |
| D53 | Other nutritional anemias |
| H33 | Retinal detachments and breaks |
| L03 | Cellulitis and acute lymphangitis |
| H28 | Cataract in diseases classified elsewhere |
| J98 | Other respiratory disorders |
| L63 | Alopecia areata |
| I33 | Acute and subacute endocarditis |
| E13 | Other specified diabetes mellitus |
| D40 | Neoplasm of uncertain behavior of male genital organs |
| M83 | Adult osteomalacia |
| D63 | Anemia in chronic diseases classified elsewhere |
| H68 | Eustachian salpingitis and obstruction |
| J12 | Viral pneumonia |
| K83 | Other diseases of biliary tract |
| L83 | Acanthosis nigricans |
| S83 | Dislocation and sprain of joints and ligaments of knee |
| S03 | Dislocation and sprain of joints and ligaments of head |
| S23 | Dislocation and sprain of joints and ligaments of thorax |
| F53 | Mental and behavioral disorders associated with the puerperium |
| S63 | Dislocation and sprain of joints and ligaments at wrist and hand level |
| K43 | Ventral hernia |
| N43 | Hydrocele and spermatocele |
| K93 | Diseases of other digestive organs in diseases classified elsewhere |
| R73 | Elevated blood glucose level |
| H58 | Other disorders of eye and adnexa in diseases classified elsewhere |
| I73 | Other peripheral vascular diseases |
| E23 | Hypofunction and other disorders of the pituitary gland |
| L53 | Other erythematous conditions |
| S33 | Dislocation and sprain of joints and ligaments of lumbar spine and pelvis |
| S13 | Dislocation and sprain of joints and ligaments at neck level |
| M33 | Dermatopolymyositis |
| O23 | Infections of genitourinary tract in pregnancy |
| D73 | Diseases of spleen |
| N28 | Other disorders of kidney and ureter |
| R86 | Abnormal findings in specimens from male genital organs |
| L43 | Lichen planus |
| H18 | Other disorders of cornea |
| F63 | Impulse disorders |
| T03 | Dislocations, sprains and strains involving multiple body regions |
| D68 | Other coagulation defects |
| N13 | Obstructive and reflux uropathy |
| B98 | Other specified infectious agents causing diseases classified in other chapters |
| N23 | Unspecified renal colic |
| K03 | Other diseases of hard tissues of teeth |
| S43 | Dislocation and sprain of joints and ligaments of shoulder girdle |
| S53 | Dislocation and sprain of joints and ligaments of elbow |
| A68 | Relapsing fevers |
| S73 | Dislocation and sprain of joint and ligaments of hip |
| T23 | Burn and corrosion of wrist and hand |
| L58 | Radiodermatitis |
| O33 | Maternal care for disproportion |
| O88 | Obstetric embolism |
| T18 | Foreign body in alimentary tract |
| O63 | Long labor |
| Q83 | Congenital malformations of breast |
| Q63 | Other congenital malformations of kidney |
| J93 | Pneumothorax and air leak |
| N03 | Chronic nephritic syndrome |
| F23 | Brief psychotic disorder |
| I08 | Multiple valve diseases |
| D83 | Common variable immunodeficiency |
| O13 | Gestational [pregnancy-induced] hypertension without significant proteinuria |
| O03 | Spontaneous abortion |
| A53 | Other and unspecified syphilis |
| D03 | Melanoma in situ |
| K38 | Other diseases of appendix |
| F93 | Emotional disorders with onset specific to childhood |
| A23 | Brucellosis |
| E43 | Unspecified severe protein-calorie malnutrition |
| Q13 | Congenital malformations of anterior segment of eye |
| B88 | Other infestations |
| J04 | Acute laryngitis and tracheitis |
| R14 | Flatulence and related conditions |
| F44 | Dissociative and conversion disorders |
| M99 | Biomechanical lesions |
| N84 | Polyp of female genital tract |
| T12 | Fracture of lower extremity, level unspecified |
| D14 | Benign neoplasm of middle ear and respiratory system |
| A04 | Other bacterial intestinal infections |
| R44 | Other symp.* involving general sensations and perceptions |
| L24 | Irritant contact dermatitis |
| E74 | Other disorders of carbohydrate metabolism |
| R54 | Age-related physical debility |
| J44 | Other chronic obstructive pulmonary disease |
| J34 | Other and unspecified disorders of nose and nasal sinuses |
| K59 | Other functional intestinal disorders |
| N74 | Female pelvic inflammatory disorders in diseases classified elsewhere |
| R04 | Hemorrhage from respiratory passages |
| E54 | Ascorbic acid deficiency |
| T70 | Effects of air pressure and water pressure |
| F14 | Cocaine related disorders |
| E04 | Other nontoxic goiter |
| I84 | Haemorrhoids |
| K74 | Fibrosis and cirrhosis of liver |
| N64 | Other disorders of breast |
| F04 | Amnestic disorder due to known physiological condition |
| H04 | Disorders of lacrimal system |
| H94 | Other disorders of ear in diseases classified elsewhere |
| T84 | Complications of internal orthopedic prosthetic devices, implants and grafts |
| S94 | Injury of nerves at ankle and foot level |
| I99 | Other and unspecified disorders of circulatory system |
| H34 | Retinal vascular occlusions |
| L04 | Acute lymphadenitis |
| J99 | Respiratory disorders in diseases classified elsewhere |
| L64 | Androgenic alopecia |
| I34 | Nonrheumatic mitral valve disorders |
| E14 | Unspecified diabetes mellitus |
| D41 | Neoplasm of uncertain behavior of urinary organs |
| M84 | Disorder of continuity of bone |
| D64 | Other anemias |
| H69 | Other and unspecified disorders of Eustachian tube |
| J13 | Pneumonia due to Streptococcus pneumoniae |
| L84 | Corns and callosities |
| S84 | Injury of nerves at lower leg level |
| S04 | Injury of cranial nerve |
| S24 | Injury of nerves and spinal cord at thorax level |
| F54 | Psychological and behavioral factors associated with disorders or diseases classified elsewhere |
| S64 | Injury of nerves at wrist and hand level |
| K44 | Diaphragmatic hernia |
| N44 | Noninf. dis.* of testis |
| R74 | Abnormal serum enzyme levels |
| H59 | Intraoperative and postprocedural complications and disorders of eye and adnexa |
| I74 | Arterial embolism and thrombosis |
| E24 | Cushing's syndrome |
| O98 | Maternal infectious and parasitic diseases classifiable elsewhere but complicating pregnancy, childbirth and the puerperium |
| S34 | Injury of lumbar and sacral spinal cord and nerves at abdomen, lower back and pelvis level |
| S14 | Injury of nerves and spinal cord at neck level |
| M34 | Systemic sclerosis [scleroderma] |
| O24 | Diabetes mellitus in pregnancy, childbirth, and the puerperium |
| I24 | Other acute ischemic heart diseases |
| N29 | Other disorders of kidney and ureter in diseases classified elsewhere |
| R87 | Abnormal findings in specimens from female genital organs |
| L44 | Other papulosquamous disorders |
| H19 | Affections of the sclera and cornea in diseases classified elsewhere |
| F64 | Gender identity disorders |
| T04 | Crushing injuries involving multiple body regions |
| D69 | Purpura and other hemorrhagic conditions |
| K04 | Diseases of pulp and periapical tissues |
| S44 | Injury of nerves at shoulder and upper arm level |
| S54 | Injury of nerves at forearm level |
| A69 | Other spirochetal infections |
| S74 | Injury of nerves at hip and thigh level |
| T24 | Burn and corrosion of lower limb, except ankle and foot |
| L59 | Other disorders of skin and subcutaneous tissue related to radiation |
| O34 | Maternal care for abnormality of pelvic organs |
| O89 | Complications of anesthesia during the puerperium |
| T19 | Foreign body in genitourinary tract |
| O64 | Obstructed labor due to malposition and malpresentation of fetus |
| Q84 | Other congenital malformations of integument |
| Q64 | Other congenital malformations of urinary system |
| J64 | Unspecified pneumoconiosis |
| J94 | Other pleural conditions |
| N04 | Nephrotic syndrome |
| I09 | Other rheumatic heart diseases |
| D84 | Other immunodeficiencies |
| O14 | Pre-eclampsia |
| O04 | Complications following (induced) termination of pregnancy |
| A54 | Gonococcal infection |
| D04 | Carcinoma in situ of skin |
| F94 | Disorders of social functioning with onset specific to childhood and adolescence |
| E44 | Protein-calorie malnutrition of moderate and mild degree |
| Q14 | Congenital malformations of posterior segment of eye |
| B89 | Unspecified parasitic disease |
| M45 | Ankylosing spondylitis |
| J05 | Acute obstructive laryngitis [croup] and epiglottitis |
| R15 | Fecal incontinence |
| F45 | Somatoform disorders |
| M65 | Synovitis and tenosynovitis |
| N85 | Other noninflammatory disorders of uterus, except cervix |
| T13 | Other injuries of the lower extremity, level unspecified |
| M05 | Rheumatoid arthritis with rheumatoid factor |
| I15 | Secondary hypertension |
| D15 | Benign neoplasm of other and unspecified intrathoracic organs |
| R45 | Symp.* involving emotional state |
| L25 | Unspecified contact dermatitis |
| E75 | Disorders of sphingolipid metabolism and other lipid storage disorders |
| K25 | Gastric ulcer |
| R55 | Syncope and collapse |
| J45 | Asthma |
| J35 | Chronic diseases of tonsils and adenoids |
| K60 | Fissure and fistula of anal and rectal regions |
| Q70 | Syndactyly |
| N75 | Diseases of Bartholin's gland |
| R05 | Cough |
| E55 | Vitamin D deficiency |
| F15 | Other stimulant related disorders |
| E05 | Thyrotoxicosis [hyperthyroidism] |
| I85 | Esophageal varices |
| K75 | Other inflammatory liver diseases |
| B30 | Viral conjunctivitis |
| F05 | Delirium due to known physiological condition |
| H05 | Disorders of orbit |
| H95 | Intraoperative and postprocedural complications and disorders of ear and mastoid process |
| T85 | Complications of other internal prosthetic devices, implants and grafts |
| S95 | Injury of blood vessels at ankle and foot level |
| H35 | Other retinal disorders |
| L05 | Pilonidal cyst and sinus |
| L65 | Other nonscarring hair loss |
| I35 | Nonrheumatic aortic valve disorders |
| D42 | Neoplasm of uncertain behavior of meninges |
| M85 | Other disorders of bone density and structure |
| H70 | Mastoiditis and related conditions |
| J14 | Pneumonia due to Hemophilus influenzae |
| K85 | Acute pancreatitis |
| L85 | Other epidermal thickening |
| S85 | Injury of blood vessels at lower leg level |
| S05 | Injury of eye and orbit |
| S25 | Injury of blood vessels of thorax |
| F55 | Abuse of non-psychoactive substances |
| S65 | Injury of blood vessels at wrist and hand level |
| K45 | Other abdominal hernia |
| N45 | Orchitis and epididymitis |
| E25 | Adrenogenital disorders |
| O99 | Other maternal diseases classifiable elsewhere but complicating pregnancy, childbirth and the puerperium |
| S35 | Injury of blood vessels at abdomen, lower back and pelvis level |
| M35 | Other systemic involvement of connective tissue |
| O25 | Malnutrition in pregnancy, childbirth and the puerperium |
| I25 | Chronic ischemic heart disease |
| D75 | Other and unspecified diseases of blood and blood-forming organs |
| H20 | Iridocyclitis |
| F65 | Paraphilias |
| N15 | Other renal tubulo-interstitial diseases |
| K05 | Gingivitis and periodontal diseases |
| S55 | Injury of blood vessels at forearm level |
| T56 | Toxic effect of metals |
| S75 | Injury of blood vessels at hip and thigh level |
| T25 | Burn and corrosion of ankle and foot |
| O35 | Maternal care for known or suspected fetal abnormality and damage |
| O90 | Complications of the puerperium |
| O65 | Obstructed labor due to maternal pelvic abnormality |
| Q85 | Phakomatoses |
| N05 | Unspecified nephritic syndrome |
| F25 | Schizoaffective disorders |
| O15 | Eclampsia |
| O05 | Other abortion |
| D05 | Carcinoma in situ of breast |
| F95 | Tic disorder |
| E45 | Retarded development following protein-calorie malnutrition |
| Q15 | Other congenital malformations of eye |
| M46 | Other inflammatory spondylopathies |
| J06 | Acute upper respiratory infections |
| R16 | Hepatomegaly and splenomegaly |
| M66 | Spontaneous rupture of synovium and tendon |
| N86 | Erosion and ectropion of cervix uteri |
| T14 | Injury of unspecified body region |
| M06 | Other rheumatoid arthritis |
| D16 | Benign neoplasm of bone and articular cartilage |
| A06 | Amebiasis |
| R46 | Symp.* involving appearance and behavior |
| L26 | Exfoliative dermatitis |
| E76 | Disorders of glycosaminoglycan metabolism |
| K26 | Duodenal ulcer |
| R56 | Convulsions |
| J46 | Status asthmaticus |
| J36 | Peritonsillar abscess |
| K61 | Abscess of anal and rectal regions |
| Q71 | Reduction defects of upper limb |
| N76 | Other inflammation of vagina and vulva |
| R06 | Abnormalities of breathing |
| E56 | Other vitamin deficiencies |
| F16 | Hallucinogen related disorders |
| E06 | Thyroiditis |
| I86 | Varicose veins of other sites |
| K76 | Other diseases of liver |
| F06 | Other mental disorders due to known physiological condition |
| H06 | Affections of the lacrimal apparatus and orbit in diseases classified elsewhere |
| T86 | Complications of transplanted organs and tissue |
| S96 | Injury of muscle and tendon at ankle and foot level |
| H36 | Retinal disorders in diseases classified elsewhere |
| L66 | Cicatricial alopecia [scarring hair loss] |
| I36 | Nonrheumatic tricuspid valve disorders |
| D43 | Neoplasm of uncertain behavior of brain and central nervous system |
| M86 | Osteomyelitis |
| H71 | Cholesteatoma of middle ear |
| J15 | Bacterial pneumonia |
| K86 | Other diseases of pancreas |
| S86 | Injury of muscle, fascia and tendon at lower leg level |
| S06 | Intracranial injury |
| S66 | Injury of muscle, fascia and tendon at wrist and hand level |
| K46 | Unspecified abdominal hernia |
| N46 | Male infertility |
| R76 | Other abnormal immunological findings in serum |
| E26 | Hyperaldosteronism |
| S36 | Injury of intra-abdominal organs |
| S16 | Injury of muscle, fascia and tendon at neck level |
| O26 | Maternal care for other conditions predominantly related to pregnancy |
| D76 | Other specified diseases with participation of lymphoreticular and reticulohistiocytic tissue |
| R89 | Abnormal findings in specimens from other organs, systems and tissues |
| H21 | Other disorders of iris and ciliary body |
| F66 | Other sexual disorders |
| T06 | Other injuries involving multiple body regions, not elsewhere classified |
| K06 | Other disorders of gingiva and edentulous alveolar ridge |
| S46 | Injury of muscle, fascia and tendon at shoulder and upper arm level |
| S56 | Injury of muscle, fascia and tendon at forearm level |
| S76 | Injury of muscle, fascia and tendon at hip and thigh level |
| T26 | Burn and corrosion confined to eye and adnexa |
| O36 | Maternal care for other fetal problems |
| O91 | Infections of breast associated with pregnancy, the puerperium and lactation |
| O66 | Other obstructed labor |
| N06 | Isolated proteinuria with specified morphological lesion |
| D86 | Sarcoidosis |
| O16 | Unspecified maternal hypertension |
| O06 | Unspecified abortion |
| A56 | Other sexually transmitted chlamydial diseases |
| D06 | Carcinoma in situ of cervix uteri |
| A26 | Erysipeloid |
| E46 | Unspecified protein-calorie malnutrition |
| M47 | Spondylosis |
| R17 | Unspecified jaundice |
| M67 | Other disorders of synovium and tendon |
| N87 | Dysplasia of cervix uteri |
| M07 | Enteropathic arthropathies |
| D17 | Benign lipomatous neoplasm |
| A07 | Other protozoal intestinal diseases |
| L27 | Dermatitis due to substances taken internally |
| E77 | Disorders of glycoprotein metabolism |
| K27 | Peptic ulcer, site unspecified |
| R57 | Shock |
| J47 | Bronchiectasis |
| J37 | Chronic laryngitis and laryngotracheitis |
| K62 | Other diseases of anus and rectum |
| Q72 | Reduction defects of lower limb |
| N77 | Vulvovaginal ulceration and inflammation in diseases classified elsewhere |
| R07 | Pain in throat and chest |
| T73 | Effects of other deprivation |
| F17 | Nicotine dependence |
| E07 | Other disorders of thyroid |
| I87 | Other disorders of veins |
| K77 | Liver disorders in diseases classified elsewhere |
| F07 | Personality and behavioral disorders due to known physiological condition |
| T87 | Complications peculiar to reattachment and amputation |
| S97 | Crushing injury of ankle and foot |
| B07 | Viral warts |
| L67 | Hair color and hair shaft abnormalities |
| I37 | Nonrheumatic pulmonary valve disorders |
| D44 | Neoplasm of uncertain behavior of endocrine glands |
| M87 | Osteonecrosis |
| H72 | Perforation of tympanic membrane |
| J16 | Pneumonia due to other infectious organisms |
| K87 | Disorders of gallbladder, biliary tract and pancreas in diseases classified elsewhere |
| L87 | Transepidermal elimination disorders |
| S07 | Crushing injury of head |
| S27 | Injury of other and unspecified intrathoracic organs |
| S67 | Crushing injury of wrist, hand and fingers |
| N47 | Disorders of prepuce |
| R77 | Other abnormalities of plasma proteins |
| A37 | Whooping cough |
| I77 | Other disorders of arteries and arterioles |
| E27 | Other disorders of adrenal gland |
| S37 | Injury of urinary and pelvic organs |
| D77 | Other disorders of blood and blood-forming organs in diseases classified elsewhere |
| H22 | Disorders of iris and ciliary body in diseases classified elsewhere |
| T07 | Unspecified multiple injuries |
| K07 | Dentofacial abnormalities [including malocclusion] |
| S57 | Crushing injury of elbow and forearm |
| T58 | Toxic effect of carbon monoxide |
| O92 | Other disorders of breast and disorders of lactation associated with pregnancy and the puerperium |
| Q87 | Other specified congenital malformation syndromes affecting multiple systems |
| J67 | Hypersensitivity pneumonitis due to organic dust |
| D07 | Carcinoma in situ of other and unspecified genital organs |
| Q17 | Other congenital malformations of ear |
| M48 | Other spondylopathies |
| R18 | Ascites |
| F48 | Other nonpsychotic mental disorders |
| M68 | Diseases of the synovium and tendons in diseases classified elsewhere |
| N88 | Other noninflammatory disorders of cervix uteri |
| D18 | Hemangioma and lymphangioma, any site |
| A08 | Viral and other specified intestinal infections |
| L28 | Lichen simplex chronicus and prurigo |
| E78 | Disorders of lipoprotein metabolism and other lipidemias |
| K28 | Gastrojejunal ulcer |
| R58 | Hemorrhage |
| J38 | Diseases of vocal cords and larynx |
| K63 | Other diseases of intestine |
| Q73 | Reduction defects of unspecified limb |
| E58 | Dietary calcium deficiency |
| T74 | Adult and child abuse, neglect and other maltreatment, confirmed |
| F18 | Inhalant related disorders |
| I88 | Nonspecific lymphadenitis |
| B33 | Other viral diseases |
| T88 | Other complications of surgical and medical care |
| S98 | Traumatic amputation of ankle and foot |
| B08 | Other viral infections characterized by skin and mucous membrane lesions |
| L08 | Other local infections of skin and subcutaneous tissue |
| L68 | Hypertrichosis |
| I38 | Endocarditis, valve unspecified |
| D45 | Polycythemia vera |
| H73 | Other disorders of tympanic membrane |
| L88 | Pyoderma gangrenosum |
| S68 | Traumatic amputation of wrist, hand and fingers |
| N48 | Other disorders of penis |
| R78 | Findings of drugs and other substances, not normally found in blood |
| A38 | Scarlet fever |
| I78 | Diseases of capillaries |
| E28 | Ovarian dysfunction |
| O28 | Abnormal findings on antenatal screening of mother |
| F68 | Other disorders of adult personality and behavior |
| K08 | Other disorders of teeth and supporting structures |
| C08 | Malignant neoplasm of other and unspecified major salivary glands |
| S58 | Traumatic amputation of elbow and forearm |
| T59 | Toxic effect of other gases, fumes and vapors |
| O68 | Labor and delivery complicated by abnormality of fetal acid-base balance |
| J68 | Respiratory conditions due to inhalation of chemicals, gases, fumes and vapors |
| N08 | Glomerular disorders in diseases classified elsewhere |
| F28 | Other psychotic disorder not due to a substance or known physiological condition |
| O08 | Complications following ectopic and molar pregnancy |
| F98 | Other behavioral and emotional disorders with onset usually occurring in childhood and adolescence |
| A28 | Other zoonotic bacterial diseases |
| Q18 | Other congenital malformations of face and neck |
| M49 | Spondylopathies in diseases classified elsewhere |
| R19 | Other symp.* involving the digestive system and abdomen |
| N89 | Other noninflammatory disorders of vagina |
| M09 | Juvenile arthritis in diseases classified elsewhere |
| A09 | Infectious gastroenteritis and colitis, unspecified |
| L29 | Pruritus |
| E79 | Disorders of purine and pyrimidine metabolism |
| K29 | Gastritis and duodenitis |
| R59 | Enlarged lymph nodes |
| J39 | Other diseases of upper respiratory tract |
| K64 | Hemorrhoids and perianal venous thrombosis |
| Q74 | Other congenital malformations of limb(s) |
| R09 | Other symp.* involving the circulatory and respiratory system |
| E59 | Dietary selenium deficiency |
| T75 | Other and unspecified effects of other external causes |
| F19 | Other psychoactive substance related disorders |
| B44 | Aspergillosis |
| I89 | Other noninfective disorders of lymphatic vessels and lymph nodes |
| B34 | Viral infection of unspecified site |
| F09 | Unspecified mental disorder due to known physiological condition |
| S99 | Other and unspecified injuries of ankle and foot |
| B09 | Unspecified viral infection characterized by skin and mucous membrane lesions |
| D46 | Myelodysplastic syndromes |
| M89 | Other disorders of bone |
| H74 | Other disorders of middle ear mastoid |
| J18 | Pneumonia, unspecified organism |
| L89 | Pressure ulcer |
| S89 | Other and unspecified injuries of lower leg |
| S09 | Other and unspecified injuries of head |
| S29 | Other and unspecified injuries of thorax |
| F59 | Unspecified behavioral syndromes associated with physiological disturbances and physical factors |
| S69 | Other and unspecified injuries of wrist, hand and finger(s) |
| N49 | Inflammatory disorders of male genital organs |
| R79 | Other abnormal findings of blood chemistry |
| A39 | Meningococcal infection |
| I79 | Disorders of arteries, arterioles and capillaries in diseases classified elsewhere |
| E29 | Testicular dysfunction |
| S39 | Other and unspecified injuries of abdomen, lower back, pelvis and external genitals |
| F69 | Unspecified disorder of adult personality and behavior |
| K09 | Cysts of oral region |
| S49 | Other and unspecified injuries of shoulder and upper arm |
| C09 | Malignant neoplasm of tonsil |
| T60 | Toxic effect of pesticides |
| S79 | Other and unspecified injuries of hip and thigh |
| T29 | Burns or chemical burns affecting several body regions |
| O69 | Labor and delivery complicated by umbilical cord complications |
| Q89 | Other congenital malformations |
| J69 | Pneumonitis due to solids and liquids |
| F29 | Unspecified psychosis not due to a substance or known physiological condition |
| D89 | Other disorders involving the immune mechanism |
| A59 | Trichomoniasis |
| D09 | Carcinoma in situ of other and unspecified sites |
| M50 | Cervical disc disorders |
| M70 | Soft tissue disorders related to use, overuse and pressure |
| N90 | Other noninflammatory disorders of vulva and perineum |
| M10 | Gout |
| L30 | Other and unspecified dermatitis |
| E80 | Disorders of porphyrin and bilirubin metabolism |
| K30 | Functional dyspepsia |
| R60 | Edema |
| Q75 | Other congenital malformations of skull and face bones |
| E60 | Dietary zinc deficiency |
| L70 | Acne |
| I40 | Acute myocarditis |
| D47 | Other neoplasms of uncertain behavior of lymphoid, hematopoietic and related tissue |
| M90 | Osteopathies in diseases classified elsewhere |
| L90 | Atrophic disorders of skin |
| N50 | Other and unspecified disorders of male genital organs |
| A40 | Streptococcal sepsis |
| K10 | Other diseases of the jaw |
| C10 | Malignant neoplasm of oropharynx |
| T61 | Toxic effect of noxious substances eaten as seafood |
| T30 | Burn and corrosion, body region unspecified |
| O40 | Polyhydramnios |
| O70 | Perineal laceration during delivery |
| J70 | Respiratory conditions due to other external agents |
| A60 | Anogenital herpesviral [herpes simplex] infections |
| M51 | Thoracic, thoracolumbar, and lumbosacral intervertebral disc disorders |
| M71 | Other bursopathies |
| N91 | Absent, scanty and rare menstruation |
| M11 | Other crystal arthropathies |
| D21 | Other benign neoplasms of connective and other soft tissue |
| K31 | Other diseases of stomach and duodenum |
| R61 | Generalized hyperhidrosis |
| Q76 | Congenital malformations of spine and bony thorax |
| E61 | Deficiency of other nutrient elements |
| B46 | Zygomycosis |
| L71 | Rosacea |
| I41 | Myocarditis in diseases classified elsewhere |
| D48 | Neoplasm of uncertain behavior of other and unspecified sites |
| M91 | Juvenile osteochondrosis of hip and pelvis |
| L91 | Hypertrophic disorders of skin |
| N51 | Disorders of male genital organs in diseases classified elsewhere |
| A41 | Other sepsis |
| E31 | Polyglandular dysfunction |
| K11 | Diseases of salivary glands |
| C11 | Malignant neoplasm of nasopharynx |
| T62 | Toxic effect of other noxious substances eaten as food |
| T31 | Burns classified according to extent of body surface involved |
| O41 | Other disorders of amniotic fluid and membranes |
| O71 | Other obstetric trauma |
| M72 | Fibroblastic disorders |
| N92 | Excessive, frequent and irregular menstruation |
| M12 | Other and unspecified arthropathy |
| D22 | Melanocytic nevi |
| T78 | Adverse effects |
| L72 | Follicular cysts of skin and subcutaneous tissue |
| I42 | Cardiomyopathy |
| M92 | Other juvenile osteochondrosis |
| L92 | Granulomatous disorders of skin and subcutaneous tissue |
| A42 | Actinomycosis |
| E32 | Diseases of thymus |
| K12 | Stomatitis and related lesions |
| T63 | Toxic effect of contact with venomous animals and plants |
| O42 | Premature rupture of membranes |
| O72 | Postpartum hemorrhage |
| M53 | Other and unspecified dorsopathies |
| M73 | Soft tissue disorders in diseases classified elsewhere |
| N93 | Other abnormal uterine and vaginal bleeding |
| M13 | Other arthritis |
| D23 | Other benign neoplasms of skin |
| E83 | Disorders of mineral metabolism |
| R63 | Symp.* concerning food and fluid intake |
| Q78 | Other osteochondrodysplasias |
| E63 | Other nutritional deficiencies |
| B48 | Other mycoses |
| L73 | Other follicular disorders |
| I43 | Cardiomyopathy in diseases classified elsewhere |
| M93 | Other osteochondropathies |
| L93 | Lupus erythematosus |
| K13 | Other diseases of lip and oral mucosa |
| C13 | Malignant neoplasm of hypopharynx |
| O43 | Placental disorders |
| O73 | Retained placenta and membranes, without hemorrhage |
| A63 | Other predominantly sexually transmitted diseases |
| M54 | Dorsalgia |
| N94 | Pain and other conditions associated with female genital organs and menstrual cycle |
| M14 | Arthropathies in other diseases classified elsewhere |
| D24 | Benign neoplasm of breast |
| R64 | Cachexia |
| Q79 | Congenital malformations of musculoskeletal system |
| E64 | Sequelae of malnutrition and other nutritional deficiencies |
| B49 | Unspecified mycosis |
| L74 | Eccrine sweat disorders |
| I44 | Atrioventricular and left bundle-branch block |
| M94 | Other disorders of cartilage |
| L94 | Other localized connective tissue disorders |
| E34 | Other endocrine disorders |
| K14 | Diseases of tongue |
| T65 | Toxic effect of other and unspecified substances |
| O44 | Placenta previa |
| O74 | Complications of anesthesia during labor and delivery |
| A64 | Unspecified sexually transmitted disease |
| M75 | Shoulder lesions |
| N95 | Menopausal and other perimenopausal disorders |
| M15 | Polyosteoarthritis |
| D25 | Leiomyoma of uterus |
| E85 | Amyloidosis |
| R65 | Symp.* specifically associated with systemic inflammation and infection |
| I45 | Other conduction disorders |
| L95 | Vasculitis limited to skin |
| E35 | Disorders of endocrine glands in diseases classified elsewhere |
| C15 | Malignant neoplasm of esophagus |
| O75 | Other complications of labor and delivery |
| M76 | Enthesopathies, lower limb, excluding foot |
| N96 | Recurrent pregnancy loss |
| M16 | Osteoarthritis of hip |
| D26 | Other benign neoplasms of uterus |
| E86 | Volume depletion |
| I46 | Cardiac arrest |
| A46 | Erysipelas |
| C16 | Malignant neoplasm of stomach |
| O46 | Antepartum hemorrhage |
| M77 | Other enthesopathies |
| N97 | Female infertility |
| M17 | Osteoarthritis of knee |
| D27 | Benign neoplasm of ovary |
| E87 | Other disorders of fluid, electrolyte and acid-base balance |
| I47 | Paroxysmal tachycardia |
| L97 | Non-pressure chronic ulcer of lower limb |
| C17 | Malignant neoplasm of small intestine |
| O47 | False labor |
| N98 | Complications associated with artificial fertilization |
| M18 | Osteoarthritis of first carpometacarpal joint |
| D28 | Benign neoplasm of other and unspecified female genital organs |
| E88 | Other and unspecified metabolic disorders |
| R68 | Other general symp.* |
| I48 | Atrial fibrillation and flutter |
| L98 | Other disorders of skin and subcutaneous tissue |
| A48 | Other bacterial diseases |
| C18 | Malignant neoplasm of colon |
| O48 | Late pregnancy |
| M79 | Other and unspecified soft tissue disorders |
| M19 | Other and unspecified osteoarthritis |
| D29 | Benign neoplasm of male genital organs |
| E89 | Postprocedural endocrine and metabolic complications and disorders |
| R69 | Illness, unspecified |
| I49 | Other cardiac arrhythmias |
| L99 | Other disorders of skin and subcutaneous tissue in diseases classified elsewhere |
| A49 | Bacterial infection of unspecified site |
| C19 | Malignant neoplasm of rectosigmoid junction |
| M20 | Acquired deformities of fingers and toes |
| D30 | Benign neoplasm of urinary organs |
| E90 | Nutritional and metabolic disorders in diseases classified elsewhere |
| I50 | Heart failure |
| C20 | Malignant neoplasm of rectum |
| M21 | Other acquired deformities of limbs |
| D31 | Benign neoplasm of eye and adnexa |
| I51 | Complications and ill-defined descriptions of heart disease |
| C21 | Malignant neoplasm of anus and anal canal |
| M22 | Disorder of patella |
| D32 | Benign neoplasm of meninges |
| C22 | Malignant neoplasm of liver and intrahepatic bile ducts |
| M23 | Internal derangement of knee |
| D33 | Benign neoplasm of brain and other parts of central nervous system |
| M24 | Other specific joint derangements |
| D34 | Benign neoplasm of thyroid gland |
| C24 | Malignant neoplasm of other and unspecified parts of biliary tract |
| M25 | Other joint disorder |
| D35 | Benign neoplasm of other and unspecified endocrine glands |
| C25 | Malignant neoplasm of pancreas |
| D36 | Benign neoplasm of other and unspecified sites |
| C26 | Malignant neoplasm of other and ill-defined digestive organs |
| C30 | Malignant neoplasm of nasal cavity and middle ear |
| C32 | Malignant neoplasm of larynx |
| C34 | Malignant neoplasm of bronchus and lung |
| C38 | Malignant neoplasm of heart, mediastinum and pleura |
| C40 | Malignant neoplasm of bone and articular cartilage of limbs |
| C41 | Malignant neoplasm of bone and articular cartilage of other and unspecified sites |
| C43 | Malignant melanoma of skin |
| C44 | Other and unspecified malignant neoplasm of skin |
| C45 | Mesothelioma |
| C46 | Kaposi's sarcoma |
| C47 | Malignant neoplasm of peripheral nerves and autonomic nervous system |
| C49 | Malignant neoplasm of other connective and soft tissue |
| C50 | Malignant neoplasm of breast |
| C51 | Malignant neoplasm of vulva |
| C53 | Malignant neoplasm of cervix uteri |
| C54 | Malignant neoplasm of corpus uteri |
| C55 | Malignant neoplasm of uterus, part unspecified |
| C56 | Malignant neoplasm of ovary |
| C57 | Malignant neoplasm of other and unspecified female genital organs |
| C58 | Malignant neoplasm of placenta |
| C60 | Malignant neoplasm of penis |
| C61 | Malignant neoplasm of prostate |
| C62 | Malignant neoplasm of testis |
| C64 | Malignant neoplasm of kidney, except renal pelvis |
| C66 | Malignant neoplasm of ureter |
| C67 | Malignant neoplasm of bladder |
| C68 | Malignant neoplasm of other and unspecified urinary organs |
| C69 | Malignant neoplasm of eye and adnexa |
| C70 | Malignant neoplasm of meninges |
| C71 | Malignant neoplasm of brain |
| C72 | Malignant neoplasm of spinal cord, cranial nerves and other parts of central nervous system |
| C73 | Malignant neoplasm of thyroid gland |
| C75 | Malignant neoplasm of other endocrine glands and related structures |
| C76 | Malignant neoplasm of other and ill-defined sites |
| C77 | Secondary and unspecified malignant neoplasm of lymph nodes |
| C78 | Secondary malignant neoplasm of respiratory and digestive organs |
| C79 | Secondary malignant neoplasm of other and unspecified sites |
| C80 | Malignant neoplasm without specification of site |
| C81 | Hodgkin lymphoma |
| C82 | Follicular lymphoma |
| C83 | Non-follicular lymphoma |
| C84 | Mature T/NK-cell lymphomas |
| C85 | Other specified and unspecified types of non-Hodgkin lymphoma |
| C86 | Other specified types of T/NK-cell lymphoma |
| C90 | Multiple myeloma and malignant plasma cell neoplasms |
| C91 | Lymphoid leukemia |
| C92 | Myeloid leukemia |
| C94 | Other leukemias of specified cell type |
| C95 | Leukemia of unspecified cell type |
| C96 | Other and unspecified malignant neoplasms of lymphoid, hematopoietic and related tissue |
| C97 | Malignant neoplasms as primary tumors at multiple locations |

### Table S3: The list of disease-modifying therapies (DMTs) used in the prediction models and their category based on the effectiveness

| **DMT** | **Category** |
| --- | --- |
| Dimethyl Fumarate | Moderate Efficacy |
| Glatiramer Acetate |  |
| Interferon_beta-1a |  |
| Interferon_beta-1b |  |
| Peginterferon_beta-1a |  |
| Teriflunomide |  |
| Cladribine | High Efficacy |
| Fingolimod |  |
| Alemtuzumab | Very High Efficacy |
| Daclizumab |  |
| Mitoxantrone |  |
| Natalizumab |  |
| Ocrelizumab |  |
| Rituximab |  |

### Table S4: Assessment criteria for determining the need for nursing care

| **Criteria** | **Definition** |
| --- | --- |
| Mobility | Changing position in bed, maintaining a stable sitting position, transferring, moving around within the living area, climbing stairs |
| Cognitive and communicative abilities | Recognizing people from the immediate environment, local orientation, temporal orientation, remembering significant events or observations, controlling multi-step everyday actions, making decisions in everyday life, understanding facts and information, recognizing risks and dangers, communicating elementary needs, understanding prompts, participating in a conversation |
| Behaviour and psychological problems | Motor behavioral abnormalities, nocturnal restlessness, self-harming and auto-aggressive behavior, damaging objects, physically aggressive behavior towards other people, verbal aggression, other care-relevant vocal abnormalities, resistance to nursing and other supportive measures, delusions, fears, listlessness in depressive moods, socially inadequate behavior, other care-relevant inadequate actions |
| Self-care | Washing the front of the upper body, personal hygiene around the head, washing the genital area, showering and bathing (including washing hair), dressing and undressing the upper body, dressing and undressing the lower body, preparing food in bite-sized portions and pouring drinks, eating, drinking, using a toilet or commode chair, coping with the consequences of urinary incontinence and dealing with a urinary catheter and urostomy, coping with the consequences of fecal incontinence and dealing with a stoma, parenteral or tube feeding, existence of serious feeding problems in children up to 18 months of age that require exceptional care |
| Coping with and independent handling of illness- or therapy-related demands and stresses | (a) with respect to medication, injections, provision of intravenous access, suctioning and oxygen administration, embrocation and cold and heat applications, measurement and interpretation of body conditions, body-related aids, b) with respect to dressing changes and wound care, stoma care, regular one-time catheterization and use of laxative methods, therapeutic measures in the home environment, c) with respect to time-consuming and technology-intensive measures in the home environment, visits to the doctor, visits to other medical or therapeutic facilities, extended visits to medical or therapeutic facilities, visits to early intervention facilities for children, and d) with respect to adherence to a diet or other illness- or therapy-related behavioral regulations |
| Organization of everyday life and social contacts | Organizing daily routines and adapting to changes, resting and sleeping, keeping busy, making plans for the future, interacting with people in direct contact, maintaining contact with people outside the immediate environment. |

### Table S5: Levels of nursing care assigned to the individuals

| **Care Level** | **Definition** |
| --- | --- |
| Level 1 | Minor impairment of independence or abilities, |
| Level 2 | Significant impairment of independence or abilities, |
| Level 3 | Severe impairment of independence or abilities, |
| Level 4 | Extremely severe impairment of independence or abilities, |
| Level 5 | Extremely severe impairment of independence or abilities with special care requirements. |

### Table S6: List of hyperparameters used for training the prediction model

| **Hyperparameter** | **Definition** | **Search Range** |
| --- | --- | --- |
| eta | Learning rate | [0.001, 0.2] |
| max_depth | Maximum depth for tree model | [2, 8] |
| num_leaves | Maximum number of leaves in one tree | [3, 256] |
| Min_data | Minimal number of data in one leaf | [5, 150] |
| min_hessian | Minimal sum hessian in one leaf | [0.01, 20] |
| path smooth | Controls smoothing applied to tree nodes | [0, 10] |
| lambda_l1 | L1 regularization | [0, 20] |
| lambda_l2 | L2 regularization | [0, 20] |
| min_gain_to_split | The minimal gain to perform split | [0, 20] |
| bagging_fraction | Fraction of samples selected on each iteration | [0.5, 1] |
| bagging_freq | Frequency of bagging | [25, 100] |
| feature_fraction | Fraction of features selected on each iteration | [0.1, 1] |

### Table S7: The comparisons of MS, CD, RA, and CTR for each age group, with respect to all healthcare parameters included in the study.

|  |  | **20-29** | | **30-39** | | **40-49** | |
| --- | --- | --- | --- | --- | --- | --- | --- |
| **Category** | **Phenotype** | **1/OR [95% CI]** | **Adjusted p-value** | **1/OR [95% CI]** | **Adjusted p-value** | **1/OR [95% CI]** | **Adjusted p-value** |
| Rate of Assistive Device Usage | CD | 1.64 [1.32;2.04] | p = 0.001 | 4.17 [3.12;5.26] | p < 1e-3 | 16.67 [12.5;20] | p < 1e-3 |
|  | RA | 1.47 [1.14;1.89] | p = 0.199 | 5.26 [3.85;6.67] | p < 1e-3 | 14.29 [11.11;16.67] | p < 1e-3 |
|  | CTR | 2.22 [1.79;2.7] | p < 1e-3 | 7.69 [5.88;10] | p < 1e-3 | 25 [20;25] | p < 1e-3 |
| Number of Different Assistive Devices Used | CD | 1.32 [1.15;1.52] | p = 0.01 | 2.27 [1.96;2.63] | p < 1e-3 | 4.35 [3.85;5] | p < 1e-3 |
|  | RA | 1.08 [0.93;1.25] | p = 1 | 1.56 [1.35;1.79] | p < 1e-3 | 3.03 [2.78;3.33] | p < 1e-3 |
|  | CTR | 1.59 [1.39;1.82] | p < 1e-3 | 3.45 [3.03;4] | p < 1e-3 | 6.67 [5.88;7.14] | p < 1e-3 |
| Using any Assistive Device at Least Once | CD | 1.39 [1.14;1.69] | p = 0.138 | 1.82 [1.56;2.13] | p < 1e-3 | 3.12 [2.7;3.57] | p < 1e-3 |
|  | RA | 1.03 [0.83;1.28] | p = 1 | 1.18 [1.02;1.37] | p = 1 | 2.17 [1.96;2.44] | p < 1e-3 |
|  | CTR | 1.61 [1.35;1.96] | p < 1e-3 | 2.7 [2.38;3.03] | p < 1e-3 | 4.55 [4.17;5] | p < 1e-3 |
| Receiving Nursing Care | CD | 2.04 [1.05;4] | p = 1 | 5.56 [3.85;7.69] | p < 1e-3 | 9.09 [7.14;12.5] | p < 1e-3 |
|  | RA | 1.33 [0.67;2.63] | p = 1 | 3.33 [2.44;4.55] | p < 1e-3 | 7.14 [5.88;8.33] | p < 1e-3 |
|  | CTR | 2.27 [1.25;4.17] | p = 0.577 | 5.56 [4.35;7.14] | p < 1e-3 | 14.29 [11.11;16.67] | p < 1e-3 |
| Rate of Participating in Any Remedy | CD | 2.13 [1.75;2.63] | p < 1e-3 | 2.38 [2.13;2.7] | p < 1e-3 | 4 [3.7;4.55] | p < 1e-3 |
|  | RA | 1.02 [0.82;1.27] | p = 1 | 1.33 [1.19;1.52] | p < 1e-3 | 2.63 [2.38;2.86] | p < 1e-3 |
|  | CTR | 3.23 [2.7;3.85] | p < 1e-3 | 4 [3.57;4.35] | p < 1e-3 | 5.88 [5.56;6.67] | p < 1e-3 |
| Rate of Receiving Sick Leave | CD | 1.08 [0.92;1.25] | p = 1 | 1.09 [0.99;1.18] | p = 1 | 1.09 [0.98;1.19] | p = 1 |
|  | RA | 2.38 [2;2.86] | p < 1e-3 | 2.22 [2;2.44] | p < 1e-3 | 1.89 [1.72;2.04] | p < 1e-3 |
|  | CTR | 1.11 [0.96;1.28] | p = 1 | 1.08 [1;1.16] | p = 1 | 0.9 [0.83;0.97] | p = 0.43 |
| Duration of Sick Leave Received | CD | 1.22 [0.97;1.52] | p = 1 | 1.39 [1.22;1.56] | p < 1e-3 | 1.45 [1.25;1.67] | p < 1e-3 |
|  | RA | 2.5 [1.96;3.12] | p < 1e-3 | 2.5 [2.17;2.78] | p < 1e-3 | 2.27 [2;2.56] | p < 1e-3 |
|  | CTR | 1.67 [1.35;2] | p < 1e-3 | 1.82 [1.64;2.04] | p < 1e-3 | 1.47 [1.3;1.64] | p < 1e-3 |
| Rate of Receiving Rehabilitation | CD | 1.89 [1.49;2.44] | p < 1e-3 | 1.85 [1.64;2.13] | p < 1e-3 | 2.13 [1.92;2.33] | p < 1e-3 |
|  | RA | 1.75 [1.33;2.33] | p = 0.004 | 1.72 [1.52;1.96] | p < 1e-3 | 1.64 [1.52;1.79] | p < 1e-3 |
|  | CTR | 5.56 [4.35;7.14] | p < 1e-3 | 4.76 [4.17;5.26] | p < 1e-3 | 4 [3.7;4.35] | p < 1e-3 |
| Duration of Rehabilitation Received | CD | 2.78 [1.39;5.56] | p = 0.266 | 2.17 [1.56;3.03] | p < 1e-3 | 2.17 [1.72;2.78] | p < 1e-3 |
|  | RA | 2.86 [1.33;6.25] | p = 0.443 | 2 [1.43;2.78] | p = 0.003 | 1.82 [1.47;2.27] | p < 1e-3 |
|  | CTR | 6.67 [3.45;12.5] | p < 1e-3 | 5 [3.85;6.67] | p < 1e-3 | 4 [3.33;4.76] | p < 1e-3 |

|  |  | **50-59** | | **60-69** | | **70-79** | | **80+** | |
| --- | --- | --- | --- | --- | --- | --- | --- | --- | --- |
| **Category** | **Phenotype** | **1/OR [95% CI]** | **Adjusted p-value** | **1/OR [95% CI]** | **Adjusted p-value** | **1/OR [95% CI]** | **Adjusted p-value** | **1/OR [95% CI]** | **Adjusted p-value** |
| Rate of Assistive Device Usage | CD | 12.5 [11.11;16.67] | p < 1e-3 | 16.67 [12.5;20] | p < 1e-3 | 8.33 [7.14;10] | p < 1e-3 | 4.35 [3.45;5.56] | p < 1e-3 |
|  | RA | 20 [16.67;20] | p < 1e-3 | 16.67 [14.29;20] | p < 1e-3 | 11.11 [9.09;12.5] | p < 1e-3 | 5.26 [4.35;6.67] | p < 1e-3 |
|  | CTR | 20 [20;25] | p < 1e-3 | 20 [16.67;25] | p < 1e-3 | 12.5 [10;14.29] | p < 1e-3 | 5.56 [4.55;6.67] | p < 1e-3 |
| Number of Different Assistive Devices Used | CD | 4.35 [4;4.76] | p < 1e-3 | 4.55 [4.17;5] | p < 1e-3 | 3.03 [2.7;3.33] | p < 1e-3 | 1.85 [1.67;2.04] | p < 1e-3 |
|  | RA | 3.85 [3.7;4.17] | p < 1e-3 | 4 [3.7;4.35] | p < 1e-3 | 3.12 [2.86;3.45] | p < 1e-3 | 1.85 [1.69;2.04] | p < 1e-3 |
|  | CTR | 6.67 [6.25;7.14] | p < 1e-3 | 6.25 [5.88;6.67] | p < 1e-3 | 4.17 [3.85;4.55] | p < 1e-3 | 2.17 [1.96;2.38] | p < 1e-3 |
| Using any Assistive Device at Least Once | CD | 3.57 [3.23;3.85] | p < 1e-3 | 5.26 [4.55;5.88] | p < 1e-3 | 6.67 [5.56;8.33] | p < 1e-3 | 5 [3.12;8.33] | p < 1e-3 |
|  | RA | 2.94 [2.7;3.12] | p < 1e-3 | 4.35 [3.85;4.76] | p < 1e-3 | 7.14 [5.88;9.09] | p < 1e-3 | 5.56 [3.45;9.09] | p < 1e-3 |
|  | CTR | 5.26 [5;5.88] | p < 1e-3 | 7.69 [6.67;8.33] | p < 1e-3 | 11.11 [9.09;14.29] | p < 1e-3 | 7.69 [4.76;12.5] | p < 1e-3 |
| Receiving Nursing Care | CD | 8.33 [7.14;10] | p < 1e-3 | 11.11 [9.09;12.5] | p < 1e-3 | 7.69 [6.25;9.09] | p < 1e-3 | 5.56 [4;8.33] | p < 1e-3 |
|  | RA | 10 [9.09;11.11] | p < 1e-3 | 11.11 [10;12.5] | p < 1e-3 | 9.09 [7.69;10] | p < 1e-3 | 5.56 [4;7.69] | p < 1e-3 |
|  | CTR | 14.29 [12.5;14.29] | p < 1e-3 | 14.29 [12.5;16.67] | p < 1e-3 | 12.5 [10;14.29] | p < 1e-3 | 7.14 [5;10] | p < 1e-3 |
| Rate of Participating in Any Remedy | CD | 5 [4.55;5.26] | p < 1e-3 | 5.26 [4.76;5.88] | p < 1e-3 | 5.88 [5;6.67] | p < 1e-3 | 5.56 [4.35;6.67] | p < 1e-3 |
|  | RA | 3.45 [3.12;3.57] | p < 1e-3 | 3.85 [3.45;4.17] | p < 1e-3 | 4.76 [4.17;5.26] | p < 1e-3 | 4.55 [3.7;5.56] | p < 1e-3 |
|  | CTR | 6.67 [6.25;7.14] | p < 1e-3 | 6.67 [6.25;7.69] | p < 1e-3 | 7.69 [6.67;8.33] | p < 1e-3 | 6.25 [5;7.69] | p < 1e-3 |
| Rate of Receiving Sick Leave | CD | 0.94 [0.84;1.08] | p = 1 | - | - | - | - | - | - |
|  | RA | 1.54 [1.39;1.69] | p < 1e-3 | - | - | - | - | - | - |
|  | CTR | 0.75 [0.67;0.82] | p < 1e-3 | - | - | - | - | - | - |
| Duration of Sick Leave Received | CD | 1.27 [1.06;1.52] | p = 0.379 | - | - | - | - | - | - |
|  | RA | 1.67 [1.45;1.96] | p < 1e-3 | - | - | - | - | - | - |
|  | CTR | 1.05 [0.91;1.22] | p = 1 | - | - | - | - | - | - |
| Rate of Receiving Rehabilitation | CD | 1.72 [1.61;1.85] | p < 1e-3 | 1.43 [1.32;1.56] | p < 1e-3 | 1.49 [1.3;1.69] | p < 1e-3 | - | - |
|  | RA | 1.32 [1.23;1.39] | p < 1e-3 | 1.09 [1.02;1.18] | p = 0.725 | 1.32 [1.18;1.47] | p < 1e-3 | - | - |
|  | CTR | 2.86 [2.7;3.03] | p < 1e-3 | 2 [1.85;2.13] | p < 1e-3 | 2.04 [1.82;2.33] | p < 1e-3 | - | - |
| Duration of Rehabilitation Received | CD | 1.82 [1.56;2.13] | p < 1e-3 | 1.49 [1.27;1.79] | p < 1e-3 | 1.69 [1.67;1.75] | p < 1e-3 | - | - |
|  | RA | 1.41 [1.23;1.61] | p < 1e-3 | 1.16 [1;1.33] | p = 1 | 1.47 [1.45;1.49] | p < 1e-3 | - | - |
|  | CTR | 2.86 [2.56;3.33] | p < 1e-3 | 2.08 [1.79;2.38] | p < 1e-3 | 2.33 [2.27;2.38] | p < 1e-3 | - | - |

### Table S8: The comparisons of MS, CD, RA, and CTR for each age group, with respect to all assistive devices included in the study.

|  |  | **20-29** | | **30-39** | | **40-49** | |
| --- | --- | --- | --- | --- | --- | --- | --- |
| **Category** | **Phenotype** | **1/OR [95% CI]** | **Adjusted p-value** | **1/OR [95% CI]** | **Adjusted p-value** | **1/OR [95% CI]** | **Adjusted p-value** |
| Aids_Mobility | CD | 1.41 [1.11;1.82] | p = 1 | 1.85 [1.59;2.17] | p < 1e-3 | 3.85 [3.33;4.35] | p < 1e-3 |
|  | RA | 0.82 [0.64;1.05] | p = 1 | 1.1 [0.94;1.28] | p = 1 | 2.17 [1.92;2.44] | p < 1e-3 |
|  | CTR | 1.75 [1.39;2.22] | p = 0.001 | 2.56 [2.22;2.94] | p < 1e-3 | 4.76 [4.35;5.26] | p < 1e-3 |
| Incontinence | CD | 1.64 [0.94;2.86] | p = 1 | 3.33 [2.38;4.55] | p < 1e-3 | 4.76 [3.7;5.88] | p < 1e-3 |
|  | RA | 4.55 [1.89;11.11] | p = 0.452 | 5.88 [4;9.09] | p < 1e-3 | 8.33 [6.67;11.11] | p < 1e-3 |
|  | CTR | 6.25 [3.23;12.5] | p < 1e-3 | 8.33 [6.25;11.11] | p < 1e-3 | 16.67 [12.5;20] | p < 1e-3 |
| Nursing.items | CD | 0.62 [0.1;4] | p = 1 | 1.3 [0.47;3.57] | p = 1 | 8.33 [3.57;20] | p < 1e-3 |
|  | RA | Inf [0;Inf] | p = 1 | 9.09 [1.19;100] | p = 1 | 4.55 [2.7;7.69] | p < 1e-3 |
|  | CTR | 1.89 [0.28;12.5] | p = 1 | 16.67 [3.57;100] | p = 0.223 | 25 [12.5;50] | p < 1e-3 |
| Visual | CD | 1.18 [0.9;1.54] | p = 1 | 1.64 [1.05;2.56] | p = 1 | 1.82 [1.28;2.56] | p = 0.438 |
|  | RA | 1.1 [0.82;1.49] | p = 1 | 0.97 [0.65;1.47] | p = 1 | 1.85 [1.39;2.5] | p = 0.02 |
|  | CTR | 1.3 [1.02;1.67] | p = 1 | 2.04 [1.41;2.94] | p = 0.097 | 2.5 [1.92;3.23] | p < 1e-3 |
| Toilet | CD | 2.5 [0.83;7.69] | p = 1 | 1.79 [1.03;3.12] | p = 1 | 6.25 [4;10] | p < 1e-3 |
|  | RA | 1.22 [0.41;3.57] | p = 1 | 1.23 [0.75;2.04] | p = 1 | 2.63 [2;3.45] | p < 1e-3 |
|  | CTR | 10 [2.63;33.33] | p = 0.405 | 5 [2.94;8.33] | p < 1e-3 | 12.5 [8.33;16.67] | p < 1e-3 |
| Care.aids | CD | 0.91 [0.28;3.03] | p = 1 | 7.14 [3.12;16.67] | p = 0.001 | 14.29 [8.33;25] | p < 1e-3 |
|  | RA | 2.13 [0.43;11.11] | p = 1 | 6.25 [2.78;14.29] | p = 0.006 | 12.5 [8.33;16.67] | p < 1e-3 |
|  | CTR | 3.03 [0.83;11.11] | p = 1 | 6.67 [4;11.11] | p < 1e-3 | 25 [16.67;33.33] | p < 1e-3 |
| Body.care_hygiene | CD | 2 [0.58;7.14] | p = 1 | 5 [2.27;11.11] | p = 0.031 | 16.67 [7.69;33.33] | p < 1e-3 |
|  | RA | 4.17 [0.65;25] | p = 1 | 5.56 [2.38;12.5] | p = 0.053 | 6.25 [4.17;9.09] | p < 1e-3 |
|  | CTR | 2.38 [0.76;7.69] | p = 1 | 7.14 [4;14.29] | p < 1e-3 | 20 [12.5;33.33] | p < 1e-3 |

|  |  | **50-59** | | **60-69** | | **70-79** | | **80+** | |
| --- | --- | --- | --- | --- | --- | --- | --- | --- | --- |
| **Category** | **Phenotype** | **1/OR [95% CI]** | **Adjusted p-value** | **1/OR [95% CI]** | **Adjusted p-value** | **1/OR [95% CI]** | **Adjusted p-value** | **1/OR [95% CI]** | **Adjusted p-value** |
| Aids_Mobility | CD | 4 [3.7;4.55] | p < 1e-3 | 5.56 [5;6.25] | p < 1e-3 | 6.25 [5;7.69] | p < 1e-3 | 4.17 [2.94;6.25] | p < 1e-3 |
|  | RA | 2.86 [2.63;3.12] | p < 1e-3 | 4.17 [3.85;4.76] | p < 1e-3 | 5.88 [5;7.14] | p < 1e-3 | 4.17 [2.86;5.88] | p < 1e-3 |
|  | CTR | 5.56 [5;5.88] | p < 1e-3 | 7.69 [7.14;9.09] | p < 1e-3 | 10 [8.33;12.5] | p < 1e-3 | 5.88 [4;8.33] | p < 1e-3 |
| Incontinence | CD | 5.88 [5;6.67] | p < 1e-3 | 8.33 [7.14;10] | p < 1e-3 | 6.25 [5.26;7.69] | p < 1e-3 | 4.55 [3.33;6.25] | p < 1e-3 |
|  | RA | 11.11 [10;12.5] | p < 1e-3 | 11.11 [9.09;12.5] | p < 1e-3 | 9.09 [7.69;11.11] | p < 1e-3 | 5.88 [4.35;7.69] | p < 1e-3 |
|  | CTR | 14.29 [12.5;16.67] | p < 1e-3 | 12.5 [11.11;14.29] | p < 1e-3 | 11.11 [9.09;12.5] | p < 1e-3 | 6.25 [4.76;8.33] | p < 1e-3 |
| Nursing.items | CD | 4.17 [2.56;7.14] | p < 1e-3 | 5.56 [3.33;9.09] | p < 1e-3 | 3.57 [2.22;5.56] | p < 1e-3 | 3.12 [1.67;5.88] | p = 0.26 |
|  | RA | 6.67 [4.35;9.09] | p < 1e-3 | 5.88 [4.35;8.33] | p < 1e-3 | 4 [2.78;5.56] | p < 1e-3 | 2.22 [1.28;3.85] | p = 1 |
|  | CTR | 9.09 [6.25;14.29] | p < 1e-3 | 6.67 [4.76;10] | p < 1e-3 | 5.26 [3.45;7.69] | p < 1e-3 | 2.44 [1.39;4.35] | p = 1 |
| Visual | CD | 1.56 [1.19;2] | p = 0.565 | 2.22 [1.61;3.03] | p < 1e-3 | 1.61 [1.04;2.5] | p = 1 | 1.82 [1.12;2.94] | p = 1 |
|  | RA | 1.92 [1.54;2.38] | p < 1e-3 | 2.5 [2;3.23] | p < 1e-3 | 1.64 [1.15;2.38] | p = 1 | 1.79 [1.15;2.7] | p = 1 |
|  | CTR | 2.38 [1.92;2.94] | p < 1e-3 | 3.23 [2.44;4.17] | p < 1e-3 | 2.44 [1.67;3.57] | p = 0.004 | 2.38 [1.54;3.7] | p = 0.086 |
| Toilet | CD | 3.85 [3.03;5] | p < 1e-3 | 3.23 [2.56;4] | p < 1e-3 | 2.17 [1.72;2.7] | p < 1e-3 | 1.41 [1.03;1.92] | p = 1 |
|  | RA | 2.7 [2.27;3.12] | p < 1e-3 | 2.27 [1.96;2.7] | p < 1e-3 | 1.85 [1.54;2.22] | p < 1e-3 | 1.28 [0.96;1.69] | p = 1 |
|  | CTR | 5.88 [4.76;6.67] | p < 1e-3 | 5 [4.17;5.88] | p < 1e-3 | 3.23 [2.7;4] | p < 1e-3 | 1.79 [1.32;2.38] | p = 0.078 |
| Care.aids | CD | 14.29 [10;20] | p < 1e-3 | 12.5 [10;16.67] | p < 1e-3 | 7.69 [6.25;10] | p < 1e-3 | 5.56 [4.17;7.14] | p < 1e-3 |
|  | RA | 16.67 [14.29;20] | p < 1e-3 | 12.5 [11.11;14.29] | p < 1e-3 | 7.69 [6.67;9.09] | p < 1e-3 | 5 [4;6.67] | p < 1e-3 |
|  | CTR | 25 [20;25] | p < 1e-3 | 16.67 [14.29;20] | p < 1e-3 | 11.11 [9.09;12.5] | p < 1e-3 | 6.67 [5;8.33] | p < 1e-3 |
| Body.care_hygiene | CD | 14.29 [10;20] | p < 1e-3 | 11.11 [7.69;14.29] | p < 1e-3 | 5 [3.85;6.67] | p < 1e-3 | 2.33 [1.64;3.33] | p = 0.001 |
|  | RA | 20 [14.29;25] | p < 1e-3 | 11.11 [10;14.29] | p < 1e-3 | 5.88 [4.76;7.14] | p < 1e-3 | 2.27 [1.64;3.12] | p < 1e-3 |
|  | CTR | 25 [20;33.33] | p < 1e-3 | 20 [14.29;25] | p < 1e-3 | 7.69 [6.25;10] | p < 1e-3 | 2.56 [1.85;3.57] | p < 1e-3 |

### Table S9: The comparisons of MS, CD, RA, and CTR for each age group, with respect to each nursing care level.

|  |  | **20-29** | | **30-39** | | **40-49** | |
| --- | --- | --- | --- | --- | --- | --- | --- |
| **Category** | **Phenotype** | **1/OR [95% CI]** | **Adjusted p-value** | **1/OR [95% CI]** | **Adjusted p-value** | **1/OR [95% CI]** | **Adjusted p-value** |
| Level 1 | CD | 6.17 [1.16;33.33] | p = 1 | 14.71 [3.45;50] | p = 0.181 | 7.94 [3.85;16.67] | p < 1e-3 |
|  | RA | 7.04 [0.82;50] | p = 1 | 3.57 [1.56;8.33] | p = 1 | 4.55 [2.86;7.14] | p < 1e-3 |
|  | CTR | 23.26 [2.63;Inf] | p = 1 | 11.76 [5.26;25] | p < 1e-3 | 14.71 [9.09;25] | p < 1e-3 |
| Level 2 | CD | 3.4 [1.12;10] | p = 1 | 13.33 [6.25;25] | p < 1e-3 | 7.63 [5.26;11.11] | p < 1e-3 |
|  | RA | 0.93 [0.36;2.38] | p = 1 | 4.27 [2.56;7.14] | p < 1e-3 | 5.85 [4.35;7.69] | p < 1e-3 |
|  | CTR | 4.07 [1.52;11.11] | p = 1 | 8.13 [5.26;12.5] | p < 1e-3 | 17.54 [12.5;25] | p < 1e-3 |
| Level 3 | CD | 1.19 [0.22;6.67] | p = 1 | 2.72 [1.49;5] | p = 0.652 | 9.8 [5.88;16.67] | p < 1e-3 |
|  | RA | 1.35 [0.19;10] | p = 1 | 2.3 [1.25;4.17] | p = 1 | 6.25 [4.35;9.09] | p < 1e-3 |
|  | CTR | 0.65 [0.15;2.86] | p = 1 | 3.79 [2.27;6.25] | p < 1e-3 | 9.8 [7.14;12.5] | p < 1e-3 |
| Level 4 | CD | 1.38 [0.25;7.69] | p = 1 | 3.48 [1.49;8.33] | p = 1 | 12.35 [6.25;25] | p < 1e-3 |
|  | RA | 1.44 [0.2;10] | p = 1 | 3.41 [1.41;8.33] | p = 1 | 20.41 [11.11;33.33] | p < 1e-3 |
|  | CTR | 2.06 [0.4;10] | p = 1 | 5.18 [2.56;10] | p = 0.002 | 14.71 [10;20] | p < 1e-3 |
| Level 5 | CD | 0 [0;2.27] | p = 1 | 1.39 [0.31;6.25] | p = 1 | 18.52 [5.88;50] | p < 1e-3 |
|  | RA | 0 [0;6.25] | p = 1 | 2.22 [0.37;12.5] | p = 1 | 17.24 [7.14;50] | p < 1e-3 |
|  | CTR | 0 [0;3.33] | p = 1 | 1 [0.29;3.45] | p = 1 | 18.18 [10;33.33] | p < 1e-3 |

|  |  | **50-59** | | **60-69** | | **70-79** | | **80 - +** | |
| --- | --- | --- | --- | --- | --- | --- | --- | --- | --- |
| **Category** | **Phenotype** | **1/OR [95% CI]** | **Adjusted p-value** | **1/OR [95% CI]** | **Adjusted p-value** | **1/OR [95% CI]** | **Adjusted p-value** | **1/OR [95% CI]** | **Adjusted p-value** |
| Level 1 | CD | 4.22 [2.78;6.67] | p < 1e-3 | 5.49 [3.33;9.09] | p < 1e-3 | 4.37 [2.63;7.14] | p < 1e-3 | 2.22 [1.01;5] | p = 1 |
|  | RA | 4.74 [3.45;6.67] | p < 1e-3 | 4.61 [3.33;6.25] | p < 1e-3 | 4.18 [2.78;6.25] | p < 1e-3 | 2.07 [0.98;4.35] | p = 1 |
|  | CTR | 9.01 [6.25;12.5] | p < 1e-3 | 6.06 [4.17;9.09] | p < 1e-3 | 6.06 [3.85;9.09] | p < 1e-3 | 2.69 [1.25;5.88] | p = 1 |
| Level 2 | CD | 7.09 [5.56;9.09] | p < 1e-3 | 6.58 [5.26;8.33] | p < 1e-3 | 4.69 [3.57;6.25] | p < 1e-3 | 3.62 [2.33;5.56] | p < 1e-3 |
|  | RA | 6.85 [5.88;8.33] | p < 1e-3 | 7.69 [6.67;9.09] | p < 1e-3 | 5.62 [4.55;7.14] | p < 1e-3 | 3.3 [2.17;5] | p < 1e-3 |
|  | CTR | 10.87 [9.09;12.5] | p < 1e-3 | 12.35 [10;14.29] | p < 1e-3 | 8.77 [6.67;11.11] | p < 1e-3 | 4.29 [2.86;6.67] | p < 1e-3 |
| Level 3 | CD | 8.85 [6.67;12.5] | p < 1e-3 | 12.66 [10;16.67] | p < 1e-3 | 9.62 [7.14;12.5] | p < 1e-3 | 6.9 [4.76;10] | p < 1e-3 |
|  | RA | 11.76 [9.09;14.29] | p < 1e-3 | 12.2 [10;14.29] | p < 1e-3 | 11.49 [9.09;14.29] | p < 1e-3 | 7.09 [5;10] | p < 1e-3 |
|  | CTR | 15.15 [12.5;20] | p < 1e-3 | 15.38 [12.5;20] | p < 1e-3 | 16.95 [14.29;20] | p < 1e-3 | 9.52 [6.67;14.29] | p < 1e-3 |
| Level 4 | CD | 13.51 [9.09;20] | p < 1e-3 | 18.87 [14.29;25] | p < 1e-3 | 11.24 [8.33;16.67] | p < 1e-3 | 7.69 [5;11.11] | p < 1e-3 |
|  | RA | 22.22 [16.67;33.33] | p < 1e-3 | 21.28 [16.67;25] | p < 1e-3 | 13.16 [10;16.67] | p < 1e-3 | 7.87 [5.26;11.11] | p < 1e-3 |
|  | CTR | 17.86 [14.29;25] | p < 1e-3 | 24.39 [20;33.33] | p < 1e-3 | 16.95 [12.5;25] | p < 1e-3 | 9.35 [6.25;14.29] | p < 1e-3 |
| Level 5 | CD | 14.93 [8.33;25] | p < 1e-3 | 58.82 [25;100] | p < 1e-3 | 15.87 [10;25] | p < 1e-3 | 10.1 [6.25;16.67] | p < 1e-3 |
|  | RA | 22.73 [14.29;33.33] | p < 1e-3 | 35.71 [25;50] | p < 1e-3 | 22.22 [16.67;33.33] | p < 1e-3 | 12.66 [7.69;20] | p < 1e-3 |
|  | CTR | 25.64 [16.67;33.33] | p < 1e-3 | 31.25 [20;50] | p < 1e-3 | 16.95 [12.5;25] | p < 1e-3 | 13.16 [8.33;20] | p < 1e-3 |

### Table S10: Predictive performances obtained through 50 replications using the area under the receiver operating characteristic curve (AUROC), the area under the precision-recall curve (AUPR), F1, and Average Balanced Accuracy (Avg. Balanced Accr.)

| **Replication** | **AUROC** | **AUPR** | **F1** | **Avg. Balanced Accr.** |
| --- | --- | --- | --- | --- |
| 1 | 0.950 | 0.758 | 0.558 | 0.890 |
| 2 | 0.940 | 0.731 | 0.522 | 0.866 |
| 3 | 0.936 | 0.727 | 0.508 | 0.867 |
| 4 | 0.937 | 0.717 | 0.526 | 0.877 |
| 5 | 0.936 | 0.700 | 0.504 | 0.870 |
| 6 | 0.941 | 0.708 | 0.519 | 0.866 |
| 7 | 0.923 | 0.700 | 0.499 | 0.860 |
| 8 | 0.930 | 0.699 | 0.516 | 0.856 |
| 9 | 0.938 | 0.698 | 0.528 | 0.868 |
| 10 | 0.946 | 0.731 | 0.531 | 0.872 |
| 11 | 0.944 | 0.732 | 0.490 | 0.867 |
| 12 | 0.944 | 0.712 | 0.558 | 0.874 |
| 13 | 0.941 | 0.713 | 0.530 | 0.875 |
| 14 | 0.948 | 0.740 | 0.567 | 0.875 |
| 15 | 0.952 | 0.737 | 0.547 | 0.882 |
| 16 | 0.929 | 0.709 | 0.520 | 0.859 |
| 17 | 0.937 | 0.709 | 0.530 | 0.868 |
| 18 | 0.937 | 0.679 | 0.507 | 0.865 |
| 19 | 0.931 | 0.687 | 0.523 | 0.858 |
| 20 | 0.948 | 0.736 | 0.568 | 0.883 |
| 21 | 0.939 | 0.699 | 0.512 | 0.870 |
| 22 | 0.945 | 0.719 | 0.547 | 0.873 |
| 23 | 0.951 | 0.756 | 0.576 | 0.891 |
| 24 | 0.927 | 0.701 | 0.457 | 0.851 |
| 25 | 0.942 | 0.691 | 0.532 | 0.875 |
| 26 | 0.933 | 0.686 | 0.511 | 0.860 |
| 27 | 0.940 | 0.701 | 0.494 | 0.869 |
| 28 | 0.922 | 0.638 | 0.458 | 0.849 |
| 29 | 0.942 | 0.720 | 0.519 | 0.872 |
| 30 | 0.921 | 0.667 | 0.455 | 0.848 |
| 31 | 0.928 | 0.681 | 0.515 | 0.857 |
| 32 | 0.948 | 0.720 | 0.520 | 0.881 |
| 33 | 0.943 | 0.711 | 0.560 | 0.879 |
| 34 | 0.950 | 0.740 | 0.542 | 0.885 |
| 35 | 0.934 | 0.686 | 0.505 | 0.866 |
| 36 | 0.941 | 0.690 | 0.506 | 0.867 |
| 37 | 0.937 | 0.708 | 0.493 | 0.870 |
| 38 | 0.937 | 0.685 | 0.469 | 0.868 |
| 39 | 0.933 | 0.693 | 0.489 | 0.872 |
| 40 | 0.931 | 0.694 | 0.496 | 0.862 |
| 41 | 0.951 | 0.760 | 0.534 | 0.877 |
| 42 | 0.941 | 0.726 | 0.521 | 0.872 |
| 43 | 0.926 | 0.698 | 0.501 | 0.859 |
| 44 | 0.937 | 0.709 | 0.501 | 0.869 |
| 45 | 0.946 | 0.728 | 0.505 | 0.880 |
| 46 | 0.947 | 0.712 | 0.549 | 0.879 |
| 47 | 0.940 | 0.708 | 0.549 | 0.869 |
| 48 | 0.943 | 0.731 | 0.532 | 0.882 |
| 49 | 0.938 | 0.715 | 0.505 | 0.872 |
| 50 | 0.940 | 0.721 | 0.528 | 0.877 |

### Table S11: The mean absolute Shapley values that show the contributions of each feature to the disease progression prediction.

| **Feature** | **MeanAbsShap** |
| --- | --- |
| Order of Observation | 0.882 |
| Mobility Aids | 0.804 |
| Year of Observation | 0.769 |
| Age | 0.764 |
| MS Subtype (RRMS, SPMS, PPMS) | 0.308 |
| DMT LEVEL | 0.169 |
| Number of Doctor Visits | 0.149 |
| Sex | 0.111 |
| Physiotherapy, special, EB | 0.108 |
| Home Visits | 0.108 |
| Incontinence Aids | 0.074 |
| Residential Region | 0.055 |
| M54: Dorsalgia | 0.052 |
| N95: Menopausal and other perimenopausal disorders | 0.039 |
| Physiotherapy, normal, EB | 0.038 |
| F17: Nicotine dependence | 0.037 |
| M77: Other enthesopathies | 0.032 |
| M41: Scoliosis | 0.031 |
| E11: Type 2 diabetes mellitus | 0.028 |
| F06: Other mental disorders due to known physiological condition | 0.025 |
| I10: Essential (primary) hypertension | 0.024 |
| M47: Spondylosis | 0.024 |
| R52: Pain, unspecified | 0.023 |
| F41: Other anxiety disorders | 0.022 |
| J45: Asthma | 0.022 |
| E06: Thyroiditis | 0.021 |
| M42: Spinal osteochondrosis | 0.021 |
| M51: Thoracic, thoracolumbar, and lumbosacral intervertebral disc disorders | 0.020 |
| J06: Acute upper respiratory infections | 0.020 |
| M79: Other and unspecified soft tissue disorders | 0.020 |
| F48: Other nonpsychotic mental disorders | 0.019 |
| N89: Other noninflammatory disorders of vagina | 0.019 |
| H93: Other disorders of ear | 0.019 |
| D22: Melanocytic nevi | 0.017 |
| M19: Other and unspecified osteoarthritis | 0.016 |
| E03: Other hypothyroidism | 0.015 |
| H35: Other retinal disorders | 0.014 |
| J40: Bronchitis, not specified as acute or chronic | 0.014 |
| K44: Diaphragmatic hernia | 0.014 |
| Rehabilitation Duration | 0.014 |
| F43: Reaction to severe stress, and adjustment disorders | 0.013 |
| H50: Other strabismus | 0.013 |
| J44: Other chronic obstructive pulmonary disease | 0.013 |
| H52: Disorders of refraction and accommodation | 0.012 |
| R07: Pain in throat and chest | 0.012 |
| M48: Other spondylopathies | 0.012 |
| E55: Vitamin D deficiency | 0.012 |
| E79: Disorders of purine and pyrimidine metabolism | 0.011 |
| N94: Pain and other conditions associated with female genital organs and menstrual cycle | 0.011 |
| F45: Somatoform disorders | 0.011 |
| I34: Nonrheumatic mitral valve disorders | 0.011 |
| E87: Other disorders of fluid, electrolyte and acid-base balance | 0.011 |
| H90: Conductive and sensorineural hearing loss | 0.011 |
| M50: Cervical disc disorders | 0.010 |
| Individual treatment | 0.010 |
| M21: Other acquired deformities of limbs | 0.010 |
| E74: Other disorders of carbohydrate metabolism | 0.010 |
| C44: Other and unspecified malignant neoplasm of skin | 0.010 |
| I87: Other disorders of veins | 0.010 |
| F51: Sleep disorders not due to a substance or known physiological condition | 0.009 |
| J01: Acute sinusitis | 0.009 |
| M22: Disorder of patella | 0.008 |
| M16: Osteoarthritis of hip | 0.008 |
| R11: Nausea and vomiting | 0.008 |
| N92: Excessive, frequent and irregular menstruation | 0.008 |
| J34: Other and unspecified disorders of nose and nasal sinuses | 0.008 |
| K80: Cholelithiasis | 0.008 |
| M62: Other disorders of muscle | 0.007 |
| M81: Osteoporosis without current pathological fracture | 0.007 |
| F20: Schizophrenia | 0.007 |
| I48: Atrial fibrillation and flutter | 0.007 |
| J31: Chronic rhinitis, nasopharyngitis and pharyngitis | 0.007 |
| Q66: Congenital deformities of feet | 0.006 |
| M17: Osteoarthritis of knee | 0.006 |
| S83: Dislocation and sprain of joints and ligaments of knee | 0.006 |
| T14: Injury of unspecified body region | 0.006 |
| M25: Other joint disorder | 0.006 |
| N18: Chronic kidney disease (CKD) | 0.006 |
| E04: Other nontoxic goiter | 0.006 |
| K57: Diverticular disease of intestine | 0.005 |
| R10: Abdominal and pelvic pain | 0.005 |
| K29: Gastritis and duodenitis | 0.005 |
| E10: Type 1 diabetes mellitus | 0.005 |
| R41: Other symp.* involving cognitive functions and awareness | 0.005 |
| I83: Varicose veins of lower extremities | 0.005 |
| J30: Vasomotor and allergic rhinitis | 0.005 |
| R15: Fecal incontinence | 0.005 |
| Findings | 0.005 |
| K30: Functional dyspepsia | 0.005 |
| Podological therapy measures | 0.005 |
| H18: Other disorders of cornea | 0.005 |
| M95: Other acquired deformities of musculoskeletal system and connective tissue | 0.005 |
| N28: Other disorders of kidney and ureter | 0.005 |
| T78: Adverse effects | 0.004 |
| F02: Dementia in other diseases classified elsewhere | 0.004 |
| K76: Other diseases of liver | 0.004 |
| Massages | 0.004 |
| D64: Other anemias | 0.004 |
| N86: Erosion and ectropion of cervix uteri | 0.004 |
| M94: Other disorders of cartilage | 0.004 |
| F07: Personality and behavioral disorders due to known physiological condition | 0.004 |
| F60: Specific personality disorders | 0.004 |
| R14: Flatulence and related conditions | 0.004 |
| L30: Other and unspecified dermatitis | 0.004 |
| D12: Benign neoplasm of colon, rectum, anus and anal canal | 0.004 |
| E66: Overweight and obesity | 0.004 |
| E78: Disorders of lipoprotein metabolism and other lipidemias | 0.004 |
| J32: Chronic sinusitis | 0.004 |
| K59: Other functional intestinal disorders | 0.004 |
| H49: Paralytic strabismus | 0.004 |
| H36: Retinal disorders in diseases classified elsewhere | 0.003 |
| D35: Benign neoplasm of other and unspecified endocrine glands | 0.003 |
| M13: Other arthritis | 0.003 |
| M99: Biomechanical lesions | 0.003 |
| L71: Rosacea | 0.003 |
| C50: Malignant neoplasm of breast | 0.003 |
| J42: Unspecified chronic bronchitis | 0.003 |
| D25: Leiomyoma of uterus | 0.003 |
| B35: Dermatophytosis | 0.003 |
| K58: Irritable bowel syndrome | 0.003 |
| Care aids to facilitate care | 0.003 |
| M96: Intraoperative and postprocedural complications and disorders of musculoskeletal system | 0.003 |
| F13: Sedative, hypnotic, or anxiolytic related disorders | 0.003 |
| F44: Dissociative and conversion disorders | 0.003 |
| M43: Other deforming dorsopathies | 0.003 |
| M20: Acquired deformities of fingers and toes | 0.003 |
| J98: Other respiratory disorders | 0.003 |
| Q74: Other congenital malformations of limb(s) | 0.003 |
| S42: Fracture of shoulder and upper arm | 0.003 |
| R60: Edema | 0.003 |
| F03: Unspecified dementia | 0.002 |
| M67: Other disorders of synovium and tendon | 0.002 |
| Manual therapy | 0.002 |
| J41: Simple and mucopurulent chronic bronchitis | 0.002 |
| M65: Synovitis and tenosynovitis | 0.002 |
| I35: Nonrheumatic aortic valve disorders | 0.002 |
| M34: Systemic sclerosis [scleroderma] | 0.002 |
| R42: Dizziness and giddiness | 0.002 |
| L89: Pressure ulcer | 0.002 |
| I89: Other noninfective disorders of lymphatic vessels and lymph nodes | 0.002 |
| Care aids for body care/hygiene and for alleviating complaints | 0.002 |
| K22: Other diseases of esophagus | 0.002 |
| S01: Open wound of head | 0.002 |
| M53: Other and unspecified dorsopathies | 0.002 |
| D29: Benign neoplasm of male genital organs | 0.002 |
| N08: Glomerular disorders in diseases classified elsewhere | 0.002 |
| R53: Malaise and fatigue | 0.002 |
| I70: Atherosclerosis | 0.002 |
| D17: Benign lipomatous neoplasm | 0.002 |
| H02: Other disorders of eyelid | 0.002 |
| E14 | 0.001 |
| J20: Acute bronchitis | 0.001 |
| C77: Secondary and unspecified malignant neoplasm of lymph nodes | 0.001 |
| H26: Other cataract | 0.001 |
| H51: Other disorders of binocular movement | 0.001 |
| E89: Postprocedural endocrine and metabolic complications and disorders | 0.001 |
| F01: Vascular dementia | 0.001 |
| E53: Deficiency of other B group vitamins | 0.001 |
| D37: Neoplasm of uncertain behavior of oral cavity and digestive organs | 0.001 |
| L80: Vitiligo | 0.001 |
| S93: Dislocation and sprain of joints and ligaments at ankle, foot and toe level | 0.001 |
| H92: Otalgia and effusion of ear | 0.001 |
| H66: Suppurative and unspecified otitis media | 0.001 |
| N13: Obstructive and reflux uropathy | 0.001 |
| T83: Complications of genitourinary prosthetic devices, implants and grafts | 0.001 |
| C61: Malignant neoplasm of prostate | 0.001 |
| K71: Toxic liver disease | 0.001 |
| S62: Fracture at wrist and hand level | 0.001 |
| F10: Alcohol related disorders | 0.001 |
| Toilet Aids | 0.001 |
| D70: Neutropenia | 0.001 |
| K26: Duodenal ulcer | 0.001 |
| F00 | 0.001 |
| J90: Pleural effusion | 0.001 |
| I50: Heart failure | 0.001 |
| K21: Gastro-esophageal reflux disease | 0.001 |
| J18: Pneumonia, unspecified organism | 0.001 |
| D80: Immunodeficiency with predominantly antibody defects | 0.001 |
| J96: Respiratory failure | 0.001 |
| H20: Iridocyclitis | 0.001 |
| H25: Age-related cataract | 0.001 |
| I45: Other conduction disorders | 0.001 |
| L50: Urticaria | 0.001 |
| C64: Malignant neoplasm of kidney, except renal pelvis | 0.001 |
| Q65: Congenital deformities of hip | 0.000 |
| H11: Other disorders of conjunctiva | 0.000 |
| E86: Volume depletion | 0.000 |
| Man. Lymphatic drainage | 0.000 |
| D45: Polycythemia vera | 0.000 |
| J11: Influenza due to unidentified influenza virus | 0.000 |
| H10: Conjunctivitis | 0.000 |
| R80: Proteinuria | 0.000 |
| M75: Shoulder lesions | 0.000 |
| N91: Absent, scanty and rare menstruation | 0.000 |
| M40: Kyphosis and lordosis | 0.000 |
| K83: Other diseases of biliary tract | 0.000 |
| M35: Other systemic involvement of connective tissue | 0.000 |
| F52: Sexual dysfunction not due to a substance or known physiological condition | 0.000 |
| Heat and cold therapy | 0.000 |
| K31: Other diseases of stomach and duodenum | 0.000 |
| M23: Internal derangement of knee | 0.000 |
| I25: Chronic ischemic heart disease | 0.000 |
| E05: Thyrotoxicosis [hyperthyroidism] | 0.000 |
| S52: Fracture of forearm | 0.000 |
| D34: Benign neoplasm of thyroid gland | 0.000 |
| D50: Iron deficiency anemia | 0.000 |
| Rehabilitation Frequency | 0.000 |
| I73: Other peripheral vascular diseases | 0.000 |
| J04: Acute laryngitis and tracheitis | 0.000 |
| R51: Headache | 0.000 |
| C81: Hodgkin lymphoma | 0.000 |
| M82 | 0.000 |
| C78: Secondary malignant neoplasm of respiratory and digestive organs | 0.000 |
| Aids for the blind | 0.000 |
| Nursing Items | 0.000 |
| Visual Aids | 0.000 |
| Movement therapy/exercise treatment, EB | 0.000 |
| Movement therapy/Ü- x000D exercise treatment GB | 0.000 |
| Physiotherapy, normal GB | 0.000 |
| Physiotherapy in the exercise pool, EB | 0.000 |
| Physiotherapy in the x000D exercise pool, GB | 0.000 |
| Traction treatment/extension treatment | 0.000 |
| Electrotherapy | 0.000 |
| Medical baths | 0.000 |
| Inhalation therapy | 0.000 |
| Miscellaneous | 0.000 |
| Standardized combination of remedies | 0.000 |
| Initial examination | 0.000 |
| Individual treatment (price including preparation and follow-up | 0.000 |
| Group treatment (price including preparation and follow-up) | 0.000 |
| Thermal application, heat/cold | 0.000 |
| rails | 0.000 |
| Movement therapy, EB | 0.000 |
| Movement therapy, UK | 0.000 |
| Heat and cold therapy | 0.000 |
| Medical baths | 0.000 |
| J00: Acute nasopharyngitis [common cold] | 0.000 |
| F40: Phobic anxiety disorders | 0.000 |
| M60: Myositis | 0.000 |
| N80: Endometriosis | 0.000 |
| T08 | 0.000 |
| M00: Pyogenic arthritis | 0.000 |
| D10: Benign neoplasm of mouth and pharynx | 0.000 |
| A00: Cholera | 0.000 |
| R40: Somnolence, stupor and coma | 0.000 |
| L20: Atopic dermatitis | 0.000 |
| E70: Disorders of aromatic amino-acid metabolism | 0.000 |
| K20: Esophagitis | 0.000 |
| R50: Fever of other and unknown origin | 0.000 |
| K55: Vascular disorders of intestine | 0.000 |
| K50: Crohn's disease [regional enteritis] | 0.000 |
| N70: Salpingitis and oophoritis | 0.000 |
| E65: Localized adiposity | 0.000 |
| R00: Abnormalities of heart beat | 0.000 |
| E50: Vitamin A deficiency | 0.000 |
| H60: Otitis externa | 0.000 |
| T66: Radiation sickness, unspecified | 0.000 |
| E00: Congenital iodine-deficiency syndrome | 0.000 |
| I80: Phlebitis and thrombophlebitis | 0.000 |
| B99: Other and unspecified infectious diseases | 0.000 |
| K70: Alcoholic liver disease | 0.000 |
| N60: Benign mammary dysplasia | 0.000 |
| B25: Cytomegaloviral disease | 0.000 |
| H00: Hordeolum and chalazion | 0.000 |
| T80: Complications following infusion, transfusion and therapeutic injection | 0.000 |
| S90: Superficial injury of ankle, foot and toes | 0.000 |
| I95: Hypotension | 0.000 |
| H30: Chorioretinal inflammation | 0.000 |
| B00: Herpesviral [herpes simplex] infections | 0.000 |
| T79: Certain early complications of trauma | 0.000 |
| J95: Intraoperative and postprocedural complications and disorders of respiratory system | 0.000 |
| L60: Nail disorders | 0.000 |
| I30: Acute pericarditis | 0.000 |
| M80: Osteoporosis with current pathological fracture | 0.000 |
| D60: Acquired pure red cell aplasia [erythroblastopenia] | 0.000 |
| H65: Nonsuppurative otitis media | 0.000 |
| J09: Influenza due to certain identified influenza viruses | 0.000 |
| S80: Superficial injury of knee and lower leg | 0.000 |
| S00: Superficial injury of head | 0.000 |
| S20: Superficial injury of thorax | 0.000 |
| F50: Eating disorders | 0.000 |
| H40: Glaucoma | 0.000 |
| S60: Superficial injury of wrist, hand and fingers | 0.000 |
| K40: Inguinal hernia | 0.000 |
| N40: Benign prostatic hyperplasia | 0.000 |
| H43: Disorders of vitreous body | 0.000 |
| K90: Intestinal malabsorption | 0.000 |
| R70: Elevated erythrocyte sedimentation rate and abnormality of plasma viscosity | 0.000 |
| H55: Nystagmus and other irregular eye movements | 0.000 |
| N17: Acute kidney failure | 0.000 |
| E20: Hypoparathyroidism | 0.000 |
| S30: Superficial injury of abdomen, lower back, pelvis and external genitals | 0.000 |
| S10: Superficial injury of neck | 0.000 |
| M30: Polyarteritis nodosa and related conditions | 0.000 |
| O20: Hemorrhage in early pregnancy | 0.000 |
| I20: Angina pectoris | 0.000 |
| N25: Disorders resulting from impaired renal tubular function | 0.000 |
| R83: Abnormal findings in cerebrospinal fluid | 0.000 |
| L40: Psoriasis | 0.000 |
| H15: Disorders of sclera | 0.000 |
| T00 | 0.000 |
| D65: Disseminated intravascular coagulation [defibrination syndrome] | 0.000 |
| N10: Acute pyelonephritis | 0.000 |
| B95: Streptococcus, Staphylococcus, and Enterococcus as the cause of diseases classified elsewhere | 0.000 |
| N20: Calculus of kidney and ureter | 0.000 |
| K00: Disorders of tooth development and eruption | 0.000 |
| S40: Superficial injury of shoulder and upper arm | 0.000 |
| O80: Encounter for full-term uncomplicated delivery | 0.000 |
| C00: Malignant neoplasm of lip | 0.000 |
| S50: Superficial injury of elbow and forearm | 0.000 |
| T51: Toxic effect of alcohol | 0.000 |
| S70: Superficial injury of hip and thigh | 0.000 |
| T20: Burn and corrosion of head, face, and neck | 0.000 |
| L55: Sunburn | 0.000 |
| O30: Multiple gestation | 0.000 |
| F99: Mental disorder, not otherwise specified | 0.000 |
| O85: Puerperal sepsis | 0.000 |
| T15: Foreign body on external eye | 0.000 |
| O60: Preterm labor | 0.000 |
| K65: Peritonitis | 0.000 |
| Q80: Congenital ichthyosis | 0.000 |
| Q60: Renal agenesis and other reduction defects of kidney | 0.000 |
| N00: Acute nephritic syndrome | 0.000 |
| I05: Rheumatic mitral valve diseases | 0.000 |
| I26: Pulmonary embolism | 0.000 |
| O10: Pre-existing hypertension complicating pregnancy, childbirth and the puerperium | 0.000 |
| O00: Ectopic pregnancy | 0.000 |
| D00: Carcinoma in situ of oral cavity, esophagus and stomach | 0.000 |
| K35: Acute appendicitis | 0.000 |
| F90: Attention-deficit hyperactivity disorders | 0.000 |
| N99: Intraoperative and postprocedural complications and disorders of genitourinary system | 0.000 |
| Q10: Congenital malformations of eyelid, lacrimal apparatus and orbit | 0.000 |
| B85: Pediculosis and phthiriasis | 0.000 |
| M61: Calcification and ossification of muscle | 0.000 |
| N81: Female genital prolapse | 0.000 |
| T09 | 0.000 |
| M01: Direct infections of joint in infectious and parasitic diseases classified elsewhere | 0.000 |
| I11: Hypertensive heart disease | 0.000 |
| D11: Benign neoplasm of major salivary glands | 0.000 |
| A01: Typhoid and paratyphoid fevers | 0.000 |
| J21: Acute bronchiolitis | 0.000 |
| L21: Seborrheic dermatitis | 0.000 |
| E71: Disorders of branched-chain amino-acid metabolism and fatty-acid metabolism | 0.000 |
| K56: Paralytic ileus and intestinal obstruction without hernia | 0.000 |
| K51: Ulcerative colitis | 0.000 |
| N71: Inflammatory disease of uterus, except cervix | 0.000 |
| R01: Cardiac murmurs and other cardiac sounds | 0.000 |
| E51: Thiamine deficiency | 0.000 |
| H61: Other disorders of external ear | 0.000 |
| T67: Effects of heat and light | 0.000 |
| F11: Opioid related disorders | 0.000 |
| E01: Iodine-deficiency related thyroid disorders and allied conditions | 0.000 |
| B36: Other superficial mycoses | 0.000 |
| I81: Portal vein thrombosis | 0.000 |
| N61: Inflammatory disorders of breast | 0.000 |
| B26: Mumps | 0.000 |
| H01: Other inflammation of eyelid | 0.000 |
| H91: Other and unspecified hearing loss | 0.000 |
| T81: Complications of procedures | 0.000 |
| S91: Open wound of ankle, foot and toes | 0.000 |
| D51: Vitamin B12 deficiency anemia | 0.000 |
| H31: Other disorders of choroid | 0.000 |
| B01: Varicella [chickenpox] | 0.000 |
| L01: Impetigo | 0.000 |
| I31: Other diseases of pericardium | 0.000 |
| D38: Neoplasm of uncertain behavior of middle ear and respiratory and intrathoracic organs | 0.000 |
| D61: Other aplastic anemias and other bone marrow failure syndromes | 0.000 |
| J10: Influenza due to other identified influenza virus | 0.000 |
| K81: Cholecystitis | 0.000 |
| L81: Other disorders of pigmentation | 0.000 |
| S81: Open wound of knee and lower leg | 0.000 |
| S21: Open wound of thorax | 0.000 |
| S61: Open wound of wrist, hand and fingers | 0.000 |
| K41: Femoral hernia | 0.000 |
| N41: Inflammatory diseases of prostate | 0.000 |
| H44: Disorders of globe | 0.000 |
| K91: Intraoperative and postprocedural complications and disorders of digestive system | 0.000 |
| R71: Abnormality of red blood cells | 0.000 |
| A31: Infection due to other mycobacteria | 0.000 |
| I71: Aortic aneurysm and dissection | 0.000 |
| E21: Hyperparathyroidism and other disorders of parathyroid gland | 0.000 |
| L51: Erythema multiforme | 0.000 |
| S31: Open wound of abdomen, lower back, pelvis and external genitals | 0.000 |
| S11: Open wound of neck | 0.000 |
| M31: Other necrotizing vasculopathies | 0.000 |
| O21: Excessive vomiting in pregnancy | 0.000 |
| I21: Acute myocardial infarction | 0.000 |
| N26: Unspecified contracted kidney | 0.000 |
| R84: Abnormal findings in specimens from respiratory organs and thorax | 0.000 |
| L41: Parapsoriasis | 0.000 |
| H16: Keratitis | 0.000 |
| F61 | 0.000 |
| T01 | 0.000 |
| D66: Hereditary factor VIII deficiency | 0.000 |
| N11: Chronic tubulo-interstitial nephritis | 0.000 |
| B96: Other bacterial agents as the cause of diseases classified elsewhere | 0.000 |
| N21: Calculus of lower urinary tract | 0.000 |
| K01: Embedded and impacted teeth | 0.000 |
| S41: Open wound of shoulder and upper arm | 0.000 |
| R81: Glycosuria | 0.000 |
| O81 | 0.000 |
| S51: Open wound of elbow and forearm | 0.000 |
| S71: Open wound of hip and thigh | 0.000 |
| T21: Burn and corrosion of trunk | 0.000 |
| L56: Other acute skin changes due to ultraviolet radiation | 0.000 |
| O86: Other puerperal infections | 0.000 |
| T16: Foreign body in ear | 0.000 |
| O61: Failed induction of labor | 0.000 |
| K66: Other disorders of peritoneum | 0.000 |
| Q81: Epidermolysis bullosa | 0.000 |
| Q61: Cystic kidney disease | 0.000 |
| J61: Pneumoconiosis due to asbestos and other mineral fibers | 0.000 |
| F21: Schizotypal disorder | 0.000 |
| I06: Rheumatic aortic valve diseases | 0.000 |
| I27: Other pulmonary heart diseases | 0.000 |
| D81: Combined immunodeficiencies | 0.000 |
| O11: Pre-existing hypertension with pre-eclampsia | 0.000 |
| O01: Hydatidiform mole | 0.000 |
| A51: Early syphilis | 0.000 |
| E16: Other disorders of pancreatic internal secretion | 0.000 |
| D01: Carcinoma in situ of other and unspecified digestive organs | 0.000 |
| K36: Other appendicitis | 0.000 |
| F91: Conduct disorders | 0.000 |
| E41: Nutritional marasmus | 0.000 |
| Q11: Anophthalmos, microphthalmos and macrophthalmos | 0.000 |
| B86: Scabies | 0.000 |
| J02: Acute pharyngitis | 0.000 |
| R12: Heartburn | 0.000 |
| F42: Obsessive-compulsive disorder | 0.000 |
| T10 | 0.000 |
| M02: Postinfective and reactive arthropathies | 0.000 |
| I12: Hypertensive chronic kidney disease | 0.000 |
| A02: Other salmonella infections | 0.000 |
| J22: Unspecified acute lower respiratory infection | 0.000 |
| L22: Diaper dermatitis | 0.000 |
| E72: Other disorders of amino-acid metabolism | 0.000 |
| Q67: Congenital musculoskeletal deformities of head, face, spine and chest | 0.000 |
| K52: Other and unspecified noninfective gastroenteritis and colitis | 0.000 |
| N72: Inflammatory disease of cervix uteri | 0.000 |
| E67: Other hyperalimentation | 0.000 |
| R02 | 0.000 |
| H62: Disorders of external ear in diseases classified elsewhere | 0.000 |
| T68: Hypothermia | 0.000 |
| F12: Cannabis related disorders | 0.000 |
| E02: Subclinical iodine-deficiency hypothyroidism | 0.000 |
| B37: Candidiasis | 0.000 |
| I82: Other venous embolism and thrombosis | 0.000 |
| K72: Hepatic failure | 0.000 |
| N62: Hypertrophy of breast | 0.000 |
| B27: Infectious mononucleosis | 0.000 |
| T82: Complications of cardiac and vascular prosthetic devices, implants and grafts | 0.000 |
| S92: Fracture of foot and toe, except ankle | 0.000 |
| I97: Intraoperative and postprocedural complications and disorders of circulatory system | 0.000 |
| D52: Folate deficiency anemia | 0.000 |
| H32: Chorioretinal disorders in diseases classified elsewhere | 0.000 |
| B02: Zoster [herpes zoster] | 0.000 |
| L02: Cutaneous abscess, furuncle and carbuncle | 0.000 |
| H27: Other disorders of lens | 0.000 |
| E12 | 0.000 |
| D39: Neoplasm of uncertain behavior of female genital organs | 0.000 |
| D62: Acute posthemorrhagic anemia | 0.000 |
| H67: Otitis media in diseases classified elsewhere | 0.000 |
| K82: Other diseases of gallbladder | 0.000 |
| L82: Seborrheic keratosis | 0.000 |
| S82: Fracture of lower leg, including ankle | 0.000 |
| S02: Fracture of skull and facial bones | 0.000 |
| S22: Fracture of rib(s), sternum and thoracic spine | 0.000 |
| H42: Glaucoma in diseases classified elsewhere | 0.000 |
| K42: Umbilical hernia | 0.000 |
| N42: Other and unspecified disorders of prostate | 0.000 |
| K92: Other diseases of digestive system | 0.000 |
| R72 | 0.000 |
| A32: Listeriosis | 0.000 |
| H57: Other disorders of eye and adnexa | 0.000 |
| N19: Unspecified kidney failure | 0.000 |
| I72: Other aneurysm | 0.000 |
| E22: Hyperfunction of pituitary gland | 0.000 |
| L52: Erythema nodosum | 0.000 |
| S32: Fracture of lumbar spine and pelvis | 0.000 |
| S12: Fracture of cervical vertebra and other parts of neck | 0.000 |
| M32: Systemic lupus erythematosus (SLE) | 0.000 |
| O22: Venous complications and hemorrhoids in pregnancy | 0.000 |
| I22: Subsequent ST elevation (STEMI) and non-ST elevation (NSTEMI) myocardial infarction | 0.000 |
| D72: Other disorders of white blood cells | 0.000 |
| N27: Small kidney of unknown cause | 0.000 |
| R85: Abnormal findings in specimens from digestive organs and abdominal cavity | 0.000 |
| L42: Pityriasis rosea | 0.000 |
| H17: Corneal scars and opacities | 0.000 |
| F62 | 0.000 |
| T02 | 0.000 |
| D67: Hereditary factor IX deficiency | 0.000 |
| N12: Tubulo-interstitial nephritis, not specified as acute or chronic | 0.000 |
| B97: Viral agents as the cause of diseases classified elsewhere | 0.000 |
| K02: Dental caries | 0.000 |
| R82: Other and unspecified abnormal findings in urine | 0.000 |
| O82: Encounter for cesarean delivery without indication | 0.000 |
| S72: Fracture of femur | 0.000 |
| T22: Burn and corrosion of shoulder and upper limb, except wrist and hand | 0.000 |
| L57: Skin changes due to chronic exposure to nonionizing radiation | 0.000 |
| O32: Maternal care for malpresentation of fetus | 0.000 |
| O87: Venous complications and hemorrhoids in the puerperium | 0.000 |
| T17: Foreign body in respiratory tract | 0.000 |
| O62: Abnormalities of forces of labor | 0.000 |
| Q82: Other congenital malformations of skin | 0.000 |
| Q62: Congenital obstructive defects of renal pelvis and congenital malformations of ureter | 0.000 |
| N02: Recurrent and persistent hematuria | 0.000 |
| F22: Delusional disorders | 0.000 |
| I07: Rheumatic tricuspid valve diseases | 0.000 |
| I28: Other diseases of pulmonary vessels | 0.000 |
| D82: Immunodeficiency associated with other major defects | 0.000 |
| O12: Gestational [pregnancy-induced] edema and proteinuria without hypertension | 0.000 |
| O02: Other abnormal products of conception | 0.000 |
| A52: Late syphilis | 0.000 |
| D02: Carcinoma in situ of middle ear and respiratory system | 0.000 |
| K37: Unspecified appendicitis | 0.000 |
| F92 | 0.000 |
| Q12: Congenital lens malformations | 0.000 |
| B87: Myiasis | 0.000 |
| J03: Acute tonsillitis | 0.000 |
| R13: Aphagia and dysphagia | 0.000 |
| M63: Disorders of muscle in diseases classified elsewhere | 0.000 |
| N83: Noninf. dis.* of ovary, fallopian tube and broad ligament | 0.000 |
| T11 | 0.000 |
| I13: Hypertensive heart and chronic kidney disease | 0.000 |
| D13: Benign neoplasm of other and ill-defined parts of digestive system | 0.000 |
| R43: Disturbances of smell and taste | 0.000 |
| L23: Allergic contact dermatitis | 0.000 |
| E73: Lactose intolerance | 0.000 |
| J43: Emphysema | 0.000 |
| J33: Nasal polyp | 0.000 |
| H13 | 0.000 |
| Q68: Other congenital musculoskeletal deformities | 0.000 |
| N73: Other female pelvic inflammatory diseases | 0.000 |
| E68: Sequelae of hyperalimentation | 0.000 |
| R03: Abnormal blood-pressure reading, without diagnosis | 0.000 |
| T69: Other effects of reduced temperature | 0.000 |
| K73: Chronic hepatitis | 0.000 |
| N63: Unspecified lump in breast | 0.000 |
| H03 | 0.000 |
| I98 | 0.000 |
| D53: Other nutritional anemias | 0.000 |
| H33: Retinal detachments and breaks | 0.000 |
| L03: Cellulitis and acute lymphangitis | 0.000 |
| H28: Cataract in diseases classified elsewhere | 0.000 |
| L63: Alopecia areata | 0.000 |
| I33: Acute and subacute endocarditis | 0.000 |
| E13: Other specified diabetes mellitus | 0.000 |
| D40: Neoplasm of uncertain behavior of male genital organs | 0.000 |
| M83: Adult osteomalacia | 0.000 |
| D63: Anemia in chronic diseases classified elsewhere | 0.000 |
| H68: Eustachian salpingitis and obstruction | 0.000 |
| J12: Viral pneumonia | 0.000 |
| L83: Acanthosis nigricans | 0.000 |
| S03: Dislocation and sprain of joints and ligaments of head | 0.000 |
| S23: Dislocation and sprain of joints and ligaments of thorax | 0.000 |
| F53: Mental and behavioral disorders associated with the puerperium | 0.000 |
| S63: Dislocation and sprain of joints and ligaments at wrist and hand level | 0.000 |
| K43: Ventral hernia | 0.000 |
| N43: Hydrocele and spermatocele | 0.000 |
| K93 | 0.000 |
| R73: Elevated blood glucose level | 0.000 |
| H58 | 0.000 |
| E23: Hypofunction and other disorders of the pituitary gland | 0.000 |
| L53: Other erythematous conditions | 0.000 |
| S33: Dislocation and sprain of joints and ligaments of lumbar spine and pelvis | 0.000 |
| S13: Dislocation and sprain of joints and ligaments at neck level | 0.000 |
| M33: Dermatopolymyositis | 0.000 |
| O23: Infections of genitourinary tract in pregnancy | 0.000 |
| D73: Diseases of spleen | 0.000 |
| R86: Abnormal findings in specimens from male genital organs | 0.000 |
| L43: Lichen planus | 0.000 |
| F63: Impulse disorders | 0.000 |
| T03 | 0.000 |
| D68: Other coagulation defects | 0.000 |
| B98 | 0.000 |
| N23: Unspecified renal colic | 0.000 |
| K03: Other diseases of hard tissues of teeth | 0.000 |
| S43: Dislocation and sprain of joints and ligaments of shoulder girdle | 0.000 |
| S53: Dislocation and sprain of joints and ligaments of elbow | 0.000 |
| A68: Relapsing fevers | 0.000 |
| S73: Dislocation and sprain of joint and ligaments of hip | 0.000 |
| T23: Burn and corrosion of wrist and hand | 0.000 |
| L58: Radiodermatitis | 0.000 |
| O33: Maternal care for disproportion | 0.000 |
| O88: Obstetric embolism | 0.000 |
| T18: Foreign body in alimentary tract | 0.000 |
| O63: Long labor | 0.000 |
| Q83: Congenital malformations of breast | 0.000 |
| Q63: Other congenital malformations of kidney | 0.000 |
| J93: Pneumothorax and air leak | 0.000 |
| N03: Chronic nephritic syndrome | 0.000 |
| F23: Brief psychotic disorder | 0.000 |
| I08: Multiple valve diseases | 0.000 |
| D83: Common variable immunodeficiency | 0.000 |
| O13: Gestational [pregnancy-induced] hypertension without significant proteinuria | 0.000 |
| O03: Spontaneous abortion | 0.000 |
| A53: Other and unspecified syphilis | 0.000 |
| D03: Melanoma in situ | 0.000 |
| K38: Other diseases of appendix | 0.000 |
| F93: Emotional disorders with onset specific to childhood | 0.000 |
| A23: Brucellosis | 0.000 |
| E43: Unspecified severe protein-calorie malnutrition | 0.000 |
| Q13: Congenital malformations of anterior segment of eye | 0.000 |
| B88: Other infestations | 0.000 |
| N84: Polyp of female genital tract | 0.000 |
| T12 | 0.000 |
| D14: Benign neoplasm of middle ear and respiratory system | 0.000 |
| A04: Other bacterial intestinal infections | 0.000 |
| R44: Other symp.* involving general sensations and perceptions | 0.000 |
| L24: Irritant contact dermatitis | 0.000 |
| R54: Age-related physical debility | 0.000 |
| N74: Female pelvic inflammatory disorders in diseases classified elsewhere | 0.000 |
| R04: Hemorrhage from respiratory passages | 0.000 |
| E54: Ascorbic acid deficiency | 0.000 |
| T70: Effects of air pressure and water pressure | 0.000 |
| F14: Cocaine related disorders | 0.000 |
| I84 | 0.000 |
| K74: Fibrosis and cirrhosis of liver | 0.000 |
| N64: Other disorders of breast | 0.000 |
| F04: Amnestic disorder due to known physiological condition | 0.000 |
| H04: Disorders of lacrimal system | 0.000 |
| H94: Other disorders of ear in diseases classified elsewhere | 0.000 |
| T84: Complications of internal orthopedic prosthetic devices, implants and grafts | 0.000 |
| S94: Injury of nerves at ankle and foot level | 0.000 |
| I99: Other and unspecified disorders of circulatory system | 0.000 |
| H34: Retinal vascular occlusions | 0.000 |
| L04: Acute lymphadenitis | 0.000 |
| J99: Respiratory disorders in diseases classified elsewhere | 0.000 |
| L64: Androgenic alopecia | 0.000 |
| D41: Neoplasm of uncertain behavior of urinary organs | 0.000 |
| M84: Disorder of continuity of bone | 0.000 |
| H69: Other and unspecified disorders of Eustachian tube | 0.000 |
| J13: Pneumonia due to Streptococcus pneumoniae | 0.000 |
| L84: Corns and callosities | 0.000 |
| S84: Injury of nerves at lower leg level | 0.000 |
| S04: Injury of cranial nerve | 0.000 |
| S24: Injury of nerves and spinal cord at thorax level | 0.000 |
| F54: Psychological and behavioral factors associated with disorders or diseases classified elsewhere | 0.000 |
| S64: Injury of nerves at wrist and hand level | 0.000 |
| N44: Noninf. dis.* of testis | 0.000 |
| R74: Abnormal serum enzyme levels | 0.000 |
| H59: Intraoperative and postprocedural complications and disorders of eye and adnexa | 0.000 |
| I74: Arterial embolism and thrombosis | 0.000 |
| E24: Cushing's syndrome | 0.000 |
| O98: Maternal infectious and parasitic diseases classifiable elsewhere but complicating pregnancy, childbirth and the puerperium | 0.000 |
| S34: Injury of lumbar and sacral spinal cord and nerves at abdomen, lower back and pelvis level | 0.000 |
| S14: Injury of nerves and spinal cord at neck level | 0.000 |
| O24: Diabetes mellitus in pregnancy, childbirth, and the puerperium | 0.000 |
| I24: Other acute ischemic heart diseases | 0.000 |
| N29: Other disorders of kidney and ureter in diseases classified elsewhere | 0.000 |
| R87: Abnormal findings in specimens from female genital organs | 0.000 |
| L44: Other papulosquamous disorders | 0.000 |
| H19 | 0.000 |
| F64: Gender identity disorders | 0.000 |
| T04 | 0.000 |
| D69: Purpura and other hemorrhagic conditions | 0.000 |
| K04: Diseases of pulp and periapical tissues | 0.000 |
| S44: Injury of nerves at shoulder and upper arm level | 0.000 |
| S54: Injury of nerves at forearm level | 0.000 |
| A69: Other spirochetal infections | 0.000 |
| S74: Injury of nerves at hip and thigh level | 0.000 |
| T24: Burn and corrosion of lower limb, except ankle and foot | 0.000 |
| L59: Other disorders of skin and subcutaneous tissue related to radiation | 0.000 |
| O34: Maternal care for abnormality of pelvic organs | 0.000 |
| O89: Complications of anesthesia during the puerperium | 0.000 |
| T19: Foreign body in genitourinary tract | 0.000 |
| O64: Obstructed labor due to malposition and malpresentation of fetus | 0.000 |
| Q84: Other congenital malformations of integument | 0.000 |
| Q64: Other congenital malformations of urinary system | 0.000 |
| J64: Unspecified pneumoconiosis | 0.000 |
| J94: Other pleural conditions | 0.000 |
| N04: Nephrotic syndrome | 0.000 |
| I09: Other rheumatic heart diseases | 0.000 |
| D84: Other immunodeficiencies | 0.000 |
| O14: Pre-eclampsia | 0.000 |
| O04: Complications following (induced) termination of pregnancy | 0.000 |
| A54: Gonococcal infection | 0.000 |
| D04: Carcinoma in situ of skin | 0.000 |
| F94: Disorders of social functioning with onset specific to childhood and adolescence | 0.000 |
| E44: Protein-calorie malnutrition of moderate and mild degree | 0.000 |
| Q14: Congenital malformations of posterior segment of eye | 0.000 |
| B89: Unspecified parasitic disease | 0.000 |
| M45: Ankylosing spondylitis | 0.000 |
| J05: Acute obstructive laryngitis [croup] and epiglottitis | 0.000 |
| N85: Other noninflammatory disorders of uterus, except cervix | 0.000 |
| T13 | 0.000 |
| M05: Rheumatoid arthritis with rheumatoid factor | 0.000 |
| I15: Secondary hypertension | 0.000 |
| D15: Benign neoplasm of other and unspecified intrathoracic organs | 0.000 |
| R45: Symp.* involving emotional state | 0.000 |
| L25: Unspecified contact dermatitis | 0.000 |
| E75: Disorders of sphingolipid metabolism and other lipid storage disorders | 0.000 |
| K25: Gastric ulcer | 0.000 |
| R55: Syncope and collapse | 0.000 |
| J35: Chronic diseases of tonsils and adenoids | 0.000 |
| K60: Fissure and fistula of anal and rectal regions | 0.000 |
| Q70: Syndactyly | 0.000 |
| N75: Diseases of Bartholin's gland | 0.000 |
| R05: Cough | 0.000 |
| F15: Other stimulant related disorders | 0.000 |
| I85: Esophageal varices | 0.000 |
| K75: Other inflammatory liver diseases | 0.000 |
| B30: Viral conjunctivitis | 0.000 |
| F05: Delirium due to known physiological condition | 0.000 |
| H05: Disorders of orbit | 0.000 |
| H95: Intraoperative and postprocedural complications and disorders of ear and mastoid process | 0.000 |
| T85: Complications of other internal prosthetic devices, implants and grafts | 0.000 |
| S95: Injury of blood vessels at ankle and foot level | 0.000 |
| L05: Pilonidal cyst and sinus | 0.000 |
| L65: Other nonscarring hair loss | 0.000 |
| D42: Neoplasm of uncertain behavior of meninges | 0.000 |
| M85: Other disorders of bone density and structure | 0.000 |
| H70: Mastoiditis and related conditions | 0.000 |
| J14: Pneumonia due to Hemophilus influenzae | 0.000 |
| K85: Acute pancreatitis | 0.000 |
| L85: Other epidermal thickening | 0.000 |
| S85: Injury of blood vessels at lower leg level | 0.000 |
| S05: Injury of eye and orbit | 0.000 |
| S25: Injury of blood vessels of thorax | 0.000 |
| F55: Abuse of non-psychoactive substances | 0.000 |
| S65: Injury of blood vessels at wrist and hand level | 0.000 |
| K45: Other abdominal hernia | 0.000 |
| N45: Orchitis and epididymitis | 0.000 |
| E25: Adrenogenital disorders | 0.000 |
| O99: Other maternal diseases classifiable elsewhere but complicating pregnancy, childbirth and the puerperium | 0.000 |
| S35: Injury of blood vessels at abdomen, lower back and pelvis level | 0.000 |
| O25: Malnutrition in pregnancy, childbirth and the puerperium | 0.000 |
| D75: Other and unspecified diseases of blood and blood-forming organs | 0.000 |
| F65: Paraphilias | 0.000 |
| N15: Other renal tubulo-interstitial diseases | 0.000 |
| K05: Gingivitis and periodontal diseases | 0.000 |
| S55: Injury of blood vessels at forearm level | 0.000 |
| T56: Toxic effect of metals | 0.000 |
| S75: Injury of blood vessels at hip and thigh level | 0.000 |
| T25: Burn and corrosion of ankle and foot | 0.000 |
| O35: Maternal care for known or suspected fetal abnormality and damage | 0.000 |
| O90: Complications of the puerperium | 0.000 |
| O65: Obstructed labor due to maternal pelvic abnormality | 0.000 |
| Q85: Phakomatoses | 0.000 |
| N05: Unspecified nephritic syndrome | 0.000 |
| F25: Schizoaffective disorders | 0.000 |
| O15: Eclampsia | 0.000 |
| O05 | 0.000 |
| D05: Carcinoma in situ of breast | 0.000 |
| F95: Tic disorder | 0.000 |
| E45: Retarded development following protein-calorie malnutrition | 0.000 |
| Q15: Other congenital malformations of eye | 0.000 |
| M46: Other inflammatory spondylopathies | 0.000 |
| R16: Hepatomegaly and splenomegaly | 0.000 |
| M66: Spontaneous rupture of synovium and tendon | 0.000 |
| M06: Other rheumatoid arthritis | 0.000 |
| D16: Benign neoplasm of bone and articular cartilage | 0.000 |
| A06: Amebiasis | 0.000 |
| R46: Symp.* involving appearance and behavior | 0.000 |
| L26: Exfoliative dermatitis | 0.000 |
| E76: Disorders of glycosaminoglycan metabolism | 0.000 |
| R56: Convulsions | 0.000 |
| J46 | 0.000 |
| J36: Peritonsillar abscess | 0.000 |
| K61: Abscess of anal and rectal regions | 0.000 |
| Q71: Reduction defects of upper limb | 0.000 |
| N76: Other inflammation of vagina and vulva | 0.000 |
| R06: Abnormalities of breathing | 0.000 |
| E56: Other vitamin deficiencies | 0.000 |
| F16: Hallucinogen related disorders | 0.000 |
| I86: Varicose veins of other sites | 0.000 |
| H06 | 0.000 |
| T86: Complications of transplanted organs and tissue | 0.000 |
| S96: Injury of muscle and tendon at ankle and foot level | 0.000 |
| L66: Cicatricial alopecia [scarring hair loss] | 0.000 |
| I36: Nonrheumatic tricuspid valve disorders | 0.000 |
| D43: Neoplasm of uncertain behavior of brain and central nervous system | 0.000 |
| M86: Osteomyelitis | 0.000 |
| H71: Cholesteatoma of middle ear | 0.000 |
| J15: Bacterial pneumonia | 0.000 |
| K86: Other diseases of pancreas | 0.000 |
| S86: Injury of muscle, fascia and tendon at lower leg level | 0.000 |
| S06: Intracranial injury | 0.000 |
| S66: Injury of muscle, fascia and tendon at wrist and hand level | 0.000 |
| K46: Unspecified abdominal hernia | 0.000 |
| N46: Male infertility | 0.000 |
| R76: Other abnormal immunological findings in serum | 0.000 |
| E26: Hyperaldosteronism | 0.000 |
| S36: Injury of intra-abdominal organs | 0.000 |
| S16: Injury of muscle, fascia and tendon at neck level | 0.000 |
| O26: Maternal care for other conditions predominantly related to pregnancy | 0.000 |
| D76: Other specified diseases with participation of lymphoreticular and reticulohistiocytic tissue | 0.000 |
| R89: Abnormal findings in specimens from other organs, systems and tissues | 0.000 |
| H21: Other disorders of iris and ciliary body | 0.000 |
| F66: Other sexual disorders | 0.000 |
| T06 | 0.000 |
| K06: Other disorders of gingiva and edentulous alveolar ridge | 0.000 |
| S46: Injury of muscle, fascia and tendon at shoulder and upper arm level | 0.000 |
| S56: Injury of muscle, fascia and tendon at forearm level | 0.000 |
| S76: Injury of muscle, fascia and tendon at hip and thigh level | 0.000 |
| T26: Burn and corrosion confined to eye and adnexa | 0.000 |
| O36: Maternal care for other fetal problems | 0.000 |
| O91: Infections of breast associated with pregnancy, the puerperium and lactation | 0.000 |
| O66: Other obstructed labor | 0.000 |
| N06: Isolated proteinuria with specified morphological lesion | 0.000 |
| D86: Sarcoidosis | 0.000 |
| O16: Unspecified maternal hypertension | 0.000 |
| O06 | 0.000 |
| A56: Other sexually transmitted chlamydial diseases | 0.000 |
| D06: Carcinoma in situ of cervix uteri | 0.000 |
| A26: Erysipeloid | 0.000 |
| E46: Unspecified protein-calorie malnutrition | 0.000 |
| R17: Unspecified jaundice | 0.000 |
| N87: Dysplasia of cervix uteri | 0.000 |
| M07: Enteropathic arthropathies | 0.000 |
| A07: Other protozoal intestinal diseases | 0.000 |
| L27: Dermatitis due to substances taken internally | 0.000 |
| E77: Disorders of glycoprotein metabolism | 0.000 |
| K27: Peptic ulcer, site unspecified | 0.000 |
| R57: Shock | 0.000 |
| J47: Bronchiectasis | 0.000 |
| J37: Chronic laryngitis and laryngotracheitis | 0.000 |
| K62: Other diseases of anus and rectum | 0.000 |
| Q72: Reduction defects of lower limb | 0.000 |
| N77: Vulvovaginal ulceration and inflammation in diseases classified elsewhere | 0.000 |
| T73: Effects of other deprivation | 0.000 |
| E07: Other disorders of thyroid | 0.000 |
| K77: Liver disorders in diseases classified elsewhere | 0.000 |
| T87: Complications peculiar to reattachment and amputation | 0.000 |
| S97: Crushing injury of ankle and foot | 0.000 |
| B07: Viral warts | 0.000 |
| L67: Hair color and hair shaft abnormalities | 0.000 |
| I37: Nonrheumatic pulmonary valve disorders | 0.000 |
| D44: Neoplasm of uncertain behavior of endocrine glands | 0.000 |
| M87: Osteonecrosis | 0.000 |
| H72: Perforation of tympanic membrane | 0.000 |
| J16: Pneumonia due to other infectious organisms | 0.000 |
| K87: Disorders of gallbladder, biliary tract and pancreas in diseases classified elsewhere | 0.000 |
| L87: Transepidermal elimination disorders | 0.000 |
| S07: Crushing injury of head | 0.000 |
| S27: Injury of other and unspecified intrathoracic organs | 0.000 |
| S67: Crushing injury of wrist, hand and fingers | 0.000 |
| N47: Disorders of prepuce | 0.000 |
| R77: Other abnormalities of plasma proteins | 0.000 |
| A37: Whooping cough | 0.000 |
| I77: Other disorders of arteries and arterioles | 0.000 |
| E27: Other disorders of adrenal gland | 0.000 |
| S37: Injury of urinary and pelvic organs | 0.000 |
| D77: Other disorders of blood and blood-forming organs in diseases classified elsewhere | 0.000 |
| H22: Disorders of iris and ciliary body in diseases classified elsewhere | 0.000 |
| T07: Unspecified multiple injuries | 0.000 |
| K07 | 0.000 |
| S57: Crushing injury of elbow and forearm | 0.000 |
| T58: Toxic effect of carbon monoxide | 0.000 |
| O92: Other disorders of breast and disorders of lactation associated with pregnancy and the puerperium | 0.000 |
| Q87: Other specified congenital malformation syndromes affecting multiple systems | 0.000 |
| J67: Hypersensitivity pneumonitis due to organic dust | 0.000 |
| D07: Carcinoma in situ of other and unspecified genital organs | 0.000 |
| Q17: Other congenital malformations of ear | 0.000 |
| R18: Ascites | 0.000 |
| M68 | 0.000 |
| N88: Other noninflammatory disorders of cervix uteri | 0.000 |
| D18: Hemangioma and lymphangioma, any site | 0.000 |
| A08: Viral and other specified intestinal infections | 0.000 |
| L28: Lichen simplex chronicus and prurigo | 0.000 |
| K28: Gastrojejunal ulcer | 0.000 |
| R58: Hemorrhage | 0.000 |
| J38: Diseases of vocal cords and larynx | 0.000 |
| K63: Other diseases of intestine | 0.000 |
| Q73: Reduction defects of unspecified limb | 0.000 |
| E58: Dietary calcium deficiency | 0.000 |
| T74: Adult and child abuse, neglect and other maltreatment, confirmed | 0.000 |
| F18: Inhalant related disorders | 0.000 |
| I88: Nonspecific lymphadenitis | 0.000 |
| B33: Other viral diseases | 0.000 |
| T88: Other complications of surgical and medical care | 0.000 |
| S98: Traumatic amputation of ankle and foot | 0.000 |
| B08: Other viral infections characterized by skin and mucous membrane lesions | 0.000 |
| L08: Other local infections of skin and subcutaneous tissue | 0.000 |
| L68: Hypertrichosis | 0.000 |
| I38: Endocarditis, valve unspecified | 0.000 |
| H73: Other disorders of tympanic membrane | 0.000 |
| L88: Pyoderma gangrenosum | 0.000 |
| S68: Traumatic amputation of wrist, hand and fingers | 0.000 |
| N48: Other disorders of penis | 0.000 |
| R78: Findings of drugs and other substances, not normally found in blood | 0.000 |
| A38: Scarlet fever | 0.000 |
| I78: Diseases of capillaries | 0.000 |
| E28: Ovarian dysfunction | 0.000 |
| O28: Abnormal findings on antenatal screening of mother | 0.000 |
| F68: Other disorders of adult personality and behavior | 0.000 |
| K08: Other disorders of teeth and supporting structures | 0.000 |
| C08: Malignant neoplasm of other and unspecified major salivary glands | 0.000 |
| S58: Traumatic amputation of elbow and forearm | 0.000 |
| T59: Toxic effect of other gases, fumes and vapors | 0.000 |
| O68: Labor and delivery complicated by abnormality of fetal acid-base balance | 0.000 |
| J68: Respiratory conditions due to inhalation of chemicals, gases, fumes and vapors | 0.000 |
| F28: Other psychotic disorder not due to a substance or known physiological condition | 0.000 |
| O08: Complications following ectopic and molar pregnancy | 0.000 |
| F98: Other behavioral and emotional disorders with onset usually occurring in childhood and adolescence | 0.000 |
| A28: Other zoonotic bacterial diseases | 0.000 |
| Q18: Other congenital malformations of face and neck | 0.000 |
| M49: Spondylopathies in diseases classified elsewhere | 0.000 |
| R19: Other symp.* involving the digestive system and abdomen | 0.000 |
| M09 | 0.000 |
| A09: Infectious gastroenteritis and colitis, unspecified | 0.000 |
| L29: Pruritus | 0.000 |
| R59: Enlarged lymph nodes | 0.000 |
| J39: Other diseases of upper respiratory tract | 0.000 |
| K64: Hemorrhoids and perianal venous thrombosis | 0.000 |
| R09: Other symp.* involving the circulatory and respiratory system | 0.000 |
| E59: Dietary selenium deficiency | 0.000 |
| T75: Other and unspecified effects of other external causes | 0.000 |
| F19: Other psychoactive substance related disorders | 0.000 |
| B44: Aspergillosis | 0.000 |
| B34: Viral infection of unspecified site | 0.000 |
| F09: Unspecified mental disorder due to known physiological condition | 0.000 |
| S99: Other and unspecified injuries of ankle and foot | 0.000 |
| B09: Unspecified viral infection characterized by skin and mucous membrane lesions | 0.000 |
| D46: Myelodysplastic syndromes | 0.000 |
| M89: Other disorders of bone | 0.000 |
| H74: Other disorders of middle ear mastoid | 0.000 |
| S89: Other and unspecified injuries of lower leg | 0.000 |
| S09: Other and unspecified injuries of head | 0.000 |
| S29: Other and unspecified injuries of thorax | 0.000 |
| F59: Unspecified behavioral syndromes associated with physiological disturbances and physical factors | 0.000 |
| S69: Other and unspecified injuries of wrist, hand and finger(s) | 0.000 |
| N49: Inflammatory disorders of male genital organs | 0.000 |
| R79: Other abnormal findings of blood chemistry | 0.000 |
| A39: Meningococcal infection | 0.000 |
| I79: Disorders of arteries, arterioles and capillaries in diseases classified elsewhere | 0.000 |
| E29: Testicular dysfunction | 0.000 |
| S39: Other and unspecified injuries of abdomen, lower back, pelvis and external genitals | 0.000 |
| F69: Unspecified disorder of adult personality and behavior | 0.000 |
| K09: Cysts of oral region | 0.000 |
| S49: Other and unspecified injuries of shoulder and upper arm | 0.000 |
| C09: Malignant neoplasm of tonsil | 0.000 |
| T60: Toxic effect of pesticides | 0.000 |
| S79: Other and unspecified injuries of hip and thigh | 0.000 |
| T29 | 0.000 |
| O69: Labor and delivery complicated by umbilical cord complications | 0.000 |
| Q89: Other congenital malformations | 0.000 |
| J69: Pneumonitis due to solids and liquids | 0.000 |
| F29: Unspecified psychosis not due to a substance or known physiological condition | 0.000 |
| D89: Other disorders involving the immune mechanism | 0.000 |
| A59: Trichomoniasis | 0.000 |
| D09: Carcinoma in situ of other and unspecified sites | 0.000 |
| M70: Soft tissue disorders related to use, overuse and pressure | 0.000 |
| N90: Other noninflammatory disorders of vulva and perineum | 0.000 |
| M10: Gout | 0.000 |
| E80: Disorders of porphyrin and bilirubin metabolism | 0.000 |
| Q75: Other congenital malformations of skull and face bones | 0.000 |
| E60: Dietary zinc deficiency | 0.000 |
| L70: Acne | 0.000 |
| I40: Acute myocarditis | 0.000 |
| D47: Other neoplasms of uncertain behavior of lymphoid, hematopoietic and related tissue | 0.000 |
| M90: Osteopathies in diseases classified elsewhere | 0.000 |
| L90: Atrophic disorders of skin | 0.000 |
| N50: Other and unspecified disorders of male genital organs | 0.000 |
| A40: Streptococcal sepsis | 0.000 |
| K10 | 0.000 |
| C10: Malignant neoplasm of oropharynx | 0.000 |
| T61: Toxic effect of noxious substances eaten as seafood | 0.000 |
| T30: Burn and corrosion, body region unspecified | 0.000 |
| O40: Polyhydramnios | 0.000 |
| O70: Perineal laceration during delivery | 0.000 |
| J70: Respiratory conditions due to other external agents | 0.000 |
| A60: Anogenital herpesviral [herpes simplex] infections | 0.000 |
| M71: Other bursopathies | 0.000 |
| M11: Other crystal arthropathies | 0.000 |
| D21: Other benign neoplasms of connective and other soft tissue | 0.000 |
| R61: Generalized hyperhidrosis | 0.000 |
| Q76: Congenital malformations of spine and bony thorax | 0.000 |
| E61: Deficiency of other nutrient elements | 0.000 |
| B46: Zygomycosis | 0.000 |
| I41: Myocarditis in diseases classified elsewhere | 0.000 |
| D48: Neoplasm of uncertain behavior of other and unspecified sites | 0.000 |
| M91: Juvenile osteochondrosis of hip and pelvis | 0.000 |
| L91: Hypertrophic disorders of skin | 0.000 |
| N51: Disorders of male genital organs in diseases classified elsewhere | 0.000 |
| A41: Other sepsis | 0.000 |
| E31: Polyglandular dysfunction | 0.000 |
| K11: Diseases of salivary glands | 0.000 |
| C11: Malignant neoplasm of nasopharynx | 0.000 |
| T62: Toxic effect of other noxious substances eaten as food | 0.000 |
| T31: Burns classified according to extent of body surface involved | 0.000 |
| O41: Other disorders of amniotic fluid and membranes | 0.000 |
| O71: Other obstetric trauma | 0.000 |
| M72: Fibroblastic disorders | 0.000 |
| M12: Other and unspecified arthropathy | 0.000 |
| L72: Follicular cysts of skin and subcutaneous tissue | 0.000 |
| I42: Cardiomyopathy | 0.000 |
| M92: Other juvenile osteochondrosis | 0.000 |
| L92: Granulomatous disorders of skin and subcutaneous tissue | 0.000 |
| A42: Actinomycosis | 0.000 |
| E32: Diseases of thymus | 0.000 |
| K12: Stomatitis and related lesions | 0.000 |
| T63: Toxic effect of contact with venomous animals and plants | 0.000 |
| O42: Premature rupture of membranes | 0.000 |
| O72: Postpartum hemorrhage | 0.000 |
| M73 | 0.000 |
| N93: Other abnormal uterine and vaginal bleeding | 0.000 |
| D23: Other benign neoplasms of skin | 0.000 |
| E83: Disorders of mineral metabolism | 0.000 |
| R63: Symp.* concerning food and fluid intake | 0.000 |
| Q78: Other osteochondrodysplasias | 0.000 |
| E63: Other nutritional deficiencies | 0.000 |
| B48: Other mycoses | 0.000 |
| L73: Other follicular disorders | 0.000 |
| I43: Cardiomyopathy in diseases classified elsewhere | 0.000 |
| M93: Other osteochondropathies | 0.000 |
| L93: Lupus erythematosus | 0.000 |
| K13: Other diseases of lip and oral mucosa | 0.000 |
| C13: Malignant neoplasm of hypopharynx | 0.000 |
| O43: Placental disorders | 0.000 |
| O73: Retained placenta and membranes, without hemorrhage | 0.000 |
| A63: Other predominantly sexually transmitted diseases | 0.000 |
| M14: Arthropathies in other diseases classified elsewhere | 0.000 |
| D24: Benign neoplasm of breast | 0.000 |
| R64: Cachexia | 0.000 |
| Q79: Congenital malformations of musculoskeletal system | 0.000 |
| E64: Sequelae of malnutrition and other nutritional deficiencies | 0.000 |
| B49: Unspecified mycosis | 0.000 |
| L74: Eccrine sweat disorders | 0.000 |
| I44: Atrioventricular and left bundle-branch block | 0.000 |
| L94: Other localized connective tissue disorders | 0.000 |
| E34: Other endocrine disorders | 0.000 |
| K14: Diseases of tongue | 0.000 |
| T65: Toxic effect of other and unspecified substances | 0.000 |
| O44: Placenta previa | 0.000 |
| O74: Complications of anesthesia during labor and delivery | 0.000 |
| A64: Unspecified sexually transmitted disease | 0.000 |
| M15: Polyosteoarthritis | 0.000 |
| E85: Amyloidosis | 0.000 |
| R65: Symp.* specifically associated with systemic inflammation and infection | 0.000 |
| L95: Vasculitis limited to skin | 0.000 |
| E35: Disorders of endocrine glands in diseases classified elsewhere | 0.000 |
| C15: Malignant neoplasm of esophagus | 0.000 |
| O75: Other complications of labor and delivery | 0.000 |
| M76: Enthesopathies, lower limb, excluding foot | 0.000 |
| N96: Recurrent pregnancy loss | 0.000 |
| D26: Other benign neoplasms of uterus | 0.000 |
| I46: Cardiac arrest | 0.000 |
| A46: Erysipelas | 0.000 |
| C16: Malignant neoplasm of stomach | 0.000 |
| O46: Antepartum hemorrhage | 0.000 |
| N97: Female infertility | 0.000 |
| D27: Benign neoplasm of ovary | 0.000 |
| I47: Paroxysmal tachycardia | 0.000 |
| L97: Non-pressure chronic ulcer of lower limb | 0.000 |
| C17: Malignant neoplasm of small intestine | 0.000 |
| O47: False labor | 0.000 |
| N98: Complications associated with artificial fertilization | 0.000 |
| M18: Osteoarthritis of first carpometacarpal joint | 0.000 |
| D28: Benign neoplasm of other and unspecified female genital organs | 0.000 |
| E88: Other and unspecified metabolic disorders | 0.000 |
| R68: Other general symp.* | 0.000 |
| L98: Other disorders of skin and subcutaneous tissue | 0.000 |
| A48: Other bacterial diseases | 0.000 |
| C18: Malignant neoplasm of colon | 0.000 |
| O48: Late pregnancy | 0.000 |
| R69: Illness, unspecified | 0.000 |
| I49: Other cardiac arrhythmias | 0.000 |
| L99: Other disorders of skin and subcutaneous tissue in diseases classified elsewhere | 0.000 |
| A49: Bacterial infection of unspecified site | 0.000 |
| C19: Malignant neoplasm of rectosigmoid junction | 0.000 |
| D30: Benign neoplasm of urinary organs | 0.000 |
| E90 | 0.000 |
| C20: Malignant neoplasm of rectum | 0.000 |
| D31: Benign neoplasm of eye and adnexa | 0.000 |
| I51: Complications and ill-defined descriptions of heart disease | 0.000 |
| C21: Malignant neoplasm of anus and anal canal | 0.000 |
| D32: Benign neoplasm of meninges | 0.000 |
| C22: Malignant neoplasm of liver and intrahepatic bile ducts | 0.000 |
| D33: Benign neoplasm of brain and other parts of central nervous system | 0.000 |
| M24: Other specific joint derangements | 0.000 |
| C24: Malignant neoplasm of other and unspecified parts of biliary tract | 0.000 |
| C25: Malignant neoplasm of pancreas | 0.000 |
| D36: Benign neoplasm of other and unspecified sites | 0.000 |
| C26: Malignant neoplasm of other and ill-defined digestive organs | 0.000 |
| C30: Malignant neoplasm of nasal cavity and middle ear | 0.000 |
| C32: Malignant neoplasm of larynx | 0.000 |
| C34: Malignant neoplasm of bronchus and lung | 0.000 |
| C38: Malignant neoplasm of heart, mediastinum and pleura | 0.000 |
| C40: Malignant neoplasm of bone and articular cartilage of limbs | 0.000 |
| C41: Malignant neoplasm of bone and articular cartilage of other and unspecified sites | 0.000 |
| C43: Malignant melanoma of skin | 0.000 |
| C45: Mesothelioma | 0.000 |
| C46: Kaposi's sarcoma | 0.000 |
| C47: Malignant neoplasm of peripheral nerves and autonomic nervous system | 0.000 |
| C49: Malignant neoplasm of other connective and soft tissue | 0.000 |
| C51: Malignant neoplasm of vulva | 0.000 |
| C53: Malignant neoplasm of cervix uteri | 0.000 |
| C54: Malignant neoplasm of corpus uteri | 0.000 |
| C55: Malignant neoplasm of uterus, part unspecified | 0.000 |
| C56: Malignant neoplasm of ovary | 0.000 |
| C57: Malignant neoplasm of other and unspecified female genital organs | 0.000 |
| C58: Malignant neoplasm of placenta | 0.000 |
| C60: Malignant neoplasm of penis | 0.000 |
| C62: Malignant neoplasm of testis | 0.000 |
| C66: Malignant neoplasm of ureter | 0.000 |
| C67: Malignant neoplasm of bladder | 0.000 |
| C68: Malignant neoplasm of other and unspecified urinary organs | 0.000 |
| C69: Malignant neoplasm of eye and adnexa | 0.000 |
| C70: Malignant neoplasm of meninges | 0.000 |
| C71: Malignant neoplasm of brain | 0.000 |
| C72: Malignant neoplasm of spinal cord, cranial nerves and other parts of central nervous system | 0.000 |
| C73: Malignant neoplasm of thyroid gland | 0.000 |
| C75: Malignant neoplasm of other endocrine glands and related structures | 0.000 |
| C76: Malignant neoplasm of other and ill-defined sites | 0.000 |
| C79: Secondary malignant neoplasm of other and unspecified sites | 0.000 |
| C80: Malignant neoplasm without specification of site | 0.000 |
| C82: Follicular lymphoma | 0.000 |
| C83: Non-follicular lymphoma | 0.000 |
| C84: Mature T/NK-cell lymphomas | 0.000 |
| C85: Other specified and unspecified types of non-Hodgkin lymphoma | 0.000 |
| C86: Other specified types of T/NK-cell lymphoma | 0.000 |
| C90: Multiple myeloma and malignant plasma cell neoplasms | 0.000 |
| C91: Lymphoid leukemia | 0.000 |
| C92: Myeloid leukemia | 0.000 |
| C94: Other leukemias of specified cell type | 0.000 |
| C95: Leukemia of unspecified cell type | 0.000 |
| C96: Other and unspecified malignant neoplasms of lymphoid, hematopoietic and related tissue | 0.000 |
| C97 | 0.000 |

### Table S12: The predictive performances obtained for each model configuration (varying observation durations and missing value percentages) based on the area under the precision-recall curve (AUPR) values over 50 replications.

| **Replication** | **4years 0% NA** | **4years 10% NA** | **4years 30% NA** | **5years 0% NA** | **5years 10% NA** | **5years 30% NA** | **6years 0% NA** | **6years 10% NA** | **6years 30% NA** |
| --- | --- | --- | --- | --- | --- | --- | --- | --- | --- |
| 1 | 0.813 | 0.827 | 0.831 | 0.868 | 0.880 | 0.865 | 0.837 | 0.875 | 0.880 |
| 2 | 0.833 | 0.823 | 0.831 | 0.870 | 0.847 | 0.876 | 0.860 | 0.872 | 0.873 |
| 3 | 0.825 | 0.824 | 0.821 | 0.876 | 0.872 | 0.876 | 0.845 | 0.872 | 0.876 |
| 4 | 0.802 | 0.827 | 0.826 | 0.877 | 0.871 | 0.878 | 0.831 | 0.864 | 0.870 |
| 5 | 0.805 | 0.827 | 0.819 | 0.877 | 0.871 | 0.876 | 0.841 | 0.874 | 0.872 |
| 6 | 0.798 | 0.826 | 0.826 | 0.873 | 0.870 | 0.870 | 0.857 | 0.882 | 0.875 |
| 7 | 0.822 | 0.830 | 0.812 | 0.875 | 0.881 | 0.875 | 0.844 | 0.874 | 0.871 |
| 8 | 0.814 | 0.824 | 0.827 | 0.863 | 0.875 | 0.877 | 0.841 | 0.870 | 0.873 |
| 9 | 0.805 | 0.831 | 0.824 | 0.888 | 0.874 | 0.876 | 0.860 | 0.875 | 0.873 |
| 10 | 0.840 | 0.826 | 0.824 | 0.878 | 0.875 | 0.872 | 0.870 | 0.880 | 0.869 |
| 11 | 0.804 | 0.819 | 0.819 | 0.859 | 0.872 | 0.873 | 0.833 | 0.871 | 0.871 |
| 12 | 0.833 | 0.826 | 0.822 | 0.865 | 0.869 | 0.878 | 0.847 | 0.879 | 0.866 |
| 13 | 0.824 | 0.828 | 0.829 | 0.860 | 0.875 | 0.873 | 0.834 | 0.878 | 0.874 |
| 14 | 0.823 | 0.826 | 0.819 | 0.873 | 0.874 | 0.867 | 0.851 | 0.879 | 0.869 |
| 15 | 0.809 | 0.823 | 0.817 | 0.854 | 0.872 | 0.874 | 0.854 | 0.870 | 0.868 |
| 16 | 0.820 | 0.828 | 0.823 | 0.877 | 0.879 | 0.873 | 0.885 | 0.877 | 0.879 |
| 17 | 0.816 | 0.809 | 0.823 | 0.862 | 0.873 | 0.870 | 0.851 | 0.874 | 0.876 |
| 18 | 0.816 | 0.818 | 0.831 | 0.857 | 0.880 | 0.882 | 0.838 | 0.877 | 0.883 |
| 19 | 0.813 | 0.821 | 0.815 | 0.894 | 0.868 | 0.876 | 0.854 | 0.877 | 0.876 |
| 20 | 0.815 | 0.821 | 0.837 | 0.882 | 0.869 | 0.875 | 0.841 | 0.871 | 0.875 |
| 21 | 0.786 | 0.826 | 0.828 | 0.877 | 0.876 | 0.873 | 0.853 | 0.871 | 0.874 |
| 22 | 0.793 | 0.818 | 0.827 | 0.856 | 0.867 | 0.873 | 0.854 | 0.875 | 0.870 |
| 23 | 0.821 | 0.829 | 0.831 | 0.890 | 0.877 | 0.870 | 0.868 | 0.880 | 0.875 |
| 24 | 0.822 | 0.828 | 0.827 | 0.885 | 0.880 | 0.874 | 0.860 | 0.871 | 0.873 |
| 25 | 0.817 | 0.820 | 0.830 | 0.892 | 0.875 | 0.880 | 0.809 | 0.878 | 0.880 |
| 26 | 0.811 | 0.819 | 0.826 | 0.871 | 0.877 | 0.878 | 0.842 | 0.878 | 0.877 |
| 27 | 0.825 | 0.820 | 0.823 | 0.863 | 0.875 | 0.877 | 0.849 | 0.880 | 0.876 |
| 28 | 0.821 | 0.825 | 0.819 | 0.876 | 0.877 | 0.880 | 0.822 | 0.869 | 0.872 |
| 29 | 0.794 | 0.828 | 0.819 | 0.876 | 0.882 | 0.873 | 0.848 | 0.877 | 0.878 |
| 30 | 0.829 | 0.826 | 0.825 | 0.883 | 0.870 | 0.875 | 0.858 | 0.871 | 0.876 |
| 31 | 0.801 | 0.832 | 0.813 | 0.885 | 0.874 | 0.879 | 0.822 | 0.871 | 0.881 |
| 32 | 0.809 | 0.827 | 0.829 | 0.874 | 0.881 | 0.872 | 0.855 | 0.874 | 0.869 |
| 33 | 0.817 | 0.829 | 0.833 | 0.868 | 0.878 | 0.872 | 0.848 | 0.883 | 0.875 |
| 34 | 0.801 | 0.828 | 0.825 | 0.902 | 0.876 | 0.874 | 0.825 | 0.882 | 0.872 |
| 35 | 0.813 | 0.816 | 0.830 | 0.874 | 0.874 | 0.877 | 0.827 | 0.877 | 0.874 |
| 36 | 0.793 | 0.824 | 0.817 | 0.877 | 0.883 | 0.873 | 0.839 | 0.874 | 0.876 |
| 37 | 0.822 | 0.829 | 0.825 | 0.877 | 0.875 | 0.876 | 0.858 | 0.879 | 0.876 |
| 38 | 0.834 | 0.825 | 0.824 | 0.880 | 0.877 | 0.879 | 0.837 | 0.879 | 0.869 |
| 39 | 0.807 | 0.828 | 0.822 | 0.866 | 0.884 | 0.873 | 0.846 | 0.878 | 0.874 |
| 40 | 0.807 | 0.819 | 0.824 | 0.882 | 0.875 | 0.875 | 0.859 | 0.877 | 0.877 |
| 41 | 0.808 | 0.829 | 0.826 | 0.868 | 0.880 | 0.873 | 0.856 | 0.871 | 0.871 |
| 42 | 0.800 | 0.830 | 0.820 | 0.881 | 0.875 | 0.868 | 0.837 | 0.879 | 0.874 |
| 43 | 0.816 | 0.814 | 0.815 | 0.880 | 0.880 | 0.873 | 0.867 | 0.871 | 0.873 |
| 44 | 0.796 | 0.812 | 0.808 | 0.854 | 0.874 | 0.875 | 0.865 | 0.877 | 0.876 |
| 45 | 0.822 | 0.839 | 0.821 | 0.888 | 0.881 | 0.871 | 0.852 | 0.875 | 0.880 |
| 46 | 0.825 | 0.818 | 0.826 | 0.882 | 0.847 | 0.869 | 0.862 | 0.875 | 0.869 |
| 47 | 0.823 | 0.824 | 0.815 | 0.871 | 0.874 | 0.878 | 0.863 | 0.871 | 0.871 |
| 48 | 0.811 | 0.825 | 0.819 | 0.859 | 0.881 | 0.879 | 0.837 | 0.873 | 0.879 |
| 49 | 0.812 | 0.822 | 0.834 | 0.858 | 0.880 | 0.883 | 0.853 | 0.874 | 0.877 |
| 50 | 0.818 | 0.833 | 0.829 | 0.885 | 0.883 | 0.873 | 0.832 | 0.873 | 0.878 |
| Average | 0.814 | 0.825 | 0.824 | 0.874 | 0.875 | 0.875 | 0.848 | 0.875 | 0.874 |

[1]. Lundberg S. A unified approach to interpreting model predictions. *arXiv preprint arXiv:170507874*. 2017.
